# Effects of a Multimodal Transitional Care Intervention in Patients at High Risk of Readmission: The TARGET-READ Randomized Clinical Trial

**DOI:** 10.1001/jamainternmed.2023.0791

**Published:** 2023-05-01

**Authors:** Jacques Donzé, Gregor John, Daniel Genné, Marco Mancinetti, Alexandre Gouveia, Marie Méan, Lukas Bütikofer, Drahomir Aujesky, Jeffrey Schnipper

**Affiliations:** 1 Department of Medicine, Neuchâtel Hospital Network, Neuchâtel, Switzerland; 2 Division of Internal Medicine, Inselspital, Bern University Hospital, Bern, Switzerland; 3 Division of Internal Medicine, Lausanne University Hospital (CHUV) and University of Lausanne, Lausanne, Switzerland; 4 Brigham and Women’s Hospital, Harvard Medical School, Boston, Massachusetts; 5 Department of Internal Medicine, Geneva University Hospitals (HUG), Geneva, Switzerland; 6 Geneva University, Geneva, Switzerland; 7 Department of Internal Medicine, Bienne Hospital Center, Bienne, Switzerland; 8 Department of Internal Medicine, Hôpital cantonal de Fribourg, Villars-sur-Glâne, Switzerland; 9 Medical Education Unit, University of Fribourg, Switzerland; 10 Department of Ambulatory Care, Center for Primary Care and Public Health (Unisanté), University of Lausanne, Lausanne, Switzerland; 11 Division of Internal Medicine, Lausanne University Hospital (CHUV), Lausanne, Switzerland; 12 Clinical Trials Unit, University of Bern, Bern, Switzerland; 13 Department of Internal Medicine, Bern University Hospital and University of Bern, Bern, Switzerland

## Abstract

**Importance:**

Hospital readmissions are frequent, costly, and sometimes preventable. Although these issues have been well publicized and incentives to reduce them introduced, the best interventions for reducing readmissions remain unclear.

**Objectives:**

To evaluate the effects of a multimodal transitional care intervention targeting patients at high risk of hospital readmission on the composite outcome of 30-day unplanned readmission or death.

**Design, Setting, and Participants:**

A single-blinded, multicenter randomized clinical trial was conducted from April 2018 to January 2020, with a 30-day follow-up in 4 medium-to-large–sized teaching hospitals in Switzerland. Participants were consecutive patients discharged from general internal medicine wards and at higher risk of unplanned readmission based on their simplified HOSPITAL score (≥4 points). Data were analyzed between April and September 2022.

**Interventions:**

The intervention group underwent systematic medication reconciliation, a 15-minute patient education session with teach-back, a planned first follow-up visit with their primary care physician, and postdischarge follow-up telephone calls from the study team at 3 and 14 days. The control group received usual care from their hospitalist, plus a 1-page standard study information sheet.

**Main Outcomes and Measures:**

Thirty-day postdischarge unplanned readmission or death.

**Results:**

A total of 1386 patients were included with a mean (SD) age of 72 (14) years; 712 (51%) were male. The composite outcome of 30-day unplanned readmission or death was 21% (95% CI, 18% to 24%) in the intervention group and 19% (95% CI, 17% to 22%) in the control group. The intention-to-treat analysis risk difference was 1.7% (95% CI, −2.5% to 5.9%; *P* = .44). There was no evidence of any intervention effects on time to unplanned readmission or death, postdischarge health care use, patient satisfaction with the quality of their care transition, or readmission costs.

**Conclusions and Relevance:**

In this randomized clinical trial, use of a standardized multimodal care transition intervention targeting higher-risk patients did not significantly decrease the risks of 30-day postdischarge unplanned readmission or death; it demonstrated the difficulties in preventing hospital readmissions, even when multimodal interventions specifically target higher-risk patients.

**Trial Registration:**

ClinicalTrials.gov Identifier: NCT03496896

## Key Points

**Question:**

Could a transitional care intervention targeting higher-risk medical patients reduce the risk of 30-day unplanned hospital readmission or death?

**Findings:**

In this randomized clinical trial including 1386 patients at a high risk of unplanned readmissions across 4 hospitals in Switzerland, no statistical difference in the composite outcome of 30-day unplanned readmission or death between the intervention and control groups was found. There was no evidence of any intervention effects on postdischarge health care use, patient satisfaction with the quality of their care transition, or readmission costs.

**Meaning:**

Results of this study suggest that the difficulties in preventing hospital readmissions continue, even when using multimodal interventions targeting higher-risk patients.

## Teaser

This randomized clinical trial evaluates the effects of a transitional care intervention targeting higher-risk medical patients using a composite outcome of 30-day unplanned readmission or death.

## Introduction

Many complications can occur when patients are discharged from hospitals to ambulatory settings; these may lead to unnecessary patient distress and costly hospital readmissions.^1,2^ It is estimated that 30% of readmissions are preventable^3^ and approximately 50% are potentially preventable.^4,5^ For these reasons, readmissions have received increasing attention from policy makers.^6,7^ Although issues surrounding readmissions and the quality of discharge processes have been identified previously, with incentives established to reduce readmissions, there have been few randomized clinical trials (RCTs) examining the effects of transitional care interventions on hospital readmissions and other patient-relevant outcomes.^8^

Although all patients deserve high-quality discharge processes (eg, timely handoff and follow-up appointments), more complex and costly interventions, like postdischarge telephone calls or home-based transition coaching, could specifically target those patients who are most likely to benefit from them.

Fewer than 20% of readmissions are due to the same specific acute diagnosis responsible for the index hospital admission.^2,9^ Indeed, patients are frequently readmitted due to complications from an underlying chronic condition.^10^ This suggests that postdischarge care should focus more on underlying comorbidities that could cause new acute complications and not just on the primary index hospital admission diagnosis. It also highlights the importance of adequately educating patients about how to self-manage their illnesses, improving care coordination between hospitals and outpatient settings, and promoting active patient follow-up during the high-risk period of the first weeks after discharge.

We evaluated the effects of a transitional care intervention targeting higher-risk medical patients using a composite outcome of 30-day unplanned readmission or death in a single-blinded, multicenter RCT. We also explored the effect of the intervention on the time to readmission or death, postdischarge health care use, patients’ perspectives on the quality of their care transition, and costs.

## Methods

**Figure 1.**
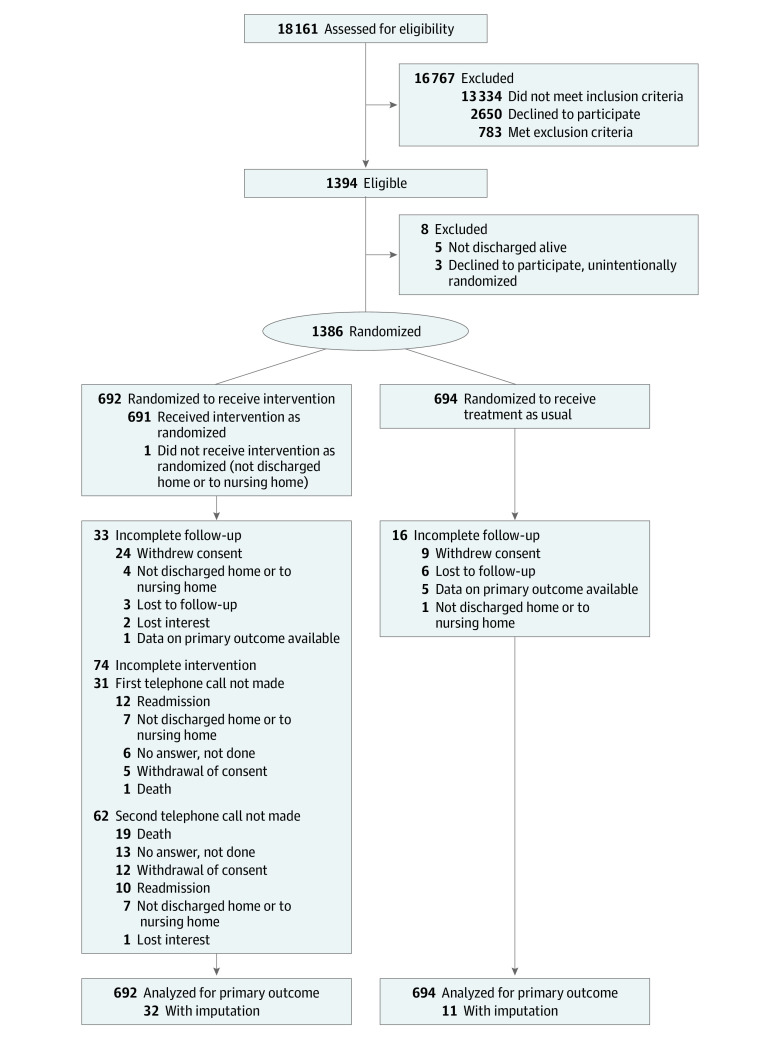
Patient Flow

In this RCT, patients at a higher risk of unplanned readmissions, discharged from the general internal medicine wards of 4 medium-to-large–sized teaching hospitals in Switzerland from April 2018 to January 2020, were randomized 1:1 in parallel groups, receiving either a standardized multimodal care transition from a trained team of discharge nurses or usual care (Figure 1). Data were analyzed between April and September 2022. Readmissions and overall mortality at 30 days postdischarge were recorded. The study followed the Consolidated Standards of Reporting Trials (CONSORT) reporting guideline, was approved by each participating hospital center’s ethics committee, and was coordinated by Bern University Hospital, whose clinical trials unit managed, monitored, and analyzed the data. The local study nurse at each site handled the patient informed consent. A signed copy was given to the patient, and another one was kept in the record at each site. The trial protocol is available in Supplement 1.

### Settings and Population

All consecutive adult patients (aged ≥18 years) admitted to the participating hospitals’ general internal medicine departments were screened for eligibility. They were eligible if their hospital stay lasted at least 1 day, they faced a higher risk of readmission (simplified HOSPITAL score ≥4 points), and they were planned to be discharged home or to a nursing home. The simplified HOSPITAL score (0-3: readmission unlikely; 4 or more: readmission likely) is an internationally validated clinical prognostic model that accurately predicts unplanned readmission after hospital discharge.^11,12^ It uses 6 variables readily available at discharge (eTable 1 in Supplement 2). We excluded patients previously enrolled in this study, living outside Switzerland, unable to speak German or French, without a telephone, or who declined or could not sign the informed consent form.

### Randomization and Blinding

All participants enrolled were centrally randomized to either the intervention or usual care groups using a computer-generated randomization list stratified by discharge site and their readmission-risk category per their simplified HOSPITAL score (4–5 vs ≥6 points), with randomly varying block sizes of 2, 4, and 6 patients. Given the nature of the intervention, blinding the patients or the discharge team was impossible, but the study nurses collecting outcomes data were blinded to group allocation.

### Intervention

**Figure 2.**
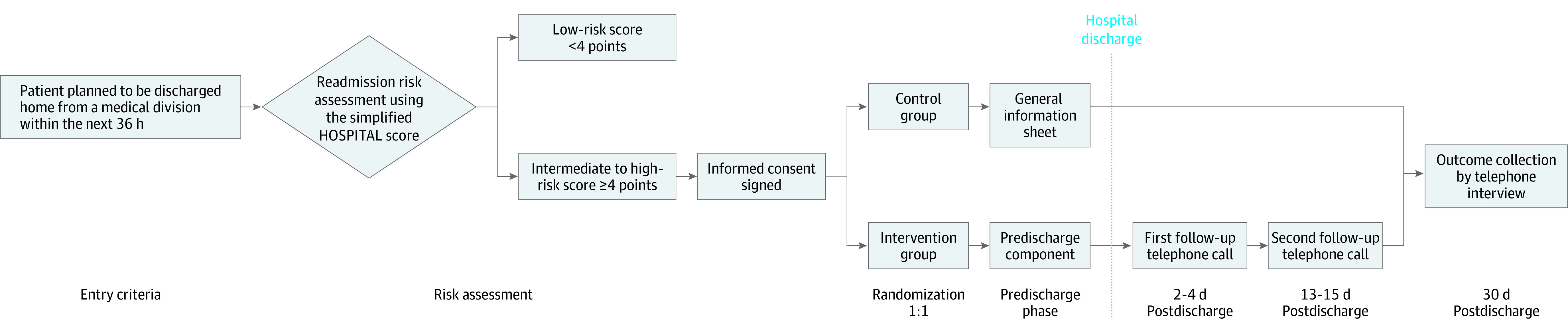
Intervention Study Overview The simplified HOSPITAL score ranges are 0-4 indicating readmission unlikely and 5 or more indicating readmission likely.

The intervention group received a standardized care transition intervention (Figure 2) from a team of trained discharge nurses consisting of 1 predischarge component and 2 postdischarge follow-up telephone calls on days 3 (±1) and 14 (±1). The predischarge component included identification of medication discrepancies in admission and/or discharge lists, a 15-minute patient education session about the patient’s main diseases using teach-back to confirm their understanding, and planning for the first postdischarge follow-up visit with their primary care physician (PCP) within 7 days of discharge. Patients receiving the intervention also received educational materials, including clear pragmatic advice on disease management, and a 1- to 2-page document containing information about their chronic diseases. These documents on most common chronic conditions were reviewed and adapted by a group composed of 2 patients, a PCP, 1 hospitalist, 1 pharmacist, and 1 care coordinator. Two follow-up telephone calls involved reinforcing the previous patient education, restating the importance of promptly consulting a PCP if needed, reviewing the patient’s medication list, assessing potential adverse drug events, and verifying that the hospital medical team had sent a timely discharge summary to the PCP.

Patients in the control group received usual care from their managing hospitalist plus a 1-page study information sheet (Figure 2). Usual care discharge procedures were similar in all 4 participating hospitals (eTable 2 in Supplement 2), without PCP follow-up visits scheduled at discharge, standardized patient education with teach-back, or follow-up postdischarge telephone calls.

### Outcomes and Measurements

The primary composite outcome was the number of patients discharged alive who had an unplanned readmission or died from any cause within 30 days of discharge. The secondary outcomes at 30 days were the individual components of the primary outcome: time to readmission or death (whichever came first), the main cause of readmission, postdischarge health care use (number of unplanned or planned readmissions, emergency department visits, and PCP visits), patient satisfaction with discharge (measured using the 3-item Care Transitions Measure or CTM-3^13^), and readmission costs based on diagnosis related group.

Unplanned readmission was defined as any nonelective hospitalization to any division of any acute care hospital. In contrast, elective hospitalization was defined as a nonurgent hospitalization scheduled at least 1 day before the admission day. Cause of death and readmission were collected from Switzerland’s national death registry and participating hospitals.

Data on outcomes at 30 days were collected using telephone interviews by trained study nurses who did not belong to any of the discharge teams. They had access to medical records to complete or ascertain patient-reported outcomes. PCPs were also contacted to complete any necessary information.

### Statistical Analyses

We assumed that the intervention would result in a 25% relative reduction in readmissions or death. With an expected 30-day readmission or death rate of 27% for patients at high risk,^14^ we calculated that a sample size of 1380 patients (690 in each group) was needed for a power of 80% at an α level of 5%, and an expected 10% loss to follow-up based on a χ^2^ test.

The primary analysis included all participants discharged alive, analyzed according to the treatment they were assigned at randomization (intention-to-treat), and adjusted for the stratification factors used at randomization (discharge site and simplified HOSPITAL score, 4–5 vs ≥6 points). The between-group comparison of the proportions of unplanned readmissions or deaths within 30 days (primary outcome) used a stratified Mantel-Haenszel risk difference with a 2-sided 95% CI calculated using the Klingenberg procedure^15^ and a Cochran-Mantel-Haenszel test. Death and satisfaction with discharge were analyzed in the same way. Each group’s risk of 30-day unplanned readmissions was estimated using the cumulative incidence function with death as a competing event, calculated from flexible parametric survival models.^2,16^ Between-group time to unplanned readmission or death was compared using a log-rank test, and the restricted mean survival time truncated at 30 days was calculated from flexible parametric survival models.^17^ The intervention and control groups’ health care use were compared using a negative binomial regression with robust standard errors and the observation time as a model offset. There were many patients without unplanned hospitalization days, leading to a 0 inflation for this outcome, and this was modeled using 0-inflated negative binomial regression with robust standard errors. The readmission costs for every patient who was readmitted were analyzed using linear regression with robust standard errors.

Due to the short follow-up time, dropout was rare and was assumed not to have been caused by readmittance or death. Dropout instances were censored for survival analyses, and the offset was adjusted accordingly for count outcomes. For patient satisfaction and readmission costs, we made multiple imputations for missing data based on baseline and outcome variables.

We repeated the above analyses for the per-protocol data set in our secondary analysis. We only considered patients with no missing values for the respective outcomes—those who had received the predischarge component plus at least 1 of the 2 postdischarge follow-up telephone calls (if in the intervention group), were discharged home, violated no eligibility criteria, and were stratified in the correct risk category.

The primary outcome was also analyzed in prespecified subgroups (simplified HOSPITAL score of 4–5 vs ≥6 points, clinical site, having diabetes, chronic heart failure, chronic obstructive pulmonary disease [COPD], or cancer, living in a nursing home, living alone, or having health insurance) using multivariable logistic regression with treatment and subgroup interaction terms to detect effect modifications. We also calculated stratum-specific estimates by risk of readmission and center (ie, including their interaction) because there was evidence that the intervention’s effect was not homogeneous over the strata (post hoc analysis).

We also performed a set of sensitivity analyses. First, we excluded early readmissions or deaths (within 1 day of discharge) from the analysis of the primary outcome. Second, we analyzed all outcomes without adjusting for the site and simplified HOSPITAL score. Third, we used survival methods to analyze the primary outcome by calculating the risk difference at 30 days from flexible parametric survival models. Fourth, we assumed a negative CTM-3 score result for patients who died. Fifth, readmission costs were analyzed using a generalized linear model with a γ distribution and a log link. Sixth, we excluded patients living in nursing homes from the analysis of readmission or death.

All of these analyses were predefined in a statistical analysis plan developed before study completion (Supplement 1) and were accompanied by 2-sided 95% CIs and a 2-sided *P* = .05. We used STATA software, version 15.1 (StataCorp LLC), with figures drawn using R software, version 3.6.2 (R Foundation for Statistical Computing).

## Results

Over 21 months, we enrolled and randomized 1386 participants for our primary analysis (Figure 1). The most frequent reason for study exclusion was declining to participate (n = 1012); 644 patients were excluded due to cognitive impairment or the inability to give consent. The randomization resulted in well-balanced groups (Table 1).

**Table 1.** Baseline Patient Characteristics

Characteristic	No. (%)^a^		
	Total (N = 1386)	Intervention group (n = 692)	Control group (n = 694)
Patient age, mean (SD), y	72 (14)	71 (14)	72 (14)
Sex			
Female	674 (49)	355 (51)	319 (46)
Male	712 (51)	337 (49)	395 (54)
Nationality			
Swiss	1182 (85)	587 (85)	595 (86)
German	15 (1.1)	11 (1.6)	4 (0.6)
French	27 (1.9)	13 (1.9)	14 (2.0)
Italian	65 (4.7)	29 (4.2)	36 (5.2)
Spanish/Portuguese	39 (2.8)	21 (3.0)	18 (2.6)
Eastern European	9 (0.6)	3 (0.4)	6 (0.9)
African	20 (1.4)	10 (1.4)	10 (1.4)
Other	29 (2.1)	18 (2.6)	11 (1.6)
Living status			
With spouse or partner	682 (49)	334 (48)	348 (50)
With another person	90 (6.5)	55 (7.9)	35 (5.0)
Alone	614 (44)	303 (44)	311 (45)
Living place			
Home	1206 (87)	590 (85)	616 (89)
Sheltered accommodation	25 (1.8)	11 (1.6)	14 (2.0)
Nursing home	145 (10)	82 (12)	63 (9.1)
Other	10 (0.7)	9 (1.3)	1 (0.1)
Home visits from a nurse	573 (46)	302 (50)	271 (43)
Home-care support			
For cleaning^b^	495 (40)	246 (40)	249 (39)
For buying groceries^b^	211 (17)	108 (18)	103 (16)
For feeding^b^	218 (18)	115 (19)	103 (16)
Index hospital admission			
Index hospitalization length of stay, median (IQR), d^b^	10 (7-14)	10 (7-14)	10 (8-13)
Type of admission			
Elective	102 (7.4)	43 (6.2)	59 (8.5)
Nonelective	1284 (93)	649 (94)	635 (91)
Main diagnostic category			
Heart failure	148 (11)	74 (11)	74 (11)
Acute ischemic heart disease	29 (2.1)	12 (1.7)	17 (2.4)
Arrhythmia	19 (1.4)	10 (1.4)	9 (1.3)
Venous thromboembolism	29 (2.1)	18 (2.6)	11 (1.6)
Stroke/TIA	25 (1.8)	10 (1.4)	15 (2.2)
COPD exacerbation	51 (3.7)	24 (3.5)	27 (3.9)
Pneumonia	113 (8.2)	53 (7.7)	60 (8.6)
Other infection, sepsis	163 (12)	79 (11)	84 (12)
Gastrointestinal disorder	142 (10)	78 (11)	64 (9.2)
Liver disorder	31 (2.2)	18 (2.6)	13 (1.9)
Kidney disorder	56 (4.0)	27 (3.9)	29 (4.2)
Nutritional or metabolic disorder	51 (3.7)	27 (3.9)	24 (3.5)
Adverse drug event	16 (1.2)	13 (1.9)	3 (0.4)
Neoplasm	198 (14)	92 (13)	106 (15)
Epilepsy	17 (1.2)	12 (1.7)	5 (0.7)
Other	298 (22)	145 (21)	153 (22)
No. of hospitalizations at the same hospital in the last 12 mo, median (IQR)	1.0 (0.0-2.0)	1.0 (0.0-2.0)	1.0 (0.0-2.0)
Simplified HOSPITAL score, median (IQR)^c^	5.0 (4.0-6.0)	5.0 (4.0-6.0)	5.0 (4.0- 6.0)
Destination after discharge^b^			
Home	1207 (87)	591 (85)	616 (89)
Nursing home	150 (11)	86 (12)	64 (9.2)
Other acute care hospital (including psychiatric)	2 (0.1)	1 (0.1)	1 (0.1)
Rehabilitation (general, cardiovascular, and neuro)	8 (0.6)	4 (0.6)	4 (0.6)
Palliative care	7 (0.5)	4 (0.6)	3 (0.4)
Other	11 (0.8)	6 (0.9)	5 (0.7)
Unknown	1 (0.1)	0 (0.0)	1 (0.1)
Patient self-discharged against medical advice	14 (1.0)	8 (1.2)	6 (0.9)
Comorbidity			
Chronic heart failure	372 (27)	194 (28)	178 (26)
Coronary disease	362 (26)	184 (27)	178 (26)
Atrial fibrillation	294 (21)	141 (20)	153 (22)
Peripheral artery disease	157 (11)	79 (11)	78 (11)
Diabetes	384 (28)	200 (29)	184 (27)
Dementia	32 (2.3)	14 (2.0)	18 (2.6)
Chronic obstructive pulmonary disease	223 (16)	105 (15)	118 (17)
Active cancer	594 (43)	288 (42)	306 (44)
Chronic kidney failure	399 (29)	205 (30)	194 (28)
Liver cirrhosis	99 (7.1)	46 (6.6)	53 (7.6)
Drug or alcohol abuse	197 (14)	100 (14)	97 (14)
Epilepsy	60 (4.3)	37 (5.3)	23 (3.3)
Any treated psychiatric disease	236 (17)	118 (17)	118 (17)

Abbreviations: COPD, chronic obstructive pulmonary disease; TIA, transient ischemic attack.

^a^ Percentage values are rounded to 2 digits (≥10 to whole numbers and <10 to a single decimal place).

^b^ Not applicable if living in a nursing home.

^c^ For the simplified HOSPITAL score, 0-4: readmission unlikely; 5 or more: readmission likely.

### Intervention

In the intervention group, 688 patients (99%) received basic information about their main chronic diseases (eTable 3 in Supplement 2), with 681 patients (98%) undergoing medication reconciliation and 235 patients (34%) having their medication discrepancies corrected. By the day-3 follow-up telephone call (n=661 patients reached by telephone), 356 patients (54%) had already visited their PCP at least once; by the day-14 telephone call (n=630 patients reached by telephone), 558 patients (89%) had visited at least once (eTable 4 in Supplement 2). Based on new or worsening symptoms, 183 patients (29%) were encouraged to consult their treating physician on day 3, and 206 patients (31%) were encouraged to do so on day 14.

### Unplanned Readmission or Death

The primary outcome was experienced by 145 patients (21%;95% CI, 18% to 24%) in the intervention group and 134 patients (19%; 95% CI, 17% to 22%; *P* = .44) in the control group (Table 2). Secondary analysis of the per-protocol data set confirmed the main analysis’s findings (eTable 5 in Supplement 2). The intention-to-treat analysis risk difference was 1.7% (95% CI, −2.5% to 5.9%; *P* = .44).

**Table 2.** Main Results at 30-Day Postdischarge From the Index Hospital Admission^a^

Main result	No. (%, 95% CI)		Difference or IRR (95% CI)	*P* value
	Intervention group (n = 692)	Control group (n = 694)		
Primary outcome				
Unplanned readmission or death	145 (21.0, 18.1 to 24.1)	134 (19.3, 16.5 to 22.4)	Risk difference: 1.7 (−2.5 to 5.9)^b^	.44
Secondary outcome				
Death	32 (4.6, 3.3 to 6.5)	18 (2.6, 1.6 to 4.1)	Risk difference: 2.0 (0.1 to 4.0)	.04
Death without unplanned readmission	18 (2.8, 1.8 to 4.3)	10 (1.5, 0.8 to 2.7)	Risk difference: 1.3 (−0.2 to 2.8)^b^	.10
Unplanned readmission	127 (19.3, 16.6 to 22.6)	124 (18.1, 15.4 to 21.1)	Risk difference: 1.3 (−2.9 to 5.4)^b^	.55
Time to unplanned readmission or death, RMST (95% CI), d	26.8 (26.3 to 27.3)	27.0 (26.5 to 27.6)	Difference: −0.2 (−0.9 to 0.5)^c^	.51
Health care use (incidence per 30 d, 95% CI)				
No. of unplanned hospitalization days	1118 (1.50, 1.42 to 1.59)	1191 (1.51, 1.42 to 1.60)	IRR: 0.81 (0.57 to 1.15)^d^	.24
No. of planned hospitalization days	136 (0.18, 0.15 to 0.22)	202 (0.26, 0.22 to 0.29)	IRR: 0.84 (0.39 to 1.81)^d^	.65
No. of unplanned hospital readmissions	135 (0.18, 0.15 to 0.21)	140 (0.18, 0.15 to 0.21)	IRR: 1.02 (0.81 to 1.30)^d^	.85
No. of planned hospital readmissions	28 (0.04, 0.03 to 0.05)	36 (0.05, 0.03 to 0.06)	IRR: 0.83 (0.50 to 1.37)^d^	.46
No. of emergency department visits	51 (0.07, 0.05 to 0.09)	55 (0.07, 0.05 to 0.09)	IRR: 0.94 (0.62 to 1.43)^c^	.76
No. of primary care provider consultations	1103 (1.52, 1.43 to 1.61)	1158 (1.51, 1.43 to 1.60)	IRR: 1.00 (0.92 to 1.09)^d^	.99
Patient satisfaction				
Satisfied with quality of care transition (3 items on the CTM-3)	575 (83.1, 79.9 to 86.3)	585 (84.3, 81.3 to 87.4)	Risk difference: −1.3 (−5.6 to 3.1)^b^	.52
Readmission cost, mean (SD), Swiss francs				
Readmission cost per patient (multiply imputed data)	15 355 (20 723)^e^	15 921 (24 391)^f^	Mean difference: −1075 (−5314 to 3165)^g^	.62

Abbreviations: CTM-3, 3-item Care Transitions Measure; IRR, incidence rate ratio; RMST, restricted mean survival time.

^a^ Binary outcomes are presented with risks and risk difference in days to primary outcome, with the RMST truncated at 30 days, and count outcomes with incidence (per 30 patient-days) and IRR.

^b^ A negative difference would indicate an intervention benefit.

^c^ The RMST was truncated at 30 days: a positive difference would indicate an intervention benefit.

^d^ An IRR smaller than 1 would indicate an intervention benefit.

^e^ N = 127.

^f^ N = 124.

^g^ A negative mean difference would indicate an intervention benefit.

**Figure 3.**
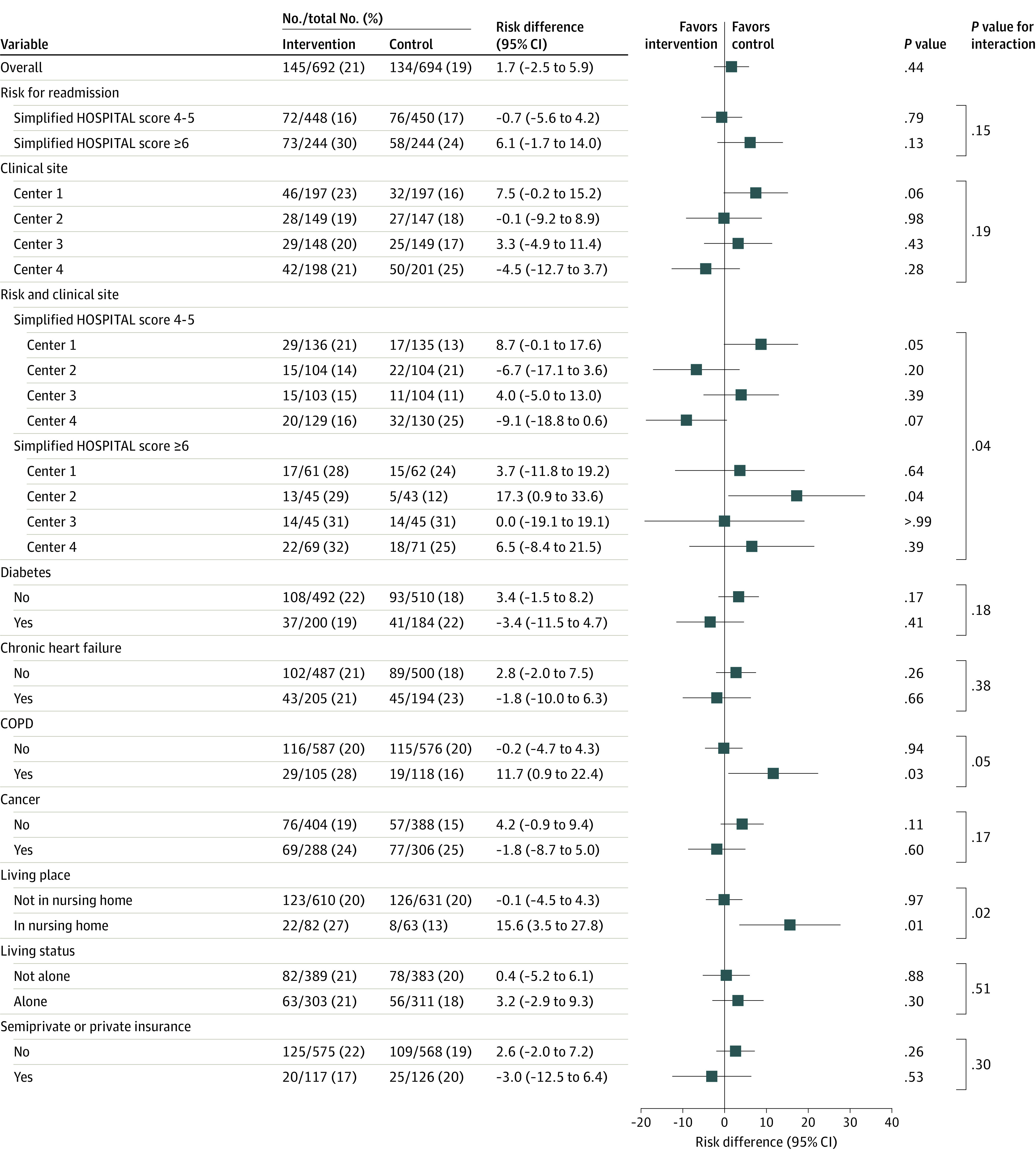
Subgroup Analysis for the Primary Outcome (Postdischarge 30-Day Unplanned Readmission or Death) The simplified HOSPITAL score ranges are 0-4 indicating readmission unlikely and 5 or more indicating readmission likely. COPD indicates chronic obstructive pulmonary disease.

The strata-specific analysis (Figure 3) (eTable 6 and eTable 7 in Supplement 2) showed heterogeneity, the intervention having negative effects on one of the center’s patients at very-high risk (simplified HOSPITAL score ≥6) and a tendency toward benefits for another center’s patients with simplified HOSPITAL scores of 4 to 5. Little evidence was found for any effect in other strata. Interactions between the intervention and risk groups or between centers were not statistically significant (Figure 3). The intervention increased the risks of a primary outcome among patients with COPD or living in nursing homes, with evidence of effect modification in both cases.

### Secondary Outcomes

There were 251 (18%) unplanned readmissions overall. The main diagnosis categories at readmission were infection (24%), oncology (21%), and heart failure (10%) (eTable 8 in Supplement 2). Diagnoses were the same as index admissions for 76 patients (33%). There was no difference between treatment groups in the risk of unplanned readmissions without death (Table 2), but strata-specific analyses showed the same heterogeneity as for the primary outcome (eTables 6-7 in Supplement 2). Thirty-two (4.6%) intervention group patients died, as did 18 (2.6%; *P* = .04) in the control group. The difference in death without unplanned readmission was not statistically significant. We found no differences in health care use, patient satisfaction, or readmission costs in the intervention group (Table 2).

### Sensitivity Analysis

Sensitivity analyses confirmed the findings of the main analyses (eTables 9-14 in Supplement 2). The difference in mortality between groups was no longer present when patients who lived in nursing homes were excluded.

## Discussion

The present multicenter RCT targeted patients at a higher risk of hospital readmission using a standardized care transition intervention composed of predischarge patient education and 2 postdischarge follow-up telephone calls. It did not decrease the risk of postdischarge unplanned readmission or death. Similarly, we found no effects on postdischarge health care use, patient satisfaction with the quality of care transitions, or readmission costs. Indeed, we found a small but significant increase in the risk of death in the intervention group.

Hospital readmissions have received great deal of attention recently, especially since the 2010 Affordable Care Act established the US Hospital Readmission Reduction Program. Although numerous heterogeneous studies have tried to reduce readmission risks, few interventions have shown clear, positive reproducible effects,^14^ especially among patients with medical multimorbidity. To our knowledge, this multicenter RCT was one of the first multimodal interventions targeting patients at higher risk, defined using a validated score, in a medical patient population.

The lack of a significant impact on unplanned readmission could have several explanations. First, the intervention may have identified new or deteriorating symptoms earlier than in the usual care group. Approximately 30% of the intervention group was asked to contact their PCP. A proportion of these follow-up visits could have led to hospitalization. Indeed, some authors advocate against focusing on readmission alone when trying to appreciate the benefits of transitional care interventions,^18,19^ especially because some readmissions are medically necessary and the readmission outcome is an insensitive measure of safety and patient centeredness. Second, the intervention group’s proportion of patients living in nursing homes was slightly higher. The intervention performed worse than usual care for this subgroup of patients. Third, although all members of the study team underwent standardized training, many of the multimodal intervention’s components were dependent on individual skills. Similarly, the usual care received by the control group’s patients may have differed by hospital (eg, baseline rates of scheduling follow-up visits with PCPs) and could partially explain center-related differences in the intervention’s benefits. Finally, we cannot exclude that the intervention was not sufficiently comprehensive, as it did not include family and community support and was limited to symptom monitoring and management. Family involvement may be particularly important due to lack of patient retention of information on the day of discharge, as evidenced by the lack of improvement in CTM-3 scores in the intervention group. On the other hand, a more complex intervention would have faced the difficulties of resource scarcity.

Considering the nature of the intervention, the increase in mortality is difficult to explain. Perhaps the higher number of patients living in nursing homes in the intervention group resulted in an incomplete adjustment for confounding. Because most of these deaths ended the patient’s period of readmission, a direct causal effect of the intervention seems less likely. The intervention was not associated with any statistical increase in deaths when considering death without an unplanned readmission or when excluding patients living in nursing homes.

Several systematic reviews and meta-analyses have reported that interventions are effective at reducing overall readmissions.^14,20,21,22,23,24^ However, most of these intervention studies were performed on specific patient populations or only targeted older patients. Age itself is not a risk factor for readmission.^25,26^ Few studies targeted patients at a high risk of readmission. Schnipper et al^18^ used a multifaceted intervention, similar to ours. However, these were not patients at high risk of readmission (median simplified HOSPITAL score, 3). Although the study found that the intervention reduced postdischarge adverse events, no difference in the risk of unplanned readmissions was observed. Another large interventional study^27^ of US veterans compared a multimodal intervention with a propensity-matched control cohort, finding an increase in medical consultations and a reduction in 30-day deaths but no benefits on the risk of readmission or emergency department visits. Finally, in an RCT, Dhalla et al^28^ used the LACE index (risk scoring criteria based on length of stay, acuity of the admission, comorbidities, and emergency department use 6 months before admission) to target patients at a high risk of readmission, but they found that their multimodal intervention had no benefits for the risks of readmission or death. Thus, standardized care transition interventions for general medical patients may display some benefits, such as reinforced medical management for outpatients or reduced adverse medical events,^18^ but with no effects on hospital readmission rates.

### Limitations

This study had a few limitations. First, outcome horizons were limited to 30 days. Although this is a common timeframe in this area of research, the intervention benefits may appear later. Second, due to the intervention design, study participants could not be blinded, which might have led to different health behaviors. Third, several components of the intervention were not fully standardized. Fourth, the study sample calculations assumed interhospital homogeneity, and thus, because of the observed heterogeneity, the study may have been underpowered. We did not collect medication reconciliation or PCP follow-up within 7 days in the usual care arm to evaluate for the mediating effects of these factors. Fifth, the intervention effects on patients with cognitive impairment remain uncertain as such patients were excluded from the study. Finally, as a multimodal intervention, the design did not allow us to evaluate which components worked or did not work; this could have been better elucidated using factorial randomization.

## Conclusions

In this RCT, our multimodal intervention for medical inpatients at a higher risk of hospital readmission, defined using their simplified HOSPITAL score, did not show any significant benefits regarding reducing hospital readmissions. This particular intervention added to uncertainties about the real benefits and impacts of trying to monitor and reduce this indicator of care.

## Notes

Supplement 1. Trial Protocol and Statistical Analysis Plan

Supplement 2. **eTable 1.** Simplified HOSPITAL score Switzerland. **eTable 2.** Summary of Discharge Procedures for the Usual Care Groups **eTable 3.** Pre-Discharge Component of the Intervention **eTable 4.** Follow-Up Phone Call **eTable 5.** Secondary Analysis (Per-Protocol) of Main Outcomes at 30-Days **eTable 6.** Strata-Specific Primary Analysis (Intent-to-Treat) for the Primary Outcome **eTable 7.** Strata-Specific Secondary Analysis (Per Protocol) for the Primary Outcome **eTable 8.** Main Diagnoses of Unplanned Readmissions **eTable 9.** Sensitivity Analyses; Primary Outcome Excluding Early Readmissions or Deaths **eTable 10.** Sensitivity Analyses; All Outcomes Without Adjusting for Stratification Factors **eTable 11.** Sensitivity Analyses; Primary Outcome Using Survival Methods by Calculating the Risk Difference at 30 Days From Flexible Parametric Survival Models **eTable 12.** Sensitivity Analyses; Assuming a Negative CTM-3 Score Result for Patients Who Died **eTable 13.** Sensitivity Analyses for Readmission Costs Using the Mean Ratio Calculated From a Gamma Regression **eTable 14.** Sensitivity Analysis for Unplanned Readmission and Death, Excluding Patients Living in Nursing Homes

Supplement 3.
Data Sharing Statement
