# Supplementary material for: Effects of a Multimodal Transitional Care Intervention in Patients at High Risk of Readmission: The TARGET-READ Randomized Clinical Trial
Source: JAMA Intern Med. 2023 May 1;183(7):658–68. doi: 10.1001/jamainternmed.2023.0791 (PMC10152373; doi:10.1001/jamainternmed.2023.0791)
Supplement: Supplement 1. — Trial Protocol and Statistical Analysis Plan [file jamainternmed-e230791-s001.pdf]

Change history

Version Nr	Version date	Modified without version change	Description, comments	Control
1.0	28.12.17		Initial version	JD
1.1	13.02.18		1st revision based on KEK Bedingungen	JD
1.2	15.05.18		2nd revision with clarifications	JD

Research Plan

Transition cAre inteRvention tarGeted to high-risk patiEnts To Reduce rEADmission (TARGET-READ):

A randomized controlled trial

Short Title: Target-READ Phase 2

Study Type:	Clinical trial
Study Categorisation:	Risk category A: Clinical trials with interventions that are neither a therapeutic product nor a transplant product, nor a transplant
Study Registration:	SNCTP Portal SNCTP000002799.<br>ClinicalTrials.gov: NCT03496896.
Study Identifier:	ID 2018-00084
Sponsor, Sponsor-Investigator or Principal Investigator:	Prof. Dr. med. Jacques Donzé, MSc<br>Leitender Arzt<br>Universitätsklinik für Allgemeine Innere Medizin<br>Haus 5, H22<br>INSELSPITAL, Universitätspital Bern<br>3010 Bern<br>Switzerland<br>Telefon: +41 (31) 632.57.69
Investigational Product:	interventions that are neither a therapeutic product nor a transplant product, nor a transplant
Protocol Version and Date:	Version 1.2 of 15.05.2018

CONFIDENTIAL

e.g. “The information contained in this document is confidential and the property of Prof. Dr. med. Jacques Donzé (or “sponsor”). The information may not - in full or in part - be transmitted, reproduced, published, or disclosed to others than the applicable Competent Ethics Committee(s) and Regulatory Authority(ies) without prior written authorisation from the sponsor except to the extent necessary to obtain informed consent from those who will participate in the study.

Signature Page

Study number	ID 2018-00084
Study Title	Transition cAre inteRvention tarGeted to high-risk patiEnts To Reduce rEADmission (TARGET-READ): A randomized controlled trial

The Sponsor-Investigator and trial statistician have approved the protocol version 1.2 dated 15.05.2017, and confirm hereby to conduct the study according to the protocol, current version of the World Medical Association Declaration of Helsinki, ICH-GCP guidelines or ISO 14155 norm if applicable and the local legally applicable requirements.

Sponsor:

Prof. Jacques Donzé

Place/Date	Signature

Local Principal Investigator at study site*:

I have read and understood this trial protocol and agree to conduct the trial as set out in this study protocol, the current version of the World Medical Association Declaration of Helsinki, ICH-GCP guidelines or ISO 14155 norm and the local legally applicable requirements.

Site	CHUV Lausanne
Principal investigator	Dr Marco Mancinetti

Place/Date	Signature

**Note:* In multicentre studies, this page must be individually signed by all participating Local Principal Investigators.

Table of Contents

SHORT TITLE: TARGET-READ PHASE 2 ... 2
STUDY SYNOPSIS ... 8
STUDY SUMMARY IN LOCAL LANGUAGE ... 11
ABBREVIATIONS ... 12
STUDY SCHEDULE ... 13
1. STUDY ADMINISTRATIVE STRUCTURE ... 14
1.1 Sponsor, Sponsor-Investigator ... 14
1.2 Principal Investigator(s) ... 14
1.3 Statistician ("Biostatistician") ... 15
1.4 Laboratory ... 15
1.5 Monitoring institution ... 15
1.6 Data Safety Monitoring Committee ... 15
1.7 Any other relevant Committee, Person, Organisation, Institution ... 15
2. ETHICAL AND REGULATORY ASPECTS ... 16
2.1 Study registration ... 16
2.2 Categorisation of study ... 16
2.3 Competent Ethics Committee (CEC) ... 16
2.4 Competent Authorities (CA) ... 16
2.5 Ethical Conduct of the Study ... 16
2.6 Declaration of interest ... 16
2.7 Patient Information and Informed Consent ... 16
2.8 Participant privacy and confidentiality ... 17
2.9 Early termination of the study ... 17
2.10 Protocol amendments ... 17
3. BACKGROUND AND RATIONALE ... 18
3.1 Background and Rationale ... 18
3.2 Investigational Product (treatment, device) and Indication ... 21
3. Medications: **... 22**
4. Patient education: **... 22**
3.3 Preclinical Evidence ... 22
3.4 Clinical Evidence to Date ... 22
3.5 Dose Rationale / Medical Device: Rationale for the intended purpose in study (pre-market MD) 22
3.6 Explanation for choice of comparator (or placebo) ... 22
3.7 Risks / Benefits ... 23
3.8 Justification of choice of study population ... 24
4. STUDY OBJECTIVES ... 25
4.1 Overall Objective ... 25
4.2 Primary Objective ... 25
4.3 Secondary Objectives ... 25
4.4 Safety Objectives ... 25
5. STUDY OUTCOMES ... 26
5.1 Primary Outcome ... 26
5.2 Secondary Outcomes ... 26
5.3 Other Outcomes of Interest ... 27

5.4 Safety Outcomes ... 27
6. STUDY DESIGN ... 28
6.1 General study design and justification of design ... 28
6.2 Methods of minimising bias ... 29
6.2.1 Randomisation ... 29
6.2.2 Blinding procedures ... 29
6.2.3 Other methods of minimising bias ... 29
6.3 Unblinding Procedures (Code break) ... 29
7. STUDY POPULATION ... 30
7.1 Eligibility criteria ... 30
7.2 Recruitment and screening ... 31
7.3 Assignment to study groups ... 32
7.4 Criteria for withdrawal / discontinuation of participants ... 32
8. STUDY INTERVENTION ... 33
8.1 Identity of Investigational Products (treatment / medical device) ... 33
8.1.1 Experimental Intervention (treatment / medical device) ... 34
8.1.2 Control Intervention (standard/routine/comparator treatment / medical device) ... 34
8.1.3 Packaging, Labelling and Supply (re-supply) ... 34
8.1.4 Storage Conditions ... 34
8.2 Administration of experimental and control interventions ... 34
8.2.1 Experimental Intervention ... 34
8.2.2 Control Intervention ... 34
8.3 Dose / Device modifications ... 34
8.4 Compliance with study intervention ... 34
8.5 Data Collection and Follow-up for withdrawn participants ... 35
8.6 Trial specific preventive measures ... 35
8.7 Concomitant Interventions (treatments) ... 35
8.8 Study Drug / Medical Device Accountability ... 35
8.9 Return or Destruction of Study Drug / Medical Device ... 35
9. STUDY ASSESSMENTS ... 36
9.1 Study flow chart(s) / table of study procedures and assessments ... 36
9.2 Assessments of outcomes ... 37
9.2.1 Assessment of primary outcome ... 37
9.2.2 Assessment of secondary outcomes ... 37
9.2.3 Assessment of other outcomes of interest ... 38
9.2.4 Assessment of safety outcomes ... 38
9.2.5 Assessments in participants who prematurely stop the study ... 38
9.3 Procedures at each visit ... 38
9.3.1 Screening (Visit 0) ... 39
9.3.2 Visit 1: Patient inclusion and pre-discharge intervention ... 39
9.3.3 Visit 2: Post-discharge follow-up phone call 1 (Day 3 ±1) ... 39
9.3.4 Visit 3: Post-discharge follow-up phone call 2 (Day 14 ±1) ... 39
9.3.5 Visit 4: Outcome collection (Day 30 up to day 45) ... 39
10. SAFETY ... 41
10.1 Drug studies ... 41
10.2 Medical Device Category C studies ... 41

10.3 Medical Device Category A studies ... 41
10.4 Other clinical studies ... 41
10.4.1 Definition and assessment of serious adverse events and other safety related events ... 41
10.4.2 Reporting of serious adverse events (SAE) and other safety related events ... 42
10.4.3 Follow up of (Serious) Adverse Events ... 42
11. STATISTICAL METHODS ... 43
11.1 Hypothesis ... 43
11.2 Determination of Sample Size ... 43
11.3 Statistical criteria of termination of trial ... 43
11.4 Planned Analyses ... 43
11.4.1 Datasets to be analysed, analysis populations ... 43
11.4.2 Primary Analysis ... 43
11.4.3 Secondary Analyses ... 44
11.4.4 Interim analyses ... 44
11.4.5 Safety analysis ... 44
11.4.6 Deviation(s) from the original statistical plan ... 44
11.5 Handling of missing data and drop-outs ... 44
12. QUALITY ASSURANCE AND CONTROL ... 45
12.1 Data handling and record keeping / archiving ... 45
12.1.1 Case Report Forms ... 45
12.1.2 Specification of source documents ... 45
12.1.3 Record keeping / archiving ... 45
12.2 Data management ... 45
12.2.1 Data Management System ... 46
12.2.2 Data security, access and back-up ... 46
12.2.3 Analysis and archiving ... 46
12.2.4 Electronic and central data validation ... 46
12.3 Monitoring ... 47
12.4 Audits and Inspections ... 47
12.5 Confidentiality, Data Protection ... 47
12.6 Storage of biological material and related health data ... 48
13. PUBLICATION AND DISSEMINATION POLICY ... 49
14. FUNDING AND SUPPORT ... 50
14.1 Funding ... 50
14.2 Other Support ... 50
15. INSURANCE ... 50
16. REFERENCES ... 51
17. APPENDICES ... 54

Study synopsis

**Sponsor / Sponsor-Investigator**	Jacques Donzé<br>Universtitätsklinik für Allgemeine Innere Medizin<br>Inselspital, 3010 Bern<br>Email: jacques.donze@insel.ch<br>Phone: 031/632.57.69<br>Fax: 031/632.42.58
**Study Title:**	Transition cAre inteRvention tarGeted to high-risk patiEnts To Reduce rEADmission (TARGET-READ): A randomized controlled trial
**Short Title / Study ID:**	Target-READ Phase 2
**Protocol Version and Date:**	Version 1.2, 15.05.2018
**Trial registration:**	SNCTPregistration: SNCTP000002799.<br>ClinicalTrials.gov Identifier: NCT03496896.
**Study category and Rationale**	Clinical trial.<br>Risk category A: Clinical trials with interventions that are neither a therapeutic product nor a transplant product, nor a transplant.<br>The investigator randomly assigned ("prospectively assigned") patients discharged home from the hospital to receive either standard intervention to reduce the risk of hospital readmission. The intervention include patient education, medication reconciliation, follow-up appointment planning, follow-up phone calls. A teaching and follow-up call intervention is not a medicinal product or device, a transplant or transplant product, a gene therapy, or a pathogenic organism.
**Clinical Phase:**	NA
**Background and Rationale:**	Hospital readmissions within 30 days are frequent. They represent a burden for the patients, but also for the entire health care system. Is it therefore recognized as important to improve the quality of the transition of care period in order to avoid as much as possible potentially avoidable readmissions. There are however still several gaps in current knowledge. First, most trials to reduce hospital readmission have been performed on specific patient populations such as patients with diabetes or heart failure, but less to an overall population of medical patients. Second, the most effective transition care interventions were also the most complex ones, but trials failed to target the medical patients who are most likely to benefit for better effectiveness. To identify those high-risk patients, no trials used one of the best validated, accurate, and easy-to-use tools, such as the “HOSPITAL” score. Finally, No interventional studies to reduce readmission have been previously performed in general medical patients in Switzerland.
**Objective(s):**	To evaluate in a randomized controlled trial the effect of a transitional care intervention prioritized to higher-risk medical patients (using the HOSPITAL score) on the composite of 30-day unplanned readmissions and death.
**Outcome(s):**	Primary outcome: unplanned readmission or death within 1 month after hospital discharge.<br>Secondary outcomes: time to readmission/death, main cause of readmission, post-discharge care utilization, medication adherence, patient’s perspective on quality of transition of care.
**Study design:**	Single-blinded randomized controlled trial.
**Inclusion / Exclusion criteria:**	All adult patients discharged home or nursing home after a hospital stay of one day or more in the department of medicine of the 4 participating hospitals will be screened.<br>Patients already enrolled in the current study, not living in Switzerland, not speaking French/German, not having a phone number to be reached at, , or with cognitive troubles will be excluded.<br>Only patients with a high risk of 30-day readmission estimated with the simplified “HOSPITAL” score will be invited to participate.

**Measurements and procedures:**	All patients will have their risk of readmission estimated according to the simplified “HOSPITAL” score, and have demographics, lab values, and diagnoses captured at inclusion.<br>The patients randomized to the intervention group will receive a pre-discharge intervention with patient education, medication reconciliation, and follow-up appointment organization. They will then receive 2 short follow-up phone calls at 3 and 14 days after discharge to ensure medication adherence and follow-up by the primary care physician.<br>Outcomes will be collected at 1 month by phone call interview by a blinded study nurse to capture outcomes.
**Study Product / Intervention:**	The intervention doesn’t involve any medical drug or device, but: 1) Patient education on the specific diseases of the patients, including information leaflets about the most frequent diseases; 2) Medication reconciliation: the study nurse will review the list of medication with the patient and seek for any missing medication in the list; 3) Organization of a follow-up appointment by the primary care physician within 7 days after discharge; 4) Insure that a discharge summary is sent quickly to the primary care physician. 5) Two follow-up phone calls at day 3 and 14 after discharge to ensure medication and appointment adherence.
**Control Intervention (if applicable):**	The group control will receive usual care without additional intervention. Only an information sheet about the study participation will be given to the participant of the group control.
**Number of Participants with Rationale:**	We plan to include around 700 patients in each arm (total of 1,400 patients).
**Study Duration:**	Start of screening: March 2018<br>Recruitment phase: 20 months<br>Length of follow-up: 30 days<br>Study duration: 21 months
**Study Schedule:**	Month Year of First-Participant-In (planned): March, 2018.<br>Month Year of Last-Participant-Out (planned): August, 2019

**Investigator(s):**	Name: Dr. Marco Mancinetti<br>Institution: Hôpital cantonal de Fribourg<br>Address: Hôpital Fribourgeois, 1708 Fribourg<br>Email: Marco.Mancinetti@h-fr.ch<br>Phone: 026/306.23.60<br><br>Name: Prof. Daniel Genné<br>Institution: Centre Hospitalier Bienne<br>Address: Centre hospitalier Bienne, Bienne<br>Email: Daniel.Genne@szb-chb.ch<br>Phone: 032/324.34.38<br><br>Name: Dr. Marie Méan<br>Institution: CHUV<br>Address: CHUV, Rue du Bugnon, 1011 Lausanne<br>Email: Marie.Mean@chuv.ch<br>Phone: 079/556.69.17<br><br>Name: Dr Gregor John<br>Institution: Hôpital neuchâtelois (Hne), Pourtalès<br>Address: Rue de la Maladière 45, 2000 Neuchâtel<br>Email: gregor.john @h-ne.ch<br>Phone: 079/559.52.92
**Study Centre(s):**	National multicenter study including 4 participating hospital in Switzerland: Centre hospitalier Bienne, Hôpital fribourgeois, CHUV, and Hôpital neuchâtelois.
**Statistical Considerations:**	The primary outcome will be reported as a proportion of patients in each group and compared between the groups by a risk difference with 95% CI and a chi squared test. The primary analysis will be a comparison between two groups according to the intention-to-treat principle, i.e. all randomized patients will be analyzed in the allocated group regardless of any protocol violations such as cross-overs or early discontinuations of the intervention.<br>Because we will target patients at higher risk of readmission, we hypothesize that the intervention could reduce readmission by 25%. The expected 30-day readmission and death rate for patients at intermediate or high risk according to the simplified HOSPITAL score is around 27%. Allowing for 10% loss to follow-up, we determine that we will need 1,380 patients for the study to have 80% power, i.e. at least 690 in each arm.
**GCP Statement:**	This study will be conducted in compliance with the protocol, the current version of the Declaration of Helsinki, the ICH-GCP or ISO EN 14155 (as far as applicable) as well as all national legal and regulatory requirements.

Study summary in local language

Il est fréquent que des patients qui sortent de l'hôpital doivent y retourner dans les 30 jours. Ceci représente un problème de santé publique avec des conséquences lourdes pour les patients eux-mêmes et pour tout le système de soin. Il est donc important d'améliorer la qualité de la prise en charge entre hôpital et soins ambulatoires afin d'essayer de limiter cette problématique. Le projet vise à mieux comprendre les réadmissions en Suisse, et de tester un meilleur accompagnement au moment et après la sortie de l'hôpital chez les patients à haut risque d'être réhospitalisé.

L'étude TARGET-READ a pour but de tester l'effet d'une intervention pour améliorer la transition entre l'hôpital et les soins ambulatoires chez des patients à haut risque d'être réhospitalisés, en comparant ces patients avec un groupe de control. En tout 1400 patients seront inclus dans 4 hôpitaux suisses, et réparti aléatoirement soit dans le groupe qui reçoit l'intervention, soit dans le groupe contrôle.

Lors de l'étude, les patients à haut risque qui sortent de 4 hôpitaux suisse vont se voir offrir la possibilité de participer à l'étude. Les participants seront ensuite aléatoirement réparti soit dans le groupe avec intervention, soit dans le groupe contrôle. Le groupe avec intervention recevra la visite et un suivi téléphonique d'une infirmière de coordination avant la sortie de l'hôpital, afin d'organiser au mieux le suivi des patients dès leur sortie de l'hôpital. Cette étude permettra d'établir si une telle intervention est à même de réduire le risque de complications et de réadmission à 30 jours.

Ce projet de recherche clinique multicentrique a pour but d'évaluer si le risque de réhospitalisation chez des patients à haut risque peut être réduit. Alors que la pression s'accentue pour réduire la durée d'hospitalisation, cette étude s'inscrit dans un but d'amélioration des soins dans ce moment charnière qui est la transition entre l'hôpital et le domicile, afin de réduire les complications et un retour trop précoce à l'hôpital. Tous les acteurs de la santé peuvent potentiellement bénéficier des résultats de cette étude, des patients jusqu'aux hôpitaux bien sûr, en passant par tout le système de santé.

Abbreviations

Provide a list of abbreviations used on the protocol - to be completed

AE	Adverse Event
CA	Competent Authority (e.g. Swissmedic)
CEC	Competent Ethics Committee
CRF	Case Report Form
ClinO	Ordinance on Clinical Trials in Human Research *(in German: KlinV, in French: OClin)*
eCRF	Electronic Case Report Form
CTCAE	Common terminology criteria for adverse events
DSUR	Development safety update report
GCP	Good Clinical Practice
IB	Investigator's Brochure
Ho	Null hypothesis
H1	Alternative hypothesis
HFG	Humanforschungsgesetz (Law on human research)
HMG	Heilmittelgesetz
HRA	Federal Act on Research involving Human Beings
IMP	Investigational Medicinal Product
IIT	Investigator-initiated Trial
ISO	International Organisation for Standardisation
ITT	Intention to treat
KlinV	Verordnung über klinische Versuche in der Humanforschung *(in English: ClinO, in French OClin)*
LPTh	Loi sur les produits thérapeutiques
LRH	Loi fédérale relative à la recherche sur l'être humain
MD	Medical Device
OClin	Ordonnance sur les essais cliniques dans le cadre de la recherche sur l'être humain *(in German : KlinV, in English : ClinO)*
PI	Principal Investigator
SDV	Source Data Verification
SOP	Standard Operating Procedure
SPC	Summary of product characteristics
SUSAR	Suspected Unexpected Serious Adverse Reaction
TMF	Trial Master File

Study schedule

Overview Data Collection

Target-READ Trial — FUNDED BY THE SWISS NATIONAL SCIENCE FOUNDATION

	PHASE II	
	**Index Discharge**	**Outcomes 30 days**
Eligibility criteria	x	
HOSPITAL score	x	
Contact info	x	
Baseline characteristics	x	
Index diagnoses	x	
Outcomes 30 days		x
Late documents (discharge letter)		x
End of study		x

1. STUDY ADMINISTRATIVE STRUCTURE

1.1 Sponsor, Sponsor-Investigator

Sponsor, Project Leader and Coordinating researcher (identical)	Name: Jacques Donzé<br>Address: Universtitätsklinik für Allgemeine Innere Medizin, Inselspital, 3010 Bern<br>Email: Jacques.donze@insel.ch<br>Phone: 031/632.57.69<br>Fax: 031/632.42.58

The sponsor designed the study, obtained the research funding, and wrote the protocol. He will supervise the data collection, manage the study nurses at each site, supervise the analysis done by the Clinical Trial Unit of the Bern University, interprete the study results with the help of the biostatistician, and write the report and final manuscript.

1.2 Principal Investigator(s)

Project site(s) and responsible researcher:	Institution: Hôpital cantonal de Fribourg<br>Name: Dr. Marco Mancinetti<br>Address: Hôpital Fribourgeois, 1708 Fribourg<br>Email: Marco.Mancinetti@h-fr.ch<br>Phone: 026/306.23.60<br><br>Institution: Centre Hospitalier Bienne<br>Name: Prof. Daniel Genné<br>Address: Centre hospitalier Bienne, Bienne<br>Email: Daniel.Genne@szb-chb.ch<br>Phone: 032/324.34.38<br><br>Institution: CHUV<br>Name: Dr Marie Méan<br>Address: CHUV, Rue du Bugnon, 1011 Lausanne<br>Email: Marie.Mean@chuv.ch<br>Phone: 079/556.69.17<br><br>Institution: Hôpital neuchâtelois (Hne), Pourtalès<br>Name: Dr Gregor John<br>Address: Rue de la Maladière 45, 2000 Neuchâtel<br>Email: gregor.john @h-ne.ch<br>Phone: 079/559.52.92

The local investigator will help the Sponsor to implement the study at each site, and give local logistical

support.

1.3 Statistician ("Biostatistician")

Biostatistician:	Name: Lukas Bütikofer<br>Address: CTU Bern, University of Bern, Mittelstrasse 43, 3012 Bern<br>Email: lukas.buetikofer@ctu.unibe.ch<br>Phone: 031/631.35.10

1.4 Laboratory

Not applicable

1.5 Monitoring institution

Monitoring institution:	Name: PD Dr. Sven Trelle<br>Address: CTU Bern, University of Bern, Mittelstrasse 43, 3012 Bern<br>Email: sven.trelle@ctu.unibe.ch<br>Phone: 031/631.35.04

1.6 Data Safety Monitoring Committee

Due to the nature of the intervention, a DSMC is not needed. No change in the therapy will be made. The intervention doesn't included any medicinal product or device, transplant or transplant product, gene therapy, nor pathogenic organism.

1.7 Any other relevant Committee, Person, Organisation, Institution

Not applicable.

2. ETHICAL AND REGULATORY ASPECTS

The decision of the CEC concerning the conduct of the study will be made in writing to the Sponsor before commencement of this study. The clinical study can only begin once approval from all required authorities has been received. Any additional requirements imposed by the authorities shall be implemented.

2.1 Study registration

The study will be registered under the SNCTP Portal after the ethical committee approval (BASEC). We will also register the study under Clinicaltrials.gov before the inclusion of the first patients.

2.2 Categorisation of study

The study is a clinical trial. The study involve living persons over a certain period of time, to investigate the effects of a health-related intervention. To investigate the effects, the principal investigator exposes individuals who have been selected specifically for the trial to the intervention to be investigated, and then measures its effects on health.

The risk category is A: the clinical trial intervention include patient education, medication reconciliation, follow-up appointment planning, follow-up phone calls. This intervention is not a medicinal product or device, a transplant or transplant product, a gene therapy, or a pathogenic organism.

2.3 Competent Ethics Committee (CEC)

The project plan, the proposed participant information and consent form as well as other project-specific documents will be submitted to a competent Ethics Committee (CEC). No changes will be made to the protocol without prior Sponsor and CEC approval, except where necessary to eliminate apparent immediate hazards to study participants.

Premature study end or interruption of the study will be reported within 15 days. The regular end of the study will be reported to the CEC within 90 days. The project does not involve investigation with radioactive sources. The final study report will be submitted within one year after study end.

2.4 Competent Authorities (CA)

Not applicable.

2.5 Ethical Conduct of the Study

The study will be carried out in accordance to the protocol and with principles enunciated in the current version of the Declaration of Helsinki, the guidelines of Good Clinical Practice (GCP) issued by ICH, the Swiss Law and Swiss regulatory authority's requirements. The CEC will receive annual safety and interim reports and be informed about study stop/end in agreement with local requirements.

2.6 Declaration of interest

The sponsor has no existing conflict of interest.

2.7 Patient Information and Informed Consent

The investigators, or one of his/her delegated research team member will explain to each potential

participant the nature of the study, its purpose, the procedures involved, the expected duration, the potential risks and benefits and any discomfort it may entail. Each subject will be informed that the participation in the study is voluntary and that he/she may withdraw from the study at any time and that withdrawal of consent will not affect his/her subsequent medical assistance and treatment.

The subject will be informed that his/her medical records may be examined by authorised individuals other than their treating physician.

All potential study participants will be provided with a patient information sheet and a consent form describing the study and providing sufficient information and time for the subject to make an informed decision about their participation in the study.

The patient information sheet and the consent form will be submitted to the CEC to be reviewed and approved. The formal consent of a subject, using the approved consent form, will be obtained before the subject is submitted to any study procedure.

The subject should read and consider the statement before signing and dating the informed consent form, and should be given a copy of the signed document. The consent form will also be signed and dated by the investigator (or his designee, e.g. a study nurse or coordinator) and it will be retained as part of the study records.

2.8 Participant privacy and confidentiality

The investigator affirms and upholds the principle of the participant's right to privacy and that they shall comply with applicable privacy laws. Especially, anonymity of the participants shall be guaranteed when presenting the data at scientific meetings or publishing them in scientific journals.

Individual subject medical information obtained as a result of this study is considered confidential and disclosure to third parties is prohibited. Subject confidentiality will be further ensured by utilising subject identification code numbers to correspond to treatment data in the computer files.

For data verification purposes, authorised representatives of the Sponsor (-Investigator), a competent authority (e.g. Swissmedic), or an ethics committee may require direct access to parts of the medical records relevant to the study, including participants’ medical history.

2.9 Early termination of the study

Due to the intervention type of the study and the minimal risk for the patients, we are not foreseeing a possible early termination of the project. However in such case, the premature end or interruption of the research project will be reported to the CEC within 15 days.

2.10 Protocol amendments

Substantial amendments are only implemented after approval of the CEC.

Under emergency circumstances, deviations from the protocol to protect the rights, safety and well-being of human subjects may proceed without prior approval of the sponsor and the CEC. Such deviations shall be documented and reported to the sponsor and the CEC as soon as possible.

All non-substantial amendments are communicated to the CEC within the Annual Safety Report (ASR).

3. BACKGROUND AND RATIONALE

3.1 Background and Rationale

Importance of readmission

Each year, more than 1.4 million acute adult hospitalizations arise in Switzerland, among which about 12-15% are 30-day readmissions.[1] Transitions of care are indeed high-risk periods for patients, especially for those with multiple chronic diseases.[2] Many complications can occur as patients are discharged from hospitals to the ambulatory setting, which lead to unnecessary distress for the patients and costly readmissions.[3, 4] Each hospital admission is moreover associated with a risk of new complications.[5, 6] For these reasons, readmissions have been lately of high interest in health policies not only in the United States with the “Patient Protection and Affordable Care Act”,[7] but also in Switzerland with the promotion of coordination of care between the different settings being one of the aim of the comprehensive strategy entitled "Gesundheit2020" (Health 2020), a total of 36 measures across all areas of the health system.[8] Some financial incentives have been also put in places in order to promote a reduction in hospital readmission in Switzerland with the new SwissDRG system payment.

Preventability of readmission and interventions

It is estimated that 30% of the readmissions are preventable,[9] and about 50% potentially preventable.[10] In order to avoid these preventable readmissions, several transitional care interventions have shown promising results.[11-14] It includes telephone follow-up, medication reconciliation, patient education, home visits, etc. A recent systematic review and meta-analysis showed that interventions are overall effective at reducing readmission.[15] However, the interventions that were effective are complex to implement and resources intensive. Furthermore, most of these interventions studies (27 out of 47) have been performed on specific patient populations such as patients with heart failure or chronic lung disease only, which is not transposable to the majority of medical patients who frequently have multiple chronic conditions. Among the 17 studies that included general medical patients, half targeted elderly patients only. We know however that age is not a risk factor for readmission,[16, 17] and therefore by targeting specifically only the older patients’ interventions may miss the group of patients with an actual high-risk of readmission. Among the 8 remaining randomized control studies in medical patients, 3 studies focused on medication interventions only. But more importantly, none tried to target high-risk patients for readmission. Although all patients deserve a high-quality discharge process that includes, for example, timely hand-off and follow-up appointments, more complex and costly interventions like post-discharge phone calls or home-based transitions coaching should be targeted to the patients who are most likely to benefit. Therefore, efficiently improving transitions in care requires hospitals to target discharge interventions at those patients at higher risk of potentially avoidable readmission. Recently, in a recent randomized controlled trial, Dhalla et al. did try to target high risk patients using the LACE index.[18] However, about 90% of the patients were identified as high-risk using the LACE index. This suggests that the threshold used was too low for the population, or that the LACE score failed to identify the real high-risk patients, which therefore may have contributed to the negative findings.

Identification of patients at high risk for readmission

To identify which are the patients at risk for these readmissions is challenging. It has been showed that clinicians (including nurses, case manager, medical residents, or attendings) are not able to accurately identify which patients are at high risk for readmission.[19] A systematic review in 2011 pointed out the lack of performance and validity of the existing prediction models in readmission.[20] However, we previously developed a prediction model to identify the patients at higher risk for potentially avoidable readmission that has shown good performance in several settings, and is now the most valid and easy to use prediction model (see paragraph 2.2 below).

Gaps in current knowledge

In conclusion, the current gaps in transitional care intervention knowledge are:

- In Switzerland, little is known about the quality of the hospital discharge process, and what are the characteristics of the readmissions, and to our best knowledge none are currently underway.
- The effectiveness of transitional care interventions to reduce readmissions has shown inconsistency.
- We know that the most effective transition care interventions are also the most complex ones, but trials failed to target the medical patients who are most likely to benefit for better effectiveness. To identify those high-risk patients, no trials used one of the best validated, accurate, and easy-to-use tools, such as the "HOSPITAL" score (see paragraph 2.2 below).
- Most of the trials have been performed on specific patient populations, such as patients with either diabetes, chronic obstructive pulmonary disease (COPD), or heart failure. But the practicability of such interventions is limited in real-life because of the high prevalence of patients with multiple chronic medical conditions.
- No interventional studies to reduce readmission have been previously performed in general medical patients in Switzerland.
- The rate and type of post hospital-discharge events is not well-known in Switzerland, as well as the quality of the discharge process, and how it varies according to social factors such as urban-rural environments.
- No simple tool to predict long-term mortality by hospitalized patients

Identification of high-risk patients for potentially avoidable readmission: the HOSPITAL score

We recently derived a prediction model to identify high-risk medical patients for 30-day potentially avoidable readmission to target transitional care interventions accordingly. This clinical decision tool is named "HOSPITAL" and includes variables readily available before discharge (Table 3.1).[21] The score has been now also validated in an international multicenter study with more than 117,000 patients in 4 different countries, including Switzerland, and showed good performance: an excellent overall accuracy (Brier score 0.08), a good discrimination power (C-statistic of 0.72), and an excellent calibration (p value of Pearson goodness-of-fit test of 0.89).[22] A retrospective validation in 3 academic hospitals in Switzerland (Inselspital, CHUV, and HUG) showed good performance with a C-statistic of 0.68.[23] A prospective validation study at the Fribourg Cantonal Hospital showed a C-statistic of 0.70.[24] We also further assessed the performance of the HOSPITAL score specifically for frequent diseases such as pneumonia, heart failure, COPD exacerbation, and myocardial infarction.[25] The performance remained stable with a C-statistic ranging from 0.68 to 0.71 according to the disease. We finally validated a simplified version of the HOSPITAL score, that includes 6 of the 7 variables (number of procedures being left out), and in which the criteria of "discharge from an oncology unit" has been replaced by "discharge from an oncology unit or diagnosis of active cancer".[26] In the simplified version, the risk of readmission was categorized into 2 groups: unlikely and likely to be readmitted, according to the total number of score points (threshold at 4 points). This simplified version showed the exact same performance as the original HOSPITAL score, and therefore can be considered to be used according to the setting. We further validated the simplified score in the phase 1 of the Target-READ (BASEC 2017-00858). Based on preliminary analysis, the simplified score showed overall similar discriminatory power (C-statistic 0.68) in comparison to the original version of the HOSPITAL score (C-statistic 0.66).

Table 3.1: Simplified “HOSPITAL” score to identify the patients at high risk for 30-day readmission

Attribute	Value	Points
Low Hemoglobin level at discharge (< 120 g/l)	yes	1
Discharge from an Oncology service or active cancer diagnosis*	yes	2
Low Sodium level at discharge (< 135 mmol/l)	yes	1
Index admission Type: urgent or emergent (nonelective)	yes	1
Number of hospital Admission(s) during the previous year	0-1	0
	2-5	2
	>5	5
Length of stay ≥ 8 days*	yes	2

* Adapted and validated in Switzerland.[23, 24, 26]

These studies demonstrate the generalizability and validity of the HOSPITAL score.[27] It is currently not only the best validated prediction model for hospital readmission so far, both internationally (USA, Canada, Israel, Denmark) and more locally in Switzerland, but is also an easy-to-use tool. Its performance is higher than the performance of scores previously described in a systematic review.[20] The HOSPITAL score has been also recently shown to be significantly better than the LACE score in a head-to-head comparison.[28] It also performs better than other currently clinical scores used in daily practice, such as those to evaluate the risk of bleeding under oral anticoagulants.[29, 30]

Context

The main objective of the study is to evaluate the effect of a transitional care intervention prioritized to higher-risk medical patients on the composite of 30-day unplanned readmissions and death. Readmissions to the hospital are frequent and costly, and represent a major health issue. Financial incentives have been put in place in several countries including Switzerland to improve the discharge process and reduce readmission rate. In Switzerland, the promotion of coordination of care between the different settings is one of the aim of The Federal Council’s health policy priorities entitled "Gesundheit2020" (Health 2020).[8] While the issue of readmission and quality of discharge process has been identified and incentives started, the methods to reach such aims are still unclear, and innovative high-quality studies with effective interventions are still lacking.

Specific aims and hypothesis

Interventional study among patients at higher-risk for readmission

Primary aim: 1) To evaluate in a randomized controlled trial the effect of a transitional care intervention prioritized to higher-risk medical patients (simplified HOSPITAL score ≥4) on the composite of 30-day unplanned readmissions and death.

Hypothesis: by providing a care transition intervention prioritized to the patients at higher-risk for readmission according to a validated clinical decision tool (HOSPITAL score), the quality of transition of care and the post-discharge adverse events such as readmission will be reduced in this population.

Secondary aims:

- To evaluate the effect of a targeted transitional care intervention to higher-risk medical patients on: hospital utilization, medication adherence, patient’s perspective on quality of transition of care, time to readmission or death (whatever comes first).
- To evaluate the effect of a targeted transitional care intervention on the primary outcome in subgroup population of patients with intermediate (simplified HOSPITAL score of 4 or 5) compared to high risk of readmission (simplified HOSPITAL score ≥6), or who suffer from common chronic diseases (diabetes, chronic heart failure, or COPD).

3.2 Investigational Product (treatment, device) and Indication

The study is not testing any product. The intervention consist of patient education, patient information, and follow-up planning.

The intervention group will receive a standardized transition care intervention (called "TARGET intervention") by a trained nurse as described below (pre-discharge component + 2 post-discharge follow-up phone calls at day 3 and day 14). The pre-discharge component includes patient information, medication reconciliation, patient education, and planning of a first post-discharge primary care physician visit (Table 3.2a). Two follow-up phone calls will be made by a nurse, at D3 (+/- 1 day) and D14 (+/- 1 day), and will include a reinforcement of the patient education, the verification of timely primary care physician visit and that a timely discharge summary is sent to the primary care physician, and review with the patient of the medication list with assessment of potential adverse drug events (Table 3.2b). Adherence to the intervention will be collected by collecting which of the 3 components the patient indeed received (i.e. pre-discharge component, first follow-up phone call, second follow-up phone call). The contact information of the patient will be necessary in order to reach the patient for these 2 follow-up phone calls.

Table 3.2a: Pre-discharge components of the TARGET intervention (within 48 hours before discharge)	
Patient information about her/his diseases	-Summarize to the patient the main diagnoses of the hospital stay, then the already known important comorbidities. Teach back.<br>-Provide standard patient information leaflet of the following disease when relevant: heart failure, coronary heart disease, atrial fibrillation, peripheral artery disease, thromboembolism, stroke, chronic obstructive pulmonary disease, gastrointestinal bleeding, cirrhosis, renal failure, diabetes.<br>-Understanding what to do in the occurrence of new danger signs based on their diseases, and what to do if they occur
Medication reconciliation	-Compare medication list at admission and discharge (medication reconciliation), ask patient about any missing medication from home. Inform treating hospital physician if any discrepancy found.<br>-Review medication's purpose with the patient. Hand out the discharge medication list to the patient. Teach back.<br>-If the patient has an aerosol therapy for COPD, ask the patient to show how to use it.<br>-Provide general explanation about the importance to follow the medication therapy at home as prescribed.
General patient education about lifestyle	-Patient education about general health recommendation: stop smoking and/or alcohol, healthy food, regular physical activity, etc.<br>-Hand out the patient information leaflet about general lifestyle recommendations.
Dependence level	-Check the Activity of Daily living of the patient (using the Katz score). If the score is >6 points inform the medical team.<br>-Ask about possible support at home by next of skin, or nurse at home.
Follow-up care planning	-Organize medical support: identify the primary care physician, and make appointment for a follow-up visit as soon as possible (ideally within 7 days following discharge). If the patient doesn't have a primary care physician, suggest a few primary care physicians. Hand out an appointment sheet with: name and phone number of the primary care physician.<br>-Insure a timely discharge summary sent to the primary care physician shortly after hospital discharge. Additionally, to the discharge summary, the primary care physician will be notified by a separate sheet on the higher risk of readmission identified by her/his patient.

Barriers	-Review any barriers to a safe discharge, including patient's ability to carry out the discharge plan, willingness and ability to pick up their medications and to take them; and willingness and ability to keep their follow-up appointments. Take actions to reduce these barriers and to communicate these barriers to the medical team.<br>-Give the opportunity to the patient to ask for questions, and rely them to the medical team if needed.

Table 3.2b: Post-discharge follow-up phone call of the TARGET intervention (day 3 and 14)	
1. Health condition:	Ask whether condition improved, worsened, or recurred. Ask about any reduced walking distance that is possible since hospital discharge. Ask about capacity to prepare meals. Ask for weight trend over the last days/weeks, blood pressure, glucose monitoring data when relevant.
2. Primary care physician visits:	Check when was or is scheduled an appointment to the primary care physician. In case of health condition or medication issue, the patient will be encouraged to contact his primary care physician.
3. Medications:	Review list of current medications and ask about any discrepancy. Monitor significant adverse events for high alert medicines (anticoagulants, narcotics and opiates, insulin, and sedatives because they represent areas of greatest harm and greatest opportunity for improvement). The most common types of harm associated with these medications include hypotension, bleeding, hypoglycemia, delirium, lethargy, and oversedation. Underline the importance of following the medication as prescribed, and to seek for primary care physician advice in case of any question or side effects.
4. Patient education:	Refresh about general health recommendations (importance of medication compliance, healthy food, regular physical activity, etc.), and more specific but simple recommendation about the diseases of the patients if part of the patient info leaflets (heart failure, coronary heart disease, atrial fibrillation, peripheral artery disease, thromboembolism, stroke, chronic obstructive pulmonary disease, gastrointestinal bleeding, cirrhosis, renal failure, diabetes).

3.3 Preclinical Evidence

Not applicable.

3.4 Clinical Evidence to Date

Not applicable. See point 3.1 Background and rationale for existing evidence about interventions to reduce hospital readmission (under "Preventability of readmission and interventions)."

3.5 Dose Rationale / Medical Device: Rationale for the intended purpose in study (pre-market MD)

Not applicable.

3.6 Explanation for choice of comparator (or placebo)

The comparator will be usual care. The purpose of this study is to evaluate effects on clinical outcomes of the above described intervention in comparison to standard care. Therefore, we compare a standardize intervention to the local usual care. A hospital would start such intervention when it has

been shown to improve care in comparison to what is currently done. Any type of “shame” intervention may influence in an undetermined way the risk of readmission, and it will be then difficult to answer the study question.

3.7 Risks / Benefits

Potential benefits

Readmissions to the hospital are frequent and costly, and represent a major health issue. Although incentives have been already put in place in Switzerland (as in the US), few has been concretely made in the field to improve the transition of care of the patients in Switzerland. Transition of care between the hospital and the ambulant setting is a known period at risk for adverse events, and has recognized potential for quality improvement. In Switzerland, the promotion of coordination of care between the different settings is one of the aim of The Federal Council’s health policy priorities entitled "Gesundheit2020" (Health 2020). While the issue of readmission and quality of discharge process has been identified, the methods to reach such aims are still unclear, and innovative high-quality studies with effective interventions are still lacking. Efficiently improving transitions in care requires hospitals to target discharge interventions at those patients at high-risk of potentially avoidable readmission. This study aims primarily to evaluate the effect of a transitional care intervention prioritized to higher-risk medical patients according to the simplified HOSPITAL score on the composite of 30-day unplanned readmissions and death.

This research project is innovative and promising in several ways to help address many gaps in current knowledge. 1) we will include all medical patients, regardless of the cause of admission, and will include many patients with multiple chronic conditions as in real-life, which will provide more generalizable results; 2) we will target the higher risk patients using one of the most accurate and validated tool, the HOSPITAL score in order to better allocate transition care resources and to improve effectiveness. This score has been now widely validated, including in Switzerland; 3) we will perform the first randomized controlled trial to test a transition care intervention in medical patients in Switzerland; 4) finally, we will look at the patient’s perspective about transition care, and also the causes of readmission in Switzerland, but also evaluate how the readmissions varies according to rural vs urban environment, and according to the type of health insurance.

The main implications of the study are the following:

1) We may demonstrate the efficacy in Switzerland of a set of interventions that separately showed some promising results in other countries (geographical transportability). Although information from existing studies in other countries might be valuable, our health care system and our population is different, and effectiveness of such intervention must be tested before to be widely implemented. One of the innovative part of the study is to target intervention to the patients at higher risk for readmission using a validated tool.
2) We will validate the clinical impact of the simplified HOSPITAL score, i.e. its effectiveness to effectively help reducing the readmission in high-risk patients when implemented together with a discharge intervention. This would give to the HOSPITAL score the highest degree of evidence (level I out of IV) in validation of prediction models.[31]
3) To reduce post hospital-discharge adverse events, such as 30-day readmission or death, in a large population of medical patients, who have typically multiple chronic conditions.
4) To better allocate of transition care interventions to those who are most likely to benefit, i.e. the patients at higher risk for readmission, using a validated tool.
5) To improve the discharge process experienced by the patients, by providing a better follow-up after discharge and a better coordination between the hospital and the primary care settings for better treatment continuity.

All the main actors of the health care system may benefit from the study findings: 1) The patients may benefit from a better quality of the transition of care and a reduced risk of post-discharge adverse events such as readmissions; 2) The hospitals may use the intervention in order to improve their quality of care process, and reduce the financial penalties due to costs of readmissions; 3) The primary care providers may be helped to avoid losing contact with their high risk patients in the post-hospital discharge period, in order for them to intervene before an adverse event arise; 4) The decision makers in Switzerland will

be informed on the quality of transition of care, and see the potential benefits of a transition of care intervention between settings, with a better care coordination.

Potential risks

All the component of the intervention are known procedures already provided sometimes to the patients. The study here is grouping in a standard package the most promising interventions together. There is therefore no known risks of such intervention. The main innovative part of the study is to target a standard package of patient support to the patients who are most likely to benefit, in order to make the intervention overall more successful. In conclusion, we are not expecting any risks for the patients, other than the confidentiality breach inerrant to any clinical study. To avoid any confidentiality breach, the data will be collected directly and stored in a web-secured database (Redcap). Each personal study will be provided with a personal access and identifiable login to Redcap. The signed informed consents and the discharge letters from the hospital will be stored in a binder and securely locked in a closet closed with a key at each hospital.

3.8 Justification of choice of study population

The study population will consist of adult inpatients discharged alive home (or nursing home). Only patients discharged from a medical department will be recruited, since the simplified HOSPITAL score used to identify the higher risk patient for readmission has been developed and validated for this population only. Among those, the project is to focus only on the patients with the highest risk of 30-day readmission based on the HOSPITAL score. The HOSPITAL score and its simplified version have been widely and internationally validated and are currently the most valid tool to identify the patients at high risk of readmission. Potential patients will be identified through active screening by dedicated trial staff in each centre.

The risk of readmission exist only if a patient has been: 1) hospitalized; 2) discharged alive. This explain the choice of the study population. Each year, more than 1.4 million acute adult hospitalizations arise in Switzerland, among which about 12-15% are 30-day readmissions.[32] Many complications can occur as patients are discharged from hospitals to the ambulatory setting, which lead to unnecessary distress for the patients and costly readmissions.[3, 4]

Because patients who are transferred to another acute care facility, or a rehabilitation center have are in a structure with a certain level of medical care available, these patients will have their risk of readmission artificially modified. Moreover, the intervention aims mainly to involve the patients in their own care and health recovery, which obviously is not something that can be done or the effect measured as long as they are in a medical facility. Therefore, patients transferred to another acute care facility or rehabilitation center will be excluded from the study.

Due to the nature of the intervention that emphasize in great part the involvement of the patients in their health care, patients who have cognitive troubles such as dementia will not be included. Because the intervention is about patient education the nurse must be able to communicate in her own language to the patient. Therefore only patient speaking French will be included at the site of Fribourg, Lausanne and Neuchâtel, and patients speaking French or German at the site of Biel. A translator is not feasible because the time between the screening of the patient (medical decision of discharge) and the discharge of the patients is most often limited to a few hours and therefore doesn't allow to contact external translators.

4. STUDY OBJECTIVES

4.1 Overall Objective

The overall objective of this RCT is to evaluate whether a standardized intervention targeted to the patients at higher risk of readmission will lead to a reduction in the risk of 30-day unplanned readmission or death.

4.2 Primary Objective

Primary aim: 1) To evaluate in a randomized controlled trial the effect of a transitional care intervention prioritized to higher-risk medical patients on the composite of 30-day unplanned readmissions and death.

Hypothesis: by providing a care transition intervention prioritized to the patients at higher-risk for readmission according to a validated clinical decision tool (simplified HOSPITAL score), the quality of transition of care and the post-discharge adverse events such as readmission will be reduced in this population.

4.3 Secondary Objectives

Secondary aims:

- To evaluate the effect of a targeted transitional care intervention to higher-risk medical patients on: hospital utilization, medication adherence, patient's perspective on quality of transition of care, time to readmission or death (whatever comes first).
- To evaluate the effect of a targeted transitional care intervention on the primary outcome in subgroup population of patients with intermediate (simplified HOSPITAL score of 4 or 5) compared to high risk of readmission (simplified HOSPITAL score ≥6), or who suffer from common chronic diseases (diabetes, chronic heart failure, or COPD).

4.4 Safety Objectives

Not applicable.

5. STUDY OUTCOMES

5.1 Primary Outcome

The primary study outcome will be the number of patients who have an unplanned readmission or die within 30 days after discharge.

The primary outcome will be the composite of first unplanned readmission or death within 30 days after discharge of index admission (inclusion time). The unplanned readmission will be defined as a non-elective hospitalization that happens within 30 days after discharge from the index hospital discharge to any division of any acute care hospital (i.e. not only the hospital where the patient was originally discharged). Elective hospitalization will be defined as a non-urgent hospitalization scheduled at least 1 day prior to the admission day. Death will be defined as any death occurring within 30 days after discharge. We will further collect the cause of readmission or death. Death is the worst outcome after a hospital discharge. It is common practice in this domain to include both readmission and death as a composite outcome, because patients who died may well have been readmitted if they hadn't died.[18, 33-35]

In order to collect thoroughly the primary outcome and avoid missing outcomes, the outcomes will be assessed through 5 steps (see below under section 9.2.1).

5.2 Secondary Outcomes

<u>Secondary outcomes:</u>

- Individual components of the primary composite outcome: 30-day unplanned readmission, or 30-day mortality.
- Time to first unplanned readmission or death.
- Main cause of readmission or death.
- Post-discharge health care utilization within 30 days after discharge from index hospitalization: number of hospital readmission and total number of days of hospitalizations within 30 days, number of emergency room visits and primary care provider visits.
- Medication adherence: 4-item Morisky Medication Adherence Scale (MMAS-4).[36-38]
- Patient's perspective (satisfaction) on quality of transition of care between hospital and home: Three-Item Care Transition Measure (CTM-3).[39]
- Costs of readmission.

Medication adherence will be evaluated using the Morisky 4-item Medication Adherence Scale (MMAS-4).[36-38] This instrument measures patients' self-reported adherence to their medications over the last 4 weeks through 4 simple questions and has been widely validated. These questions are the following: 1) Do you ever forget to take your medicine? 2) Are you careless at times about taking your medicine? 3) When you feel better do you sometimes stop taking your medicine? 4) Sometimes, if you feel worse when you take your medicine, do you stop taking it? Each question can be answered by "yes" (1 point) or "no" (0 point). A patient is considered to have a high adherence to the medications if the total score is zero, a medium adherence for score between 1 and 2, and a low adherence for score of 3 or 4. We will estimate the proportion of patients with high adherence, i.e. with a score of 0.

The 3-Item Care Transitions Measure (CTM-3) is a three-question survey that is a hospital level measure of the quality and effectiveness of the transition or discharge from an acute care hospital.[39] The CTM-3 measures whether or not patients' preferences were followed during their care (patient centered care), measures patients understanding of their own role in their care and their discharge medications. The 3 items are: 1) The hospital staff took my preferences and those of my family or caregiver into account in deciding what my health care needs would be when I left the hospital; 2) When I left the hospital, I had a good understanding of the things I was responsible for in managing my health; 3) When I left the hospital, I clearly understood the purpose for taking each of my medications. We will measure the proportion of patients who are responding positively to all 3 items 30 days after discharge of index

admission.

In order to collect thoroughly the outcomes and avoid missing data, the secondary outcomes will be assessed at the same time and through the 5 same steps as the primary outcome. However, the medication adherence (MMAS-4) and patient's perspective (CTM-3) will only be collected from the patient her/himself.

5.3 Other Outcomes of Interest

Not applicable.

5.4 Safety Outcomes

Not applicable.

6. STUDY DESIGN

6.1 General study design and justification of design

The Target-READ is a national multicenter single-blinded randomized controlled trial design among adult inpatients discharged home from the medical department of one of the 4 participating centers (Biel, Fribourg, Lausanne, Neuchâtel).

All medical patients will be screened for eligibility as soon as their hospital discharge is planned. After a baseline evaluation for all inclusion and exclusion criteria, each enrolled participant of all sites will be randomized centrally to the intervention or usual care group using a computer-generated randomization list (allocation ratio 1:1) stratified by discharge site and by readmission risk category according to the simplified HOSPITAL score (intermediate vs high risk, i.e. a HOPSITAL score of 4 or 5 vs ≥6), with randomly varying block sizes of 2, 4, and 6.

Given the nature of the intervention, it will be not possible to blind patients and study nurses. However, treatment allocation in the database will be coded (1/2) and the study nurses collecting the outcomes or working on data cleaning and the statistician performing the analysis will be blinded to the group allocation. Patients will be specifically asked not to mention their group allocation in the final assessment. The statistician will be using the coded variable to prepare a blinded analysis report. Only after discussion of the results with the PI and closing of the database, the code will be broken and a final unblinded analysis report will be generated.

The number of participants to be included will be at least 690 per arm (see below under point 7.1 and 11.2 for more details).

The study will follow the CONSORT statement,[40] and the intervention will be described according to the TIDieR checklist and guide.[41]

Figure 6.1: Study design

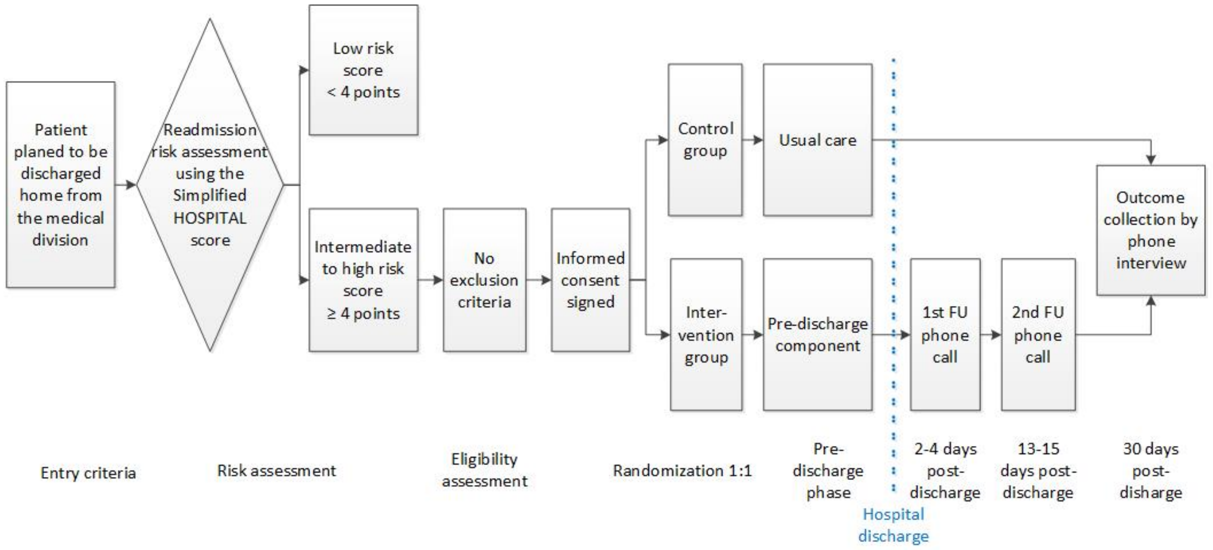

6.2 Methods of minimising bias

Selection bias is considered the most relevant biases in this trial. Screening of all consecutive adult patients about to be discharged home will be performed to avoid selection bias.

Patients will be centrally randomized into the intervention or control group (see section 6.1 above). Given the nature of the intervention, it will be not possible to blind patients and study nurses (see section 6.1 for details).

6.2.1 Randomisation

See section 6.1 for details about randomization.

6.2.2 Blinding procedures

See section 6.1 for details about blinding procedures.

6.2.3 Other methods of minimising bias

A limitation of the study design is the potential for selection bias. In particular, patients who are refusing to participate might have a different risk of readmission than the patients who are willing to participate. To avoid a selection bias, a careful monitoring of recruitment is essential. Not to be able to compare some basic characteristics between the patients who refused to participate and those who agreed to participate is a limitation that would reduce the scientific validity of the entire study.

Some baseline characteristics are thus recorded in the database for all ca. 18'000 subjects of the target population irrespective of whether they will participate in the study or not. This includes the following data: subject ID, screening date, first three inclusion criteria, age. If a subject fulfils the first three inclusion criteria, the following additional data will be recorded: single variables of the HOSPITAL score (i.e. hemoglobin and sodium levels, elective vs. non-elective admission, cancer diagnosis, number of previous hospitalizations in the last 12 months, length of stay). If, according to the HOSPITAL score, the subject is at high risk for readmission, in addition, the exclusion criteria will be recorded. Such data will not be collected from subjects with documented refusal for further use of their data.

Recording these (routine) data in the database is considered as "further use" according to Article 33 (HRA).

6.3 Unblinding Procedures (Code break)

See section 6.1 for details about unblinding procedures.

7. STUDY POPULATION

7.1 Eligibility criteria

<u>Inclusion criteria:</u>

- Adult patients planned to be discharged home or nursing home.
- Discharge alive from a medical department of one of the four participating centers (Biel, Fribourg, Lausanne, Neuchâtel).
- Hospital stay of at least 24 hours.
- Patient at higher risk of 30-day readmission based on the simplified HOSPITAL score.

<u>Exclusion criteria:</u>

- Previous enrolment into the current study (Target-READ phase 2).
- Not living in the country in the next 30 days.
- No phone to be reached at.
- Not speaking French or German (depending on the site).
- Refusal to participate, or unable to give consent (including cognitive troubles defined as a disorientation in time and space).

Figure 7.1a: Detailed inclusion process of Phase II

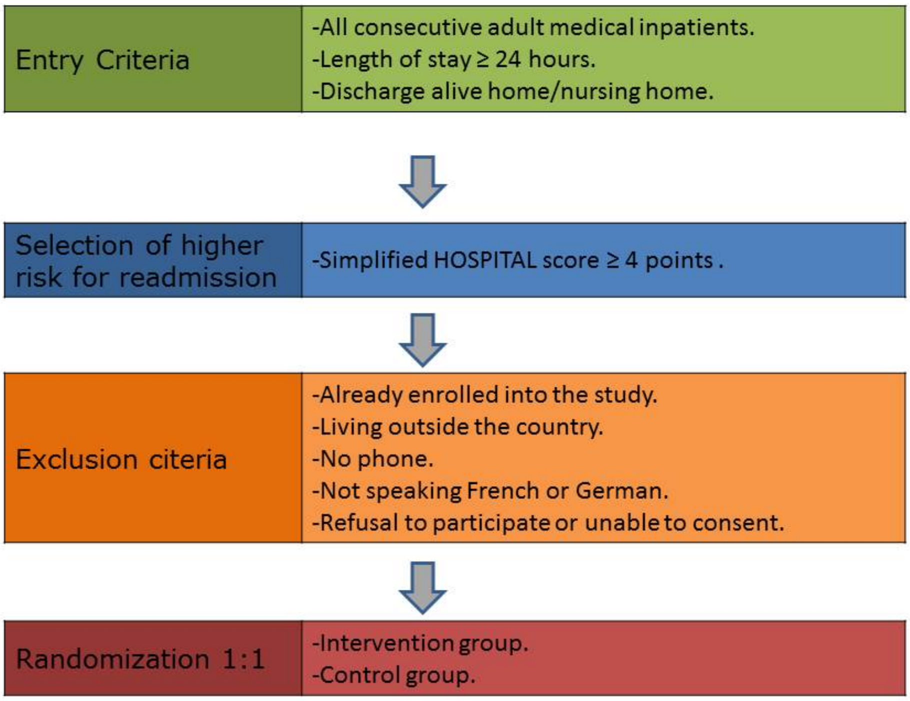

Figure 7.1b: Projected study flow-chart with estimation of the number of included and excluded patients.

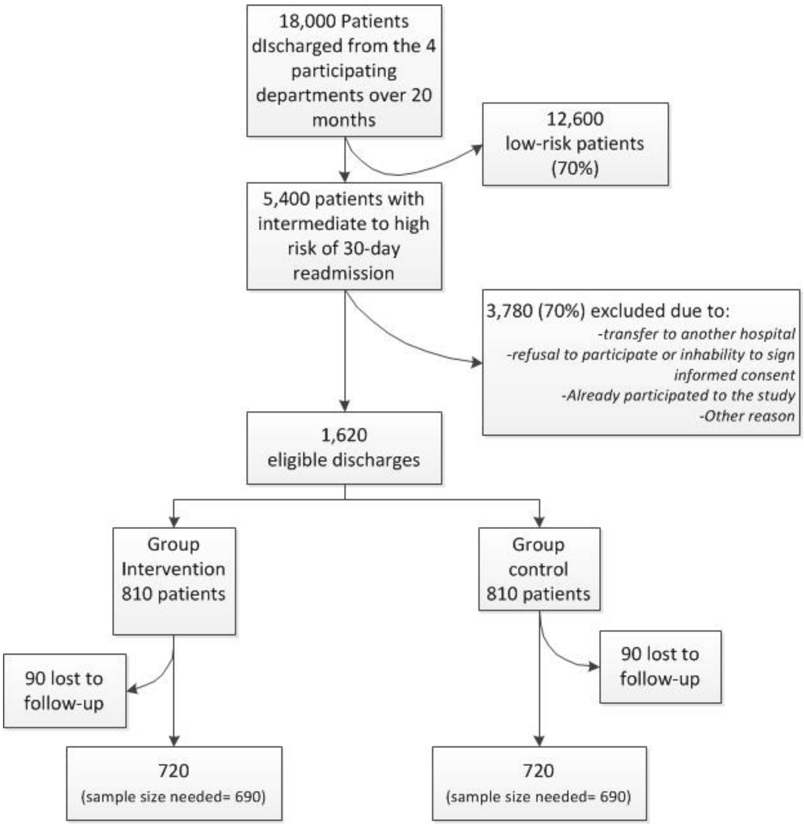

7.2 Recruitment and screening

All patients announced to be discharged soon by the medical team of each participating center will be screened for eligibility. Screening logs will be kept at each site and basic coded information (gender, age, risk of readmission based on HOSPITAL score, etc.) regarding each screened patient will be entered in the database. Variable required to calculate the HOSPITAL score (hemoglobin level, sodium level, diagnosis of cancer, number of hospitalization in the last 12 months, mode of admission, and length of stay) will be entered in the database in order to automatically and therefore reliably calculate the HOSPITAL score. Because of the great expenditure approaching 18'000 subjects for consent to collect these data, and given the importance collecting these data to minimize any selection bias (see section 6.2.3), referring to HRA Art. 34, we will refrain from asking these subjects for consent to collect these data. Such data will not be collected from patients with documented refusal collecting such

Screening data will be evaluated on a regular basis by an independent person to detect possible selection bias.

Only patients at high risk for readmission will be included. The determination of the risk of readmission will be based on the valid prediction model "HOSPITAL" score. This score requires the collection of 7 variables (see section 3.1, Table 3.1). All the information needed to calculate the score will be drawn directly from the electronic health record, no additional measurement outside usual care will be made (the score will be calculated only with available usual care data available at time of screening). No compensation nor payment will be made to the participants.

7.3 Assignment to study groups

See section 6.1 for randomization.

7.4 Criteria for withdrawal / discontinuation of participants

Patients must be withdrawn from the study for any of the following reasons:

- Patient refuses to continue
- Patient is lost to follow-up

Patients may be withdrawn from the study at the discretion of the Investigator based on his or her clinical judgment, but only in exceptional situations. The eCRF should be completed accordingly, providing the primary reason for discontinuing the study prematurely.

8. STUDY INTERVENTION

8.1 Identity of Investigational Products (treatment / medical device)

The intervention will take place in the initial hospital admission (Index hospitalization) defined as the hospitalization in which the patient inclusion took place, and will be pursued after patient hospital discharge. The TARGET intervention has 2 components, one during the hospitalization before the hospital discharge (pre-discharge component), and one after the hospital discharge (post-discharge component).

The pre-discharge component of the TARGET intervention consists of the six following steps:

1. Patient information about her/his diseases.
2. Medication reconciliation.
3. General patient education about lifestyle.
4. Dependence level assessment.
5. Follow-up care planning.
6. Assessment of possible barriers of patient's ability to carry out the discharge plan.

The following documents will be handed to the participants in the intervention group:

Who	What	Name of the file in BASEC
Each participant in the intervention arm	-Information sheet about its participation to the study along with next appointment to the primary care physician.<br>-General health hygiene information.	Binder_patientinfo.ALLintervgrp<br>_TargetIntervention
According to the disease of the patient	-one or two-page leaflet about frequent diseases.	Binder_patientinfo.Diseases<br>_TargetIntervention
Primary care physician	-Information sent directly to the primary care physician of the study participation of the patient and her/his high risk of being rehospitalized	DX_Infomédecin

For details about each steps see table 3.2a under section 3.2.

The post-discharge component of the TARGET intervention consists of the 4 following steps:

1. Review of the health condition
2. Verification of the primary care physician follow-up.
3. Review the current list of medications.
4. Patient education.

For details about each steps see table 3.2b under section 3.2.

Every study nurses will be receiving the same training for the TARGET intervention. The training will be led by the sponsor, individually and in group, as well as theoretically and practically.

Contact information including name, address and phone number of the patient will be necessary to contact the patient for the 2 follow-up phone call that are part of the intervention, and to collect the outcome at 30 days.

8.1.1 Experimental Intervention (treatment / medical device)

Not applicable.

8.1.2 Control Intervention (standard/routine/comparator treatment / medical device)

Participants randomized in the control group will receive information and organization of the post-discharge care in accordance with local usual care.

8.1.3 Packaging, Labelling and Supply (re-supply)

Not applicable.

8.1.4 Storage Conditions

Not applicable.

8.2 Administration of experimental and control interventions

8.2.1 Experimental Intervention

The intervention will be conducted before hospital discharge. Inclusion criteria regarding patient availability have to be met in order to ensure feasibility of the intervention. The whole interventional process is described under section 3.2 above.

8.2.2 Control Intervention

Patients being assigned to the control arm will be treated in accordance with standard care. They will receive in addition to the patient information consent an information sheet stating that they have been selected in the control group.

8.3 Dose / Device modifications

Not applicable.

8.4 Compliance with study intervention

During the intervention the discussion with the patient is expected to enhance compliance. We will measure which part of the intervention each participant were exposed to (pre-discharge component, 1st and 2nd post-discharge components).

8.5 Data Collection and Follow-up for withdrawn participants

A study patient can withdraw his/her consent to participate in the trial at any time and without any declaration of reason. Data collected until the time point of withdrawal will remain in the database and will be used for analysis.

8.6 Trial specific preventive measures

Not applicable.

8.7 Concomitant Interventions (treatments)

Not applicable.

8.8 Study Drug / Medical Device Accountability

Not applicable.

8.9 Return or Destruction of Study Drug / Medical Device

Not applicable.

9. STUDY ASSESSMENTS

9.1 Study flow chart(s) / table of study procedures and assessments

	**Index hospitalization[1)]**		**Follow-up[2)] (post-discharge)**		
	Screening (before inclusion)	Visit 1 (inclusion, day 0)	Visit 2 (day3 ±1)	Visit 3 (day 14 ±1)	Visit 4 (day 30 +15)
**Assessment**	**Pre-study screening**	**Baseline and pre-discharge intervention (only intervention group)**	**Post-discharge intervention 1 (only intervention group)**	**Post-discharge intervention 2 (only intervention group)**	**Outcome collection**
Demography	X				
Eligibility	X				
HOSPITAL score	X				
Informed consent		X			
Randomization		X			
Baseline characteristics		X			
Medical history		X			
Medication		X			
Activity of daily living (ADL Katz score)		X			
Exposition to intervention		X	X	X	
Medication discrepancy			X	X	
Adverse drug event			X	X	
Follow-up care plan followed					
Primary outcome: unplanned readmission or death					X
Secondary outcomes:					
Number of unplanned readmission					X
Number of days of hospitalization within 30 days					X
Diagnoses at readmission or cause of death					X
Number of emergency department visits					X
Number of PCP visits					X
Medication adherence (4-MMAS)					X
Discharge satisfaction (3-CTM)					X
Costs of readmission					X
Letter of discharge of the					X

unplanned readmission					
End of study					X

[1)] Assessment based on electronic health record and/or in person.

[2)] Assessment by phone call

Laboratory parameters: the simplified “HOSPITAL” score includes 2 lab values (haemoglobin and sodium level). These values are part of routine care. Only available data in the patient records will be used. The study participants will never get an extra blood draw solely for the purpose of the study to measure these parameters if they would not be available in the patient records at time of discharge.

9.2 Assessments of outcomes

9.2.1 Assessment of primary outcome

The primary outcome will be collected at least 30 days after inclusion via phone interview by a blinded study nurse outside the participating site. The blinded study nurse will have only access to: 1) the contact information from an Excel file; 2) the outcome collection form in Redcap. In that way the study nurse will be unaware of the group allocation of the participant. The participant will be asked in the patient informed consent and at the beginning of the phone call to not divulgate her/his group allocation.

In order to collect thoroughly the primary outcome and avoid missing outcomes, the outcomes will be assessed through 5 steps:

1. Phone interview with the study participant (3 phone calls attempts on at least 2 different days).
2. If study participant her/himself is not reachable: phone interview with a next of kin of the study participant (2 phone calls attempts on at least 2 different days).
3. If study participant her/himself or next of kin are not reachable: phone interview with the primary care physician of the study participant.
4. If information is still missing, the nurse will call the study nurse of the local site to look for any outcome event that would be reported in the electronic health system.
5. In any case of a readmission: the medical report of the readmission will be collected to assess the exact place, time, length, and cause of hospital readmission.

9.2.2 Assessment of secondary outcomes

The secondary outcomes will be collected by phone interview at the same time of the primary outcome and with the same procedure.

Number of unplanned readmission: if the patient, the next of kin or the primary physician report a readmission, the date of readmission and the length of stay will be asked. In any case, the medical report of the readmission will be collected to double check the validity of the information and obtain the causes of readmission.

Number of days of hospitalization within 30 days: if the patient, the next of kin or the primary physician report one or more readmission, the total number of days spent at the hospital will be measured through the electronic health system for the participating site, or by calling the hospital if a readmission occurred outside the 4 participating sites.

Diagnoses at readmission or cause of death: if the patient, the next of kin or the primary physician report

a readmission, the medical report will be collected. If a death is identified, the medical report will be collected if the death occurred at the hospital, otherwise the cause will be obtain from the primary care physician, or the certificate of death.

Number of emergency visits within 30 days: the patient, the next of kin or the primary physician will be asked about the number of visit to any emergency room (any clinic or hospital).

Number of primary care physician visits: the patient, the next of kin or the primary physician will be asked about the number of visit to any treating physician (generalist of specialist).

Medication adherence: only the patient (or a caring person) can answer a 4-item questionnaire about medication adherence (see section 5.2. for the details about the questionnaire MMAS-4.

Patient's perspective (satisfaction) on quality of transition of care between hospital and home: only the patient (or a caring person) can answer the questionnaire 3-Item Care Transition Measure (CTM-3, see section 5.2 for details).

Costs of readmission: the costs of the readmission will be collected at the hospital site.

9.2.3 Assessment of other outcomes of interest

Not applicable.

9.2.4 Assessment of safety outcomes

Not applicable.

9.2.4.1 Adverse events

Not applicable.

9.2.4.2 Laboratory parameters

Not applicable.

9.2.4.3 Vital signs

Not applicable.

9.2.5 Assessments in participants who prematurely stop the study

Each participant will be asked for the reason of premature withdrawn of the study participation.

9.3 Procedures at each visit

9.3.1 Screening (Visit 0)

The following activities and assessments have to be performed at the screening visit (before inclusion):

- Assess eligibility
- Document demographics
- Assess the risk of 30-day readmission based on the simplified HOSPITAL score

9.3.2 Visit 1: Patient inclusion and pre-discharge intervention

The following activities and assessments have to be performed at the first visit:

- Confirm the eligibility
- Obtain the informed consent
- Randomize the participant
- Obtain contact information needed for visits 2, 3, and 4 (collected separately from all other variables).
- Obtain the baseline characteristics
- Document medical history
- Document medication
- Obtain level of autonomy if available
- Provide the participant with the pre-discharge intervention (include mainly organization of a follow-up visit by the primary care physician, patient education).
- Provide the participant with the disease info leaflet when relevant (available for the following diseases: heart failure, coronary heart disease, atrial fibrillation, peripheral artery disease, thromboembolism, stroke, chronic obstructive pulmonary disease, gastrointestinal bleeding, cirrhosis, renal failure, diabetes).

9.3.3 Visit 2: Post-discharge follow-up phone call 1 (Day 3 ±1)

The following activities and assessments have to be performed at the second visit only for the participants allocated to the intervention group:

- Ask the patient about her/his general health condition in comparison to hospital discharge.
- Compare the current medication list with the medication list from hospital discharge.
- Document any suspicion of frequent drug adverse events such as: hypotension, bleeding, hypoglycemia, delirium, lethargy, and oversedation.
- Re-inforce patient education and importance of the treatment adherence.
- Assess if a follow-up visit by the primary care physician happened.

9.3.4 Visit 3: Post-discharge follow-up phone call 2 (Day 14 ±1)

The following activities and assessments have to be performed at the third visit (same as visit 2) only for the participants allocated to the intervention group:

- Ask the patient about her/his general health condition in comparison to hospital discharge.
- Compare the current medication list with the medication list from hospital discharge.
- Document any suspicion of frequent drug adverse events such as: hypotension, bleeding, hypoglycemia, delirium, lethargy, and oversedation.
- Re-inforce patient education and importance of the treatment adherence.
- Assess if a follow-up visit by the primary care physician happened.

9.3.5 Visit 4: Outcome collection (Day 30 up to day 45)

The following activities and assessments have to be performed at the fourth visit for all included patients:

- Document any 30-day unplanned readmission or death
- Document the number of primary care physician visits, the number of emergency department visits.
- Conduct questionnaire for medication adherence (4-MMAS), and patient's perspective on the quality and effectiveness of the transition or discharge from an acute care hospital (CTM-3).
- Collect discharge letter (causes of readmission) and costs of hospitalization of any first unplanned 30-day readmission.

10. SAFETY

10.1 Drug studies

Not applicable.

10.2 Medical Device Category C studies

Not applicable.

10.3 Medical Device Category A studies

Not applicable.

10.4 Other clinical studies

The Sponsor's SOPs provide more detail on safety reporting.

During the entire duration of the study, all adverse events will be fully investigated and assessed for seriousness and potential relationship with the TARGET intervention. If a serious adverse event (SAEs) occurs and it cannot be excluded that the event is attributable to the intervention, it is fully documented in source documents and in an SAE report form. Study duration encompasses the time from when the participant signs the informed consent form until the last protocol-specific procedure has been completed.

10.4.1 Definition and assessment of serious adverse events and other safety related events

An **Adverse Event (AE)** is any untoward medical occurrence in a clinical investigation participant administered an intervention and which does not necessarily have a causal relationship with the intervention. An AE can therefore be any unfavourable and unintended sign (including an abnormal laboratory finding), symptom, or disease temporally associated with the intervention, whether or not related to the intervention. [ICH E6 1.2, adapted]

A **Serious Adverse Event (SAE)** is classified as any untoward medical occurrence that:

- requires inpatient treatment not envisaged in the protocol or extends a current hospital stay;
- result in permanent or significant incapacity or disability;
- is life-threatening or results in death; or
- causes a congenital anomaly or birth defect.

In addition, important medical events that may not be immediately life-threatening or result in death, or require hospitalisation, but may jeopardise the patient or may require intervention to prevent one of the other outcomes listed above should also usually be considered serious.

SAEs should be followed until resolution or stabilisation. Participants with ongoing SAEs at study termination will be further followed up until recovery or until stabilisation of the disease after termination.

<u>Assessment of Causality</u>

Both Investigator and Sponsor make a causality assessment of the event to the TARGET intervention. A causal relationship towards the intervention should be rated as follows:

- **Not related:** The event is definitely not associated with the intervention; a relationship can be ruled out.
- **Possibly related:** The relationship between the intervention and the event is possible, but other causes cannot definitely be ruled out.
- **Related:** The event is definitely associated with the intervention.

Assessment of Severity

SAE severity will be classified as either mild, moderate, or severe.

10.4.2 Reporting of serious adverse events (SAE) and other safety related events

Reporting of SAEs

If, in the course of the trial, an SAE occurs and it cannot be excluded that the event is attributable to the intervention under investigation, the event must be reported immediately and within a maximum of 24 hours of learning of its occurrence to the Sponsor using the SAE report form provided by the Sponsor.

The form should be sent via email to the following address: Jacques.donze@insel.ch

The Sponsor will re-evaluate the SAE and return the form to the site.

All of the above events are reported to the coordinating and local Ethics committee(s) (as applicable) within 15 days.

Reporting of Safety Signals

All suspected new risks and events that require safety-related measures, i.e. so called safety signals, must be reported to the Sponsor within 24 hours. The Sponsor must immediately inform all participating Investigators about all safety signals.

The Sponsor must report the safety signals to the coordinating and local Ethics committee(s) (as applicable) within 7 days.

Reporting and Handling of Pregnancies

Any pregnancy must be reported to the sponsor at once.

Periodic reporting of safety

An annual safety report is submitted by the Sponsor once a year to the responsible Ethics committee and is also provided to all participating Investigators.

10.4.3 Follow up of (Serious) Adverse Events

Participants with any reported ongoing SAE at the last scheduled study contact will be followed until resolution of the event or a stabilized condition of the subject has been achieved or until the subject is lost to follow-up.

11. STATISTICAL METHODS

The statistical analysis of the trial will be done at Clinical Trials Unit (CTU) Bern by a statistician blinded to the allocation. This process is defined in standard operating procedures. After start of the trial but before recruitment ends, a statistical analysis plan will be written. The plan will determine all necessary data preparation steps (e.g. additional validations, generation of new variables), definitions (e.g. analysis sets), and statistical analyses (e.g. models and outputs such as tables and graphs). All statistical analyses will be presented as effect measure plus 95% confidence interval (CI). A significance level of 5% will be used.

11.1 Hypothesis

The statistical null hypothesis is that there is no difference in the proportion of 30-day unplanned hospital readmissions and death between the intervention and control group. The alternative hypothesis is that there is a difference in the proportion of unplanned hospital readmissions between both groups. Therefore, the statistical framework within which this trial is conducted is that of a superiority trial.

11.2 Determination of Sample Size

The intervention phase (randomized controlled trial) will be restricted to the eligible patients who are at intermediate to high risk of 30-day readmission according to the simplified HOSPITAL score, i.e. ≥4/13 points (Figure 3.1). We estimate to have around 18,000 patients discharged during the 20-month study period, 30% of which (5,400) will be at higher-risk for a 30-day readmission or death.

Because we will target patients at higher risk of readmission, we hypothesize that the intervention could reduce readmission by 25%, i.e. more than the 18% reduction found in a recent meta-analysis where patients were mostly not at high risk for readmission.[15] Based on previous findings, the expected 30-day readmission and death rate for patients at intermediate or high risk according to the simplified HOSPITAL score is around 27%. Allowing for 10% loss to follow-up, we determine that we will need 1,380 patients for the study to have 80% power, i.e. 690 in each arm. According to a conservative estimation, the sample size is expected to be reached within the study time frame.

11.3 Statistical criteria of termination of trial

There is no interim analysis planned, i.e. there are no stopping rules on the individual or trial level.

11.4 Planned Analyses

11.4.1 Datasets to be analysed, analysis populations

The main analysis set will include all randomized patients according to the intention-to-treat principle. A secondary analysis set will include patients treated per-protocol, i.e. all patients that received the pre-discharge component plus at least one of the 2 post-discharge follow-up phone calls of the intervention and completed the study according to protocol. A third analysis set will exclude all patients with readmissions within the first 24 hours.

11.4.2 Primary Analysis

The primary analysis will be a comparison between two groups according to the intention-to-treat principle, i.e. all randomized patients will be analyzed in the allocated group regardless of any protocol violations such as cross-overs or early discontinuations of the intervention.

The primary outcome will be reported as a proportion of patients in each group and compared between the groups by a risk difference with 95% CI and a chi squared test. Thirty-day deaths, dichotomized

patient satisfaction and medication adherence will be analyzed in the same way. The proportion of 30 day unplanned readmissions in each group will be estimated using the cumulative incidence function with death as competing event and compared by a risk difference with 95% CI. The competing events 30-day deaths with and without previous readmission will also be estimated using the cumulative incidence function and compared by a risk difference with 95% CI. Count outcomes (number of days of hospitalization, number of emergency room visits and primary care provider visits) will be analyzed by Poisson or negative binomial regression with the time of observation as an offset. Effects will be reported as incidence rate ratios with 95% CI. Time to readmission or death (whatever comes first) will be analyzed by Cox regression and reported as hazard ratio with 95% CI.

11.4.3 Secondary Analyses

For all outcomes a secondary analysis will be done on the per-protocol set, i.e. all patients that received the pre-discharge component plus at least one of the 2 post-discharge follow-up phone calls of the intervention and completed the study according to protocol.

In a sensitivity analysis for all outcomes, all patients with readmissions within the first 24 hours will be excluded.

Furthermore, for the primary outcome subgroups defined by 1) risk for readmission (HOSPITAL score of 5 and 6 vs ≥7), and whether the patient suffers from 2) diabetes, 3) chronic heart failure, or 4) COPD will be analyzed using logistic regression with treatment group, subgroup indicator and their interaction as covariates. An odds ratio with 95% confidence interval will be reported for each subgroup together with a p-value for interaction.

11.4.4 Interim analyses

There is no interim analysis planned.

11.4.5 Safety analysis

Since the primary outcome is related to the SAE we will not run any specific analysis.

11.4.6 Deviation(s) from the original statistical plan

The SAP is a version-controlled document which allows to tracking of all changes to the statistical analysis. Any post-hoc changes will be transparently reported in all publications.

11.5 Handling of missing data and drop-outs

Missing data may occur due to drop-outs or deaths. The former will lead the absence of all outcome information as outcomes are only assessed once and will be accounted for by multiple imputation based on all available baseline information. The latter will not lead to missing data for the primary outcome, 30-day deaths and time to primary outcome. For 30-day unplanned readmission, death is a competing event and the cumulative incidence at 30 days will be used. Count outcomes will be handled by including the observation time as offset in the Poisson regression. For patient satisfaction and medication adherence multiple imputations will be used. In a sensitivity analysis, the worst possible outcome will be assigned to deaths (i.e. low adherence with MMAS-4 score of 4 and a negative respond to all question of the CTM-3).

12. QUALITY ASSURANCE AND CONTROL

12.1 Data handling and record keeping / archiving

The Investigators will maintain appropriate medical and research records for this trial, in compliance regulatory and institutional requirements for the protection of confidentiality of subjects. The Principal Investigator, Sub-Investigator, and Clinical Research Nurses or Coordinators will have access to the records.

The Principal Investigators will permit authorized representatives of the Co-Sponsor and regulatory agencies to examine (and when required by applicable law, to copy) clinical records for the purposes of quality assurance reviews, audits, and evaluation of the study safety and progress.

12.1.1 Case Report Forms

For each enrolled trial participant, an eCRF will be maintained. All data will be entered in the eCRF at the local site. eCRFs must be kept current to reflect subject status at each phase during the course of the trial and are part of the central acquisition by the CTU Bern. Coded identification for each patient will be as follow: [trial-ID]-[site-no]-consecutive number. Authorized to enter data into the eCRF are the local trial team staff according to the authorization list. Authorized persons will be identified by their usernames. The local PI is responsible for proper training and instruction of the trial personnel filling data into the eCRF.

12.1.2 Specification of source documents

Source data must be available at the site to document the existence of the trial participants. The following documents are source documents and can only be found at the local trial site:

- Demographics and administrative data
- completed questionnaires during hospital stay as well as follow-up
- Informed consent form
- Patient screening, enrolment and randomization log
- Patient records and medical history including reports from treating physician
- Laboratory reports
- Documentation of the patients medical treatment
- Nurse report including autonomy level of the patient if available.
- Documented telephone interview for CTM-3 and MMAS-4.
- Results of relevant examinations
- Any other relevant record to document SAEs

12.1.3 Record keeping / archiving

All trial data must be archived at the participating site for a minimum of 10 years or according to national requirements after trial termination or premature termination of the clinical trial. In addition, the electronic data will be kept for the mandatory period.

12.2 Data management

Describe plans for data entry, coding, security, and storage, including any related processes to promote data quality (e.g., double data entry; range checks for data values). In case electronic data

capture systems are used, this chapter shall include a description of procedures for verification, validation and securing the database.

Reference to where details of data management procedures can be found, if not in the protocol.

12.2.1 Data Management System

The CRFs in this trial are implemented electronically using a dedicated electronic data capturing (EDC) system (REDCap, https://www.project-redcap.org/). The EDC system is activated for the trial only after successfully passing a formal test procedure. All data entered in the CRFs are stored on a Linux server in a dedicated mySQL database.

Responsibility for hosting the EDC system and the database lies with CTU Bern.

12.2.2 Data security, access and back-up

The server hosting the EDC system and the database is kept in a locked server-room. Only the system administrators have direct access to the server and back-up tapes. A role concept with personal passwords (site investigator, statistician, monitor, administrator etc.) regulates permission for each user to use the system and database as he/she requires.

All data entered into the CRFs are transferred to the database using Secure Sockets Layer (SSL) encryption. Each data point has attributes attached to it identifying the user who entered it with the exact time and date. Retrospective alterations of data in the database are recorded in an audit table. Time, table, data field and altered value, and the person are recorded (audit trail).

A multi-level back-up system is implemented. Back-ups of the whole system including the database are run internally several times per day and on external tapes once a day. The back-up tapes are stored in a secure place in a different building.

The personally identifiable information (contact information of the participants) will be stored separately from all other study variables. Contact information are necessary in order to contact the participants randomized into the intervention group for the follow-up phone calls at day 3 and day 14 (visit 2 and 3), as well as for the outcome collection at day 30 (visit 4). While the study variables are stored in Redcap, the contact info will be stored in an Excel spreadsheet in a different secured cloud storage (htpps://docs.zoho.eu). The login access to the cloud is nominative and protected by a personal password. The access to the contact information will be strictly restricted only to the study nurses who need to contact the participants for the visit 2, 3, and 4. The cloud allows versioning, and audit trails to identify which change has been made by which study nurse. Only the necessary contact information will be collected at time of inclusion, such as the name and phone numbers of the participants as well as their proxy, the name and phone number of their primary care physician. No medical information will be stored in the contact information spreadsheet, since they are exclusively stored in Redcap. At the end of the study, once the outcomes have been collected for all included participants, the contact information file will be permanently deleted.

12.2.3 Analysis and archiving

Data files will be extracted from the database into statistical packages to be analyzed. The status of the database at this time is recorded in special archive tables.

The study database with all archive tables will be securely stored by CTU Bern for at least 10 years. The sponsor also keeps the Trial Master File and final reports for at least 10 years.

12.2.4 Electronic and central data validation

Data is checked by the EDC system for completeness and plausibility. Furthermore, selected data points are cross-checked for plausibility with previously entered data for that participant. If applicable: In addition, central data reviews will be performed on a regular basis to ensure completeness of the data

collected and accuracy of the primary outcome data.

Before database lock the PI will validate the collected data with his signature.

Coding/Pseudonymization

Study-related data of the patient will be collected in a coded manner. A code (unique, consecutive numbered per center) will be attributed to each patient registered.

The name, address, and contact information will be collected in order to reach the patient for the 2 follow-up calls that are part of the intervention, and for the phone interview at 30 days to collect the outcomes. The 2 follow-up phone calls at day 3 and 14 (part of the intervention) will be performed by the local study nurse from the enrollment site. The phone call at day 30 for outcome collection purposes will be performed by a blinded study nurse from one of the other study site. All identified data (first name, last name, home address, phone number, name of next of kin, phone number of next of kin, name and contact information of the primary care physician) will be collected at inclusion into a file outside the Redcap CRF with very limited access (only the study nurse in charge of the intervention at each participating site will have access, and the blinded study nurse in charge of the outcome collection). This file will be stored in a secured cloud storage as specified under pint 12.2.2. The blinded study nurse for outcome collection will have a restricted access in Redcap only to the to the outcome data collection form and end of study form. In that way, the study nurse will not be able to be aware of the group allocation of the participants.

12.3 Monitoring

For quality control of study conduct and data retrieval, at all participating sites, remote and on-site monitoring visits will be performed during study conduct by an appropriately trained and qualified monitor of CTU Bern. The focus of monitoring will be on the implemented study processes, including the informed consent procedure, patient eligibility, primary endpoint, and on study documentation (essential documents). Any findings and comments will be documented in site visit report and communicated to the sponsor as applicable. Prior to study start (first participant enrolled) a monitoring plan detailing all monitoring-related procedures, including time points of visits, will be developed. Study site personnel will support the monitor in his/her activities. All source data and relevant documents will be accessible to monitors, and questions of monitors are answered during site visits.

In addition to on-site monitoring, central data monitoring will be performed to ensure completeness and consistency of the data in the eCRF.

12.4 Audits and Inspections

Source data/documents must be available to audits by the Co-Sponsor, the Coordinating PI or designee or to inspections by competent authorities.

A competent authority or CEC may wish to conduct an inspection (during the study or after its completion). If an inspection is requested, the local PI must inform the Coordinating PI and the local Co-sponsor immediately. The Investigators at the participating sites will give full cooperation to the inspectors in their activities.

The Coordinating PI may exclude participating sites/Investigators from further participation in the study because of fraud or non-compliance with the study protocol, ICH-GCP guidelines, or applicable laws.

Study sites or PIs may stop recruiting patients to this study when the Investigator or the Co-sponsor deems inclusion of patients into this study to be no longer ethical for medical or organizational reasons. In this case, the PI should give detailed reasons to the Coordinating PI.

12.5 Confidentiality, Data Protection

Trial-related data of the patient will be collected in a coded manner. A unique patient identification

number will be attributed to each patient registered into the trial (for more details see section 12.1.1). Identification of patients must be guaranteed at each site using the patient screening, enrolment and identification list. In order to avoid identification errors, patients identification number and the year of birth have to be provided on the eCRF. Patient confidentiality will be maintained according to applicable legislation.

Direct access to source documents will be permitted for purposes of monitoring, audits and inspections.
The monitoring institution (i.e. CTU Bern) and the local authorities will have access to all information necessary for such tasks during and after the study.

12.6 Storage of biological material and related health data

All health related data will be stored at the local recruiting site at a secure location.

13. PUBLICATION AND DISSEMINATION POLICY

We plan to stimulate the implementation of our study results in a variety of ways, both locally in Switzerland and internationally:

1. We plan to disseminate our findings by publishing our study results in peer-reviewed medical journals, but also by presenting our study at national and international meetings (annual meetings of the Swiss Society of General Internal Medicine, of the Society of Hospital Medicine, of the Society of General Internal Medicine, and/or the International Society for Quality in Health Care).
2. We plan to disseminate the results through media press releases, that includes newspaper articles and radio interviews. This will insure to reach a large audience, including the patients themselves.
3. We will create a « TARGET-READ toolkit » to provide to the hospital all required information in order to replicate the discharge intervention used in our study. The packet will contain a guide that takes users step-by-step through the process of implementing and refining change processes at their own institutions, but also all patient's documents developed for the TARGET-READ trial. This toolkit will be made available online on the study website.
4. We will get the support from the ANQ (National Association for the development of Quality in Hospitals and Clinics in Switzerland). This association is founded by all Swiss hospitals and clinics in order to promote the quality of care. If shown successful, the ANQ is willing to help disseminate the TARGET-READ transition of care intervention to all hospitals and clinics in Switzerland, and also support the dissemination of the TARGET-READ toolkit.
5. We will work with the Agency for Healthcare Research and Quality (AHRQ) and the AHRQ Office of Communication and Knowledge Transfer to disseminate our findings through their Innovations Exchange platform. This platform includes high quality study results that are believed to change the clinical practice (https://innovations.ahrq.gov). The AHRQ contacted me after the publication of the development of the HOSPITAL score, and were willing to include the HOSPITAL score as soon as it would have been tested along with an intervention (the TARGET-READ trial fit to this criterion). The AHRQ Innovation Exchange offers an excellent opportunity to disseminate largely and internationally our study results.

Together, these activities will ensure that we share with many stakeholders what we learn from this study, and therefore may improve the quality of transition of care, and reduce complications in the post-hospital discharge period.

14. FUNDING AND SUPPORT

14.1 Funding

This project is funded entirely by a SNSF-professorship from the Swiss National Science Foundation (PP00P3_170656). The funders reviewed and approved the project, but have no role in the study design, interpretation of the data, or manuscript writing.

There is no conflict of interest to declare.

14.2 Other Support

No other support.

15. INSURANCE

This research project is of risk category A with minimal risks and burdens and is therefore exempt of insurance requirement.

16. REFERENCES

1. Office SFS. Statistique médicale des hôpitaux 2014. http://wwwbfsadminch/bfs/portal/de/index/news/publikationenhtml?publicationID=6827. 2014:[accessed on 22.01.2016].

2. Medicare Hospital Quality Chartbook 2012;www.cms.gov/Medicare/Quality-Initiatives-Patient-Assessment-Instruments/HospitalQualityInits/Downloads/MedicareHospitalQualityChartbook2012.pdf (accessed on January 6, 2016).

3. Krumholz HM. Post-hospital syndrome--an acquired, transient condition of generalized risk. *N Engl J Med*. 2013;368:100-2.

4. Jencks SF, Williams MV and Coleman EA. Rehospitalizations among patients in the Medicare fee-for-service program. *NEnglJMed*. 2009;360:1418-1428.

5. Forster AJ, Clark HD, Menard A, Dupuis N, Chernish R, Chandok N, Khan A and van Walraven C. Adverse events among medical patients after discharge from hospital. *CMAJ*. 2004;170:345-9.

6. Forster AJ, Murff HJ, Peterson JF, Gandhi TK and Bates DW. The incidence and severity of adverse events affecting patients after discharge from the hospital. *AnnInternMed*. 2003;138:161-167.

7. (PPACA) PPaACA. Public Law 111–148. 2010.

8. The Federal Council’s health-policy priorities - Health2020. 01.2013.

9. van Walraven C, Bennett C, Jennings A, Austin PC and Forster AJ. Proportion of hospital readmissions deemed avoidable: a systematic review. *CMAJ*. 2011;183:E391-402.

10. Halfon P, Eggli Y, van MG, Chevalier J, Wasserfallen JB and Burnand B. Measuring potentially avoidable hospital readmissions. *JClinEpidemiol*. 2002;55:573-587.

11. Melton LD, Foreman C, Scott E, McGinnis M and Cousins M. Prioritized post-discharge telephonic outreach reduces hospital readmissions for select high-risk patients. *Am J Manag Care*. 2012;18:838-44.

12. Naylor MD, Brooten DA, Campbell RL, Maislin G, McCauley KM and Schwartz JS. Transitional care of older adults hospitalized with heart failure: a randomized, controlled trial. *J Am Geriatr Soc*. 2004;52:675-84.

13. Hansen FR, Poulsen H and Sorensen KH. A model of regular geriatric follow-up by home visits to selected patients discharged from a geriatric ward: a randomized controlled trial. *Aging (Milano)*. 1995;7:202-6.

14. Dudas V, Bookwalter T, Kerr KM and Pantilat SZ. The impact of follow-up telephone calls to patients after hospitalization. *Am J Med*. 2001;111:26S-30S.

15. Leppin AL, Gionfriddo MR, Kessler M, Brito JP, Mair FS, Gallacher K, Wang Z, Erwin PJ, Sylvester T, Boehmer K, Ting HH, Murad MH, Shippee ND and Montori VM. Preventing 30-day hospital readmissions: a systematic review and meta-analysis of randomized trials. *JAMA Intern Med*. 2014;174:1095-107.

16. Hasan O, Meltzer DO, Shaykevich SA, Bell CM, Kaboli PJ, Auerbach AD, Wetterneck TB, Arora VM, Zhang J and Schnipper JL. Hospital readmission in general medicine patients: a prediction model. *JGenInternMed*. 2010;25:211-219.

17. van Walraven C, Dhalla IA, Bell C, Etchells E, Stiell IG, Zarnke K, Austin PC and Forster AJ. Derivation and validation of an index to predict early death or unplanned readmission after discharge from hospital to the community. *CMAJ*. 2010;182:551-557.

18. Dhalla IA, O'Brien T, Morra D, Thorpe KE, Wong BM, Mehta R, Frost DW, Abrams H, Ko F, Van Rooyen P, Bell CM, Gruneir A, Lewis GH, Daub S, Anderson GM, Hawker GA, Rochon PA and Laupacis A. Effect of a postdischarge virtual ward on readmission or death for high-risk patients: a randomized clinical trial. *JAMA*. 2014;312:1305-12.

19. Allaudeen N, Schnipper JL, Orav EJ, Wachter RM and Vidyarthi AR. Inability of providers to predict unplanned readmissions. *J Gen Intern Med*. 2011;26:771-6.

20. Kansagara D, Englander H, Salanitro A, Kagen D, Theobald C, Freeman M and Kripalani S. Risk prediction models for hospital readmission: a systematic review. *JAMA*. 2011;306:1688-98.

21. Donze J, Aujesky D, Williams D and Schnipper JL. Potentially avoidable 30-day hospital readmissions in medical patients: derivation and validation of a prediction model. *JAMA internal medicine*. 2013;173:632-8.

22. Donzé J, Williams MV, Robinson EJ, Zimlichman E, Aujesky DA, Vasilevskis EE, Kripalini S, Metlay JP, Wallington T, Fletcher GS, Auerbach AD and Schnipper JL. International Validity of the "HOSPITAL" Score to Predict 30-day Potentially Avoidable Readmissions in Medical Patients *JAMA Internal Medicine*. 2016;[in press].

23. Donzé J, Stirnemann J, Marques-Vidal P and Aujesky D. HOSPITAL score predicts patients at high-risk of potentially avoidable readmission: multicenter validation study in Switzerland. *[manuscript in preparation]*.

24. Aubert CE, Folly A, Mancinetti M, Hayoz D and Donzé J. Prospective Validation of the “Hospital” Score to Predict Patients at High Risk of Unplanned Readmission. *Swiss Medical Weekly*. 2016.

25. Burke RE, Williams MV, Robinson EJ, Vasilevskis EE, Kripalani S, Metlay JP, Fletcher GS, Auerbach AD, Schnipper JL and Donze JD. The HOSPITAL score predicts potentially avoidable readmissions in conditions targeted by the Readmissions Reduction Program. *Medical Care*. 2016;[accepted].

26. Aubert CE, Schnipper JL, Williams MV, Robinson EJ, Zimlichman E, Vasilevskis EE, Kripalani S, Metlay JP, Wallington T, Fletcher GS, Auerbach AD, Aujesky D and Donze JD. Simplification of the HOSPITAL score for predicting 30-day readmissions. *[under review at Journal of General Internal Medicine]*.

27. Justice AC, Covinsky KE and Berlin JA. Assessing the generalizability of prognostic information. *Ann Intern Med*. 1999;130:515-24.

28. Cooksley T, Nanayakkara PW, Nickel CH, Subbe CP, Kellett J, Kidney R, Merten H, Van Galen L, Henriksen DP, Lassen AT, Brabrand M and safer@home c. Readmissions of medical patients: an external validation of two existing prediction scores. *QJM*. 2015.

29. Donze J, Rodondi N, Waeber G, Monney P, Cornuz J and Aujesky D. Scores to predict major bleeding risk during oral anticoagulation therapy: a prospective validation study. *Am J Med*. 2012;125:1095-102.

30. Zhu W, He W, Guo L, Wang X and Hong K. The HAS-BLED Score for Predicting Major Bleeding Risk in Anticoagulated Patients With Atrial Fibrillation: A Systematic Review and Meta-analysis. *Clin Cardiol*. 2015;38:555-61.

31. McGinn TG, Guyatt GH, Wyer PC, Naylor CD, Stiell IG and Richardson WS. Users' guides to the medical literature: XXII: how to use articles about clinical decision rules. Evidence-Based Medicine Working Group. *JAMA*. 2000;284:79-84.

32. Statistique médicale des hôpitaux 2014. 3.2016.

33. van Walraven C, Wong J and Forster AJ. Influence of neighborhood household income on early death or urgent hospital readmission. *J Hosp Med*. 2013;8:261-6.

34. McIlvennan CK and Allen LA. Outcomes in acute heart failure: 30-day readmission versus death. *Curr Heart Fail Rep*. 2014;11:445-52.

35. Lau D, Padwal RS, Majumdar SR, Pederson JL, Belga S, Kahlon S, Fradette M, Boyko D and McAlister FA. Patient-Reported Discharge Readiness and 30-Day Risk of Readmission or Death: A Prospective Cohort Study. *Am J Med*. 2016;129:89-95.

36. Morisky DE, Green LW and Levine DM. Concurrent and predictive validity of a self-reported measure of medication adherence. *Med Care*. 1986;24:67-74.

37. Morisky DE, Malotte CK, Choi P, Davidson P, Rigler S, Sugland B and Langer M. A patient education program to improve adherence rates with antituberculosis drug regimens. *Health Educ Q*. 1990;17:253-67.

38. Morisky DE, Ang A, Krousel-Wood M and Ward HJ. Predictive validity of a medication adherence measure in an outpatient setting. *J Clin Hypertens (Greenwich)*. 2008;10:348-54.

39. Parry C, Mahoney E, Chalmers SA and Coleman EA. Assessing the quality of transitional care: further applications of the care transitions measure. *Med Care*. 2008;46:317-22.

40. Schulz KF, Altman DG, Moher D and Group C. CONSORT 2010 statement: updated guidelines for reporting parallel group randomized trials. *Ann Intern Med*. 2010;152:726-32.

41. Hoffmann TC, Glasziou PP, Boutron I, Milne R, Perera R, Moher D, Altman DG, Barbour V, Macdonald H, Johnston M, Lamb SE, Dixon-Woods M, McCulloch P, Wyatt JC, Chan AW and Michie

S. Better reporting of interventions: template for intervention description and replication (TIDieR) checklist and guide. *BMJ*. 2014;348:g1687.

17. APPENDICES

Not applicable.

b
UNIVERSITÄT BERN

Faculty of Medicine
CTU Bern

Transition cAre inteRvention tarGeted to high-risk patiEnts To Reduce rEADmission (TARGET-READ): A randomized controlled trial

Analysis report

Version 3.0 — January 11, 2023

Dr. Lukas Bütikofer
Senior Statistician
CTU Bern
Mittelstrasse 43
CH-3012 Bern
lukas.buetikofer@ctu.unibe.ch
www.ctu.unibe.ch

Contents

1 Methods **3**

1.1 Overview . . . 3

1.2 Outcomes . . . 3

1.3 Patients sets . . . 3

1.4 Analysis . . . 4

1.4.1 Primary analysis . . . 4

1.4.2 Secondary analysis . . . 5

1.4.3 Sensitivity analysis . . . 5

1.4.4 Subgroup analysis . . . 6

1.5 Missing data . . . 6

1.6 Quality controls . . . 7

1.7 Changes from SAP . . . 7

2 Patient flow **8**

3 Protocol deviations and patient sets **9**

4 Baseline patient characteristics **10**

5 Procedural characteristics **13**

6 Primary analyses **18**

6.1 Summary . . . 23

7 Secondary analyses **24**

7.1 Summary . . . 27

8 Sensitivity analyses **28**

8.1 Summary . . . 31

9 Subgroup analyses **32**

9.1 Summary . . . 33

Appendix **34**

Bibliography **36**

1 Methods

1.1 Overview

- This analysis is based on study protocol version 1.1 (13.02.2018) and statistical analysis plan (SAP) version 2.0 (22.04.2020).
- Data was exported and frozen on August 05, 2020.
- The report was generated on January 11, 2023.

1.2 Outcomes

The primary outcome is the number of patients who have an unplanned readmission or die within 30 days after discharge.

Secondary outcomes are:

- Number of deaths within 30 days after discharge
- Number of patients with unplanned readmission within 30 days after discharge
- Time to first unplanned readmission or death within 30 days
- Main cause of readmission or death
- Post-discharge health care utilization within 30 days after discharge from index hospitalization:
  - Number of unplanned hospital readmissions
  - Number of planned hospital readmissions
  - Total number of unplanned days of hospitalizations
  - Total number of planned days of hospitalizations
  - Number of emergency room visits
  - Number primary care provider visits
- Patient's perspective (satisfaction) on quality of transition of care between hospital and home assessed by the three-item care transition measure (CTM-3) at 30 days.
- Costs of readmission at 30 days.

1.3 Patients sets

The full analysis set (FAS) includes all randomized subjects who were discharged alive. Following the intent-to-treat (ITT) principle, subjects will be analyzed according to the treatment they are assigned to at randomization regardless of the treatment actually received.

The per-protocol set (PPS) consists of all subjects in the FAS who received the allocated treatment and did not have any major protocol deviations, i.e.

- Violation of inclusion or exclusion criteria
- No informed consent signed
- Cross-overs, i.e. control patients receiving any part of the intervention
- Patients not discharged home or to nursing home
- HOSPITAL score risk group used for stratification does not match the HISPOTAL score at discharge
- Not receiving post-discharge component of the intervention and at least one of the follow-up phone calls (unless the patient was readmitted or died before the follow-up phone call).
- Blindness breach

1.4 Analysis

- Statistical methods are described in detail in the SAP.
- All analyses were done in Stata version 15.1 [1], plots were done in R version 4.0.3 (2020-10-10) [2].
- Continuous variables are presented with mean and standard deviation (sd), or median, lower quartile (lq) and upper quartile (uq).
- Categorical variables are presented with number (n) and percentage (%) of patients.
- If there are missing data, the number of non-missing observations is shown.
- For all outcomes, only events within 30 days were considered.
- Unless explicitly mentioned, all analyses were adjusted for the stratification factors used in randomization, i.e. discharge site and readmission risk (intermediate risk with a simplified HOSPITAL score of 4 or 5 vs high risk with a simplified HOSPITAL score of 6 or higher).

1.4.1 Primary analysis

- Primary analysis was based on the FAS.
- The primary outcome and the binary secondary outcomes deaths within 30 days and patient satisfaction were analyzed using a Mantel-Haenszel risk difference stratified for the stratification factors used in randomization (discharge site and readmission risk, i.e. simplified HOSPITAL score of 4 or 5 vs 6 or higher) with a Klingenberg two-sided 95% confidence interval (CI) [3] and a stratified Cochran-Mantel-Haenszel test (using emh in Stata).
- The risk of 30 day unplanned readmissions in each group was estimated using the cumulative incidence function with death as competing event calculated from flexible parametric survival models (using stpm2 followed by standsurv in Stata) [4, 5]. Groups were compared using the cumulative incidence difference with 95% CI and a z-test, based on delta method standard errors and a normal approximation. We also report the cumulative incidence of the competing event (death without prior readmission) for each group and the risk difference between groups.

- Time to unplanned readmission or death was compared between groups using a log-rank test stratified for the stratification factors used in randomization. As an effect measure, we estimated the restricted mean survival time truncated at 30 days using flexible parametric survival models with group and sratification factors as covariates (using stpm2 followed by standsurv in Stata) [6]. We report the restricted mean survival time for each group and the difference between groups with 95% CI and a p-value.
- Count outcomes (number of unplanned and planned hospital readmissions, number of unplanned and planned days of hospitalization, number of emergency room visits, number of primary care provider visits) are presented with number of patients, person-time and incidence per 30 person-days with 95% CI. Groups were compared using a negative binomial regression with robust standard errors (using nbreg with vce(robust) in Stata) with the group and the stratification factors as covariates and the observation time as offset. There was considerable zero inflation for the number of unplanned days of hospitalization and it was modeled using zero-inflated negative binomial regression with robust standard errors (using zinb with option vce(robust) in Stata). We report incidence rate ratio with 95% CI.
- The costs of readmission was only analyzed for patients who had a readmission using linear regression with the treatment group and the stratification factors used in randomization as covariates. The treatment effect is presented as mean difference with 95% CI and a p-value. Since the cost data were skewed we used robust standard errors and included a further sensitivity analysis based on a generelaized linear model with a gamma distribution and a log link. The results is presented as mean ratio with 95% CI and a p-value.
- For the primary outcome and the risk of unplanned readmission we also report stratum-specific estimates because there was evidence that the effect of the intervention was not homogeneous over the strata.

1.4.2 Secondary analysis

The secondary analysis for all outcomes was based on the per-protocol set (PPS) only considering patients without missing value for the respective outcome (complete cases).

1.4.3 Sensitivity analysis

We included five sensitivity analyses:

- The primary endpoint was re-analyzed excluding early readmissions or death, i.e. patients who died or had a readmission up to the day after index discharge
- All outcomes were analyzed in crude analyses not adjusting for stratification factors. Binary outcomes were be compared by chis-squared test, continuous outcomes by Student's t-test or the Wilcoxon-Mann-Whitney test, count outcomes using an exact Poisson-test and time-to-event outcomes by a log-rank test. Effects are presented as non-stratified risk difference, mean difference or Mann-Whitney statistic (i.e. the probability that a random patient from the intervention group will have a higher value than a random patient from the control group), incidence rate ratio, and restricted mean survival time difference, respectively, with 95% CI.
- The primary outcome was also estimated using survival methods, i.e.
  - a flexible parametric survival model with the group and stratification factors as covariates and
  - the Kaplan-Meier-estimator ignoring the stratification.

- Unplanned readmission at 30 days in the presence of the competing risk of death was also estimated using the non-parametric cumulative incidence function estimator with 95% CI according to [7] ignoring the stratification.
- Missing data for patient satisfaction due to death were imputed assuming the worst case, i.e. a negative respond to all question of the CTM-3.

1.4.4 Subgroup analysis

The primary outcome was analyzed for the following subgroups:

- Risk for readmission (HOSPITAL score of 4 or 5 vs =6)
- Clinical site
- Diabetes
- Chronic heart failure
- COPD
- Cancer
- Living place (nursing home vs rest)
- Living status (alone vs rest)
- Health insurance (Semi-private and private vs rest)

Subgroups were analyzed using a generalized linear model with a binomial distribution and an identity link. Effects for each subgroup were calculated from separate models. An interaction was analyzed comparing models with treatment group, subgroup and stratification factor with and without the interaction of treatment and subgroup using a likelihood ratio test.

As a secondary analysis, subgroups were analyzed using Mantel-Haenszel methodology. A Mantel-Haenszel risk difference stratified for the stratification factor used at randomization was calculated for each subgroup with a two-sided 95% CI. An interaction was tested for using a Mantel-Haenszel test of homogeneity for the subgroup ignoring the stratification factors used at randomization.

1.5 Missing data

- The number of patients with non-missing observations is reported for each outcome
- Drop outs were rare (less than 5%) and were
  - assumed to have no readmission or death,
  - censored for survival analyses,
  - assumed to have no event and an offset of 1 hour for count outcomes,
  - multiply imputed for patient satisfaction (see below).
- Deaths did not lead to missing data for the primary outcome, 30-day deaths and time to primary outcome. For death or readmission at the day of discharge, we used an event time of half a day.
- For the other outcomes deaths were:

  - treated as competing event for 30-day unplanned readmission via the cumulative incidence.
  - included using the time of death as offset for count outcomes
  - multiply imputed for patient satisfaction (see below).
- Multiple imputation for patient satisfaction were based on age, gender, nationality, living status, discharged destination, insurance, duration of index hospitalization, HOPSITAL score variables, death, readmission, number of PCP visits, source of revenue and the treatment group. Fifty imputed datasets were calculated and combined using Rubin's rule [8].
- The cost of readmission was only analyzed for patients with a documented readmission. Missing data was multiply imputed in a separate model with only those patients using the dame variables as for patient satisfaction (except for readmission).

1.6 Quality controls

- A second statistician reprogrammed the primary and secondary analysis of the primary outcome based on the exported data.
- The same results were obtained.

1.7 Changes from SAP

The analysis is consistent with principle features of the statistical methods described in the statistical analysis plan (SAP). The following deviations were made:

- For the primary outcome and the risk of unplanned readmission, we also report stratum-specific estimates because there was some evidence that the effect of the intervention may not be homogeneous over the strata.
- For the analysis of count variables, we used robust standard errors for the negative binomial regression.
- For the analysis of the number of unplanned hospitalization days, we used zero-inflated negative binomial regression since there was evidence of zero-inflation.
- The cause of readmission or death is not yet included because it was only available as freetext and requires categorization.
- For the analysis of the cost of readmission we used robust standard errors as the data was skewed. In addition, we included a sensitivity analysis based on a generalized linear model with a gamma distribution and a log link.

2 Patient flow

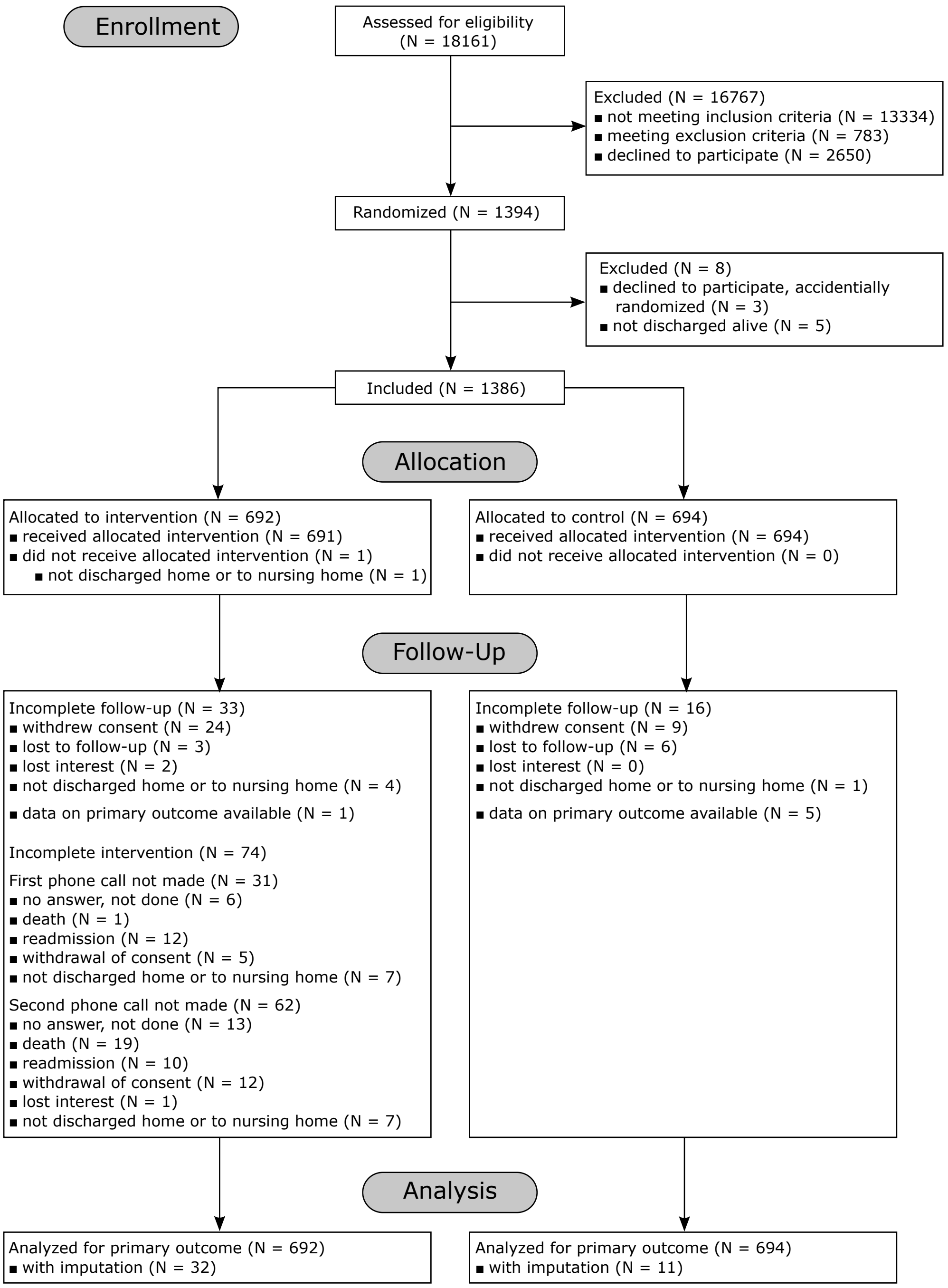

Figure 1: Patient flow.

3 Protocol deviations and patient sets

Table 1: Randomized patients included in the full analysis set (FAS) and the per-protocol set (PPS) and the reasons for exclusions.

	Total	Control group	Intervention group
Randomized	1394	698	696
Included in full analysis set (FAS)	1386/1394 (99%)	694/698 (99%)	692/696 (99%)
Reason for exclusion from FAS			
No consent	3/1394 (0.2%)	1/698 (0.1%)	2/696 (0.3%)
Not discharged alive	5/1394 (0.4%)	3/698 (0.4%)	2/696 (0.3%)
Included in per-protocol set (PPS), of those in FAS	1276/1386 (92%)	645/694 (93%)	631/692 (91%)
Reason for exclusion from PPS			
Not discharged home	29/1386 (2.1%)	14/694 (2.0%)	15/692 (2.2%)
Not at higher risk of readmission (HOSPITAL score < 4)	11/1386 (0.8%)	4/694 (0.6%)	7/692 (1.0%)
Incorrect hospital score risk group for stratification	63/1386 (4.5%)	31/694 (4.5%)	32/692 (4.6%)
Incomplete intervention	7/1386 (0.5%)	0/694 (0.0%)	7/692 (1.0%)

4 Baseline patient characteristics

Table 2: Sociodemographics.

	Total (N = 1386)	Intervention group (N = 692)	Control group (N = 694)
Age of the patient			
mean (sd)	72 (14)	71 (14)	72 (14)
median [lq, uq]	74 [64, 82]	74 [63, 82]	73 [65, 82]
Gender			
Male	712 (51%)	337 (49%)	375 (54%)
Female	674 (49%)	355 (51%)	319 (46%)
Nationality			
Switzerland	1182 (85%)	587 (85%)	595 (86%)
Germany	15 (1.1%)	11 (1.6%)	4 (0.6%)
France	27 (1.9%)	13 (1.9%)	14 (2.0%)
Italy	65 (4.7%)	29 (4.2%)	36 (5.2%)
Spain/Portugal	39 (2.8%)	21 (3.0%)	18 (2.6%)
East Europe	9 (0.6%)	3 (0.4%)	6 (0.9%)
Africa	20 (1.4%)	10 (1.4%)	10 (1.4%)
Other	29 (2.1%)	18 (2.6%)	11 (1.6%)
Living status			
With spouse/partner	682 (49%)	334 (48%)	348 (50%)
With another person	90 (6.5%)	55 (7.9%)	35 (5.0%)
Alone	614 (44%)	303 (44%)	311 (45%)
Living place type			
Home	1206 (87%)	590 (85%)	616 (89%)
Protected appartment	25 (1.8%)	11 (1.6%)	14 (2.0%)
Nursing home	145 (10%)	82 (12%)	63 (9.1%)
Other	10 (0.7%)	9 (1.3%)	1 (0.1%)
Nurse visits at home*			
No	667 (54%)	307 (50%)	360 (57%)
Yes	573 (46%)	302 (50%)	271 (43%)
Unknown	1 (0.1%)	1 (0.2%)	0 (0.0%)
Support at home for cleaning*	495 (40%)	246 (40%)	249 (39%)
Support at home to buy grocery*	211 (17%)	108 (18%)	103 (16%)
Support at home for eating*	218 (18%)	115 (19%)	103 (16%)
Source of revenue			
Employed	121 (8.7%)	60 (8.7%)	61 (8.8%)
Self-employed	34 (2.5%)	16 (2.3%)	18 (2.6%)
Unemployed	19 (1.4%)	12 (1.7%)	7 (1.0%)
Retired	1050 (76%)	517 (75%)	533 (77%)
Invalidity insurance	107 (7.7%)	56 (8.1%)	51 (7.3%)
Social	34 (2.5%)	17 (2.5%)	17 (2.4%)
Other	18 (1.3%)	12 (1.7%)	6 (0.9%)
Unknown	3 (0.2%)	2 (0.3%)	1 (0.1%)
Health insurance			
mean (sd)	1.8 (3.6)	1.9 (5.0)	1.7 (0.85)
median [lq, uq]	1.0 [1.0, 2.0]	1.0 [1.0, 2.0]	1.0 [1.0, 2.0]
missing	1 (0.1%)	1 (0.1%)	0 (0.0%)
Patient left against medical advice	14 (1.0%)	8 (1.2%)	6 (0.9%)

**Not applicable if living place type is nursing home*

Table 3: Index admission and HOSPITAL score.

	Total (N = 1386)	Intervention group (N = 692)	Control group (N = 694)
Patient discharged alive	1386 (100%)	692 (100%)	694 (100%)
Destination after discharge*			
Home	1207 (87%)	591 (85%)	616 (89%)
Nursing home	150 (11%)	86 (12%)	64 (9.2%)
Other acute care Hospital (including psychiatric Hospital).	2 (0.1%)	1 (0.1%)	1 (0.1%)
Rehab (general, cardiovascular, neuro, etc.)	8 (0.6%)	4 (0.6%)	4 (0.6%)
Palliative care	7 (0.5%)	4 (0.6%)	3 (0.4%)
Other	11 (0.8%)	6 (0.9%)	5 (0.7%)
Unknown	1 (0.1%)	0 (0.0%)	1 (0.1%)
Length of stay of index hospitalization (days)*			
mean (sd)	12 (7.2)	11 (6.9)	12 (7.5)
median [lq, uq]	10 [7.0, 14]	10 [7.0, 14]	10 [8.0, 13]
Costs (in CHF) of the index hospitalisation			
mean (sd)	15788 (14074)	15899 (14439)	15679 (13709)
median [lq, uq]	11792 [8243, 18229]	11792 [8264, 19125]	11817 [8115, 17629]
Last hemoglobin level available before discharge (g/l)			
mean (sd)	106 (17)	106 (17)	105 (17)
median [lq, uq]	105 [93, 116]	106 [94, 117]	105 [93, 116]
missing	2 (0.1%)	0 (0.0%)	2 (0.3%)
Last sodium level available before discharge (mmol/l)			
mean (sd)	138 (3.8)	138 (3.9)	138 (3.7)
median [lq, uq]	139 [136, 141]	139 [136, 141]	139 [136, 141]
missing	4 (0.3%)	2 (0.3%)	2 (0.3%)
Type of admission			
Elective (i.e. planned > 24 hours before hospital admission)	102 (7.4%)	43 (6.2%)	59 (8.5%)
Non elective	1284 (93%)	649 (94%)	635 (91%)
Number of hospitalization at the same hospital in the last 12 months			
mean (sd)	1.3 (1.4)	1.3 (1.4)	1.2 (1.4)
median [lq, uq]	1.0 [0.00, 2.0]	1.0 [0.00, 2.0]	1.0 [0.00, 2.0]
Active cancer defined as under treatment, or if remission < 5 years	596 (43%)	288 (42%)	308 (44%)
Hospital score (simplified)			
mean (sd)	5.1 (1.3)	5.0 (1.2)	5.1 (1.3)
median [lq, uq]	5.0 [4.0, 6.0]	5.0 [4.0, 6.0]	5.0 [4.0, 6.0]

**Only applicable if discharged alive*

Table 4: Index diagnosis.

	Total (N = 1386)	Intervention group (N = 692)	Control group (N = 694)
Main diagnosis category			
Heart failure	148 (11%)	74 (11%)	74 (11%)
Acute ischemic heart disease	29 (2.1%)	12 (1.7%)	17 (2.4%)
Arrhythmia	19 (1.4%)	10 (1.4%)	9 (1.3%)
Venous thromboembolism	29 (2.1%)	18 (2.6%)	11 (1.6%)
Stroke/TIA	25 (1.8%)	10 (1.4%)	15 (2.2%)
COPD exacerbation	51 (3.7%)	24 (3.5%)	27 (3.9%)
Pneumonia	113 (8.2%)	53 (7.7%)	60 (8.6%)
Other infection, sepsis	163 (12%)	79 (11%)	84 (12%)
Gastro-intestinal disorder	142 (10%)	78 (11%)	64 (9.2%)
Liver disorder	31 (2.2%)	18 (2.6%)	13 (1.9%)
Renal disorder	56 (4.0%)	27 (3.9%)	29 (4.2%)
Nutritional or metabolic disorder	51 (3.7%)	27 (3.9%)	24 (3.5%)
Adverse drug event	16 (1.2%)	13 (1.9%)	3 (0.4%)
Neoplasm	198 (14%)	92 (13%)	106 (15%)
Epilepsy	17 (1.2%)	12 (1.7%)	5 (0.7%)
Other	298 (22%)	145 (21%)	153 (22%)
Chronic heart failure	372 (27%)	194 (28%)	178 (26%)
Coronary disease	362 (26%)	184 (27%)	178 (26%)
Atrial fibrillation	294 (21%)	141 (20%)	153 (22%)
Peripheral artery disease	157 (11%)	79 (11%)	78 (11%)
Diabetes	384 (28%)	200 (29%)	184 (27%)
Dementia	32 (2.3%)	14 (2.0%)	18 (2.6%)
COPD	223 (16%)	105 (15%)	118 (17%)
Active cancer	594 (43%)	288 (42%)	306 (44%)
Chronic renal failure	399 (29%)	205 (30%)	194 (28%)
Liver cirrhosis	99 (7.1%)	46 (6.6%)	53 (7.6%)
Drug or alcohol abuse	197 (14%)	100 (14%)	97 (14%)
Epilepsy	60 (4.3%)	37 (5.3%)	23 (3.3%)
Any treated psychiatric disease	236 (17%)	118 (17%)	118 (17%)

5 Procedural characteristics

Table 5: Pre-discharge component of the intervention. Only for intervention group.

	Intervention group (N = 692)
Basic information about her/his main diseases has been performed?*	688 (99%)
Heart failure	196 (28%)
Coronary disease	184 (27%)
Peripheral artery disease	75 (11%)
Atrial fibrillation	133 (19%)
Thromboembolism	71 (10%)
Chronic obstructive lung disease	99 (14%)
Stroke	43 (6.2%)
Gastrointestinal bleeding	65 (9.4%)
Chronic renal failure	202 (29%)
Liver cirrhosis	48 (6.9%)
Diabetes	200 (29%)
Other	119 (17%)
No listed comorbidity	102 (15%)
Medication reconciliation has been performed?*	681 (98%)
Medication discrepency noticed?†	235 (34%)
Patient education about general health recommendation has been performed?*	690 (100%)
Dependence Level: score of the Katz Index*	
0	2 (0.3%)
1	9 (1.3%)
2	14 (2.0%)
3	23 (3.3%)
4	58 (8.4%)
5	129 (19%)
6	456 (66%)
Post discharge follow-up visit to the treating physician has been planned?*	596 (86%)
Discharge summary sent to the treating physician*	644 (93%)
Barriers to a safe discharge*	682 (99%)

**Missing for one patient*
†*Missing for 11 patients*

Table 6: Follow-up phone call at day 3. Answers only available for patients reached by phone.

	Intervention group (N = 692)
Patient reached by phone	661 (96%)
Reason why the patient wasn't reached	
No answer, not done	6 (19%)
Death	1 (3.2%)
Readmission	12 (39%)
Withdrawal of consent	5 (16%)
Lost to follow-up	0 (0.0%)
Lost interest	0 (0.0%)
Not discharged home or nursing	7 (23%)
Condition improved since discharge	
No, or rather no	60 (9.1%)
Yes, or rather yes	250 (38%)
Stable	298 (45%)
New symptom	53 (8.0%)
Since your discharge of the hospital, do you have more pain?	89 (13%)
How does the mobility/walking distance change since discharge?	
Mobility/walking distance improved	199 (30%)
Mobility/walking remained about the same	401 (61%)
Mobility/walking decreased	54 (8.2%)
Not able to evaluate	6 (0.9%)
missing	1 (0.2%)
Capacity to prepare meals alone?	401 (61%)
Weight trend over the last days/weeks*	
Increased	33 (10%)
Reduced	61 (18%)
Stable	170 (52%)
Unknown	50 (15%)
missing	16 (4.8%)
Glucose values most often†	
Between 4-10	115 (60%)
>10	17 (8.9%)
Between 10-15	8 (4.2%)
>15	3 (1.6%)
Unknown	44 (23%)
missing	5 (2.6%)
Do you take your medication as prescribed?	
No, or rather no	20 (3.0%)
Yes	640 (97%)
missing	1 (0.2%)
Is there any medication discrepancy between current list and list of discharge?	
No	466 (70%)
Yes	192 (29%)
missing	3 (0.5%)

continued on next page

Table 6: Follow-up phone call at day 3. Answers only available for patients reached by phone.

	Intervention group (N = 692)
Presence of any of those medications?	
Anticoagulants	205 (31%)
Narcotics and opiates	128 (19%)
Sedatives	162 (25%)
Insulin	97 (15%)
Did any of these Adverse Event occur since discharge?	
Dizziness	18 (2.7%)
Bleeding	3 (0.5%)
Hypoglycemia	1 (0.2%)
Delirium	0 (0.0%)
Lethargy / Oversedation	10 (1.5%)
Nausea / vomiting	21 (3.2%)
Fall	3 (0.5%)
None	409 (62%)
Patient had doctor's office visit since discharge	
No	305 (46%)
Yes as planned	278 (42%)
Yes as a new appointment	78 (12%)
Reason why the patient didn't have any treating physician visit since discharge	
Visit is planned	235 (77%)
Patient didn't want/ couldn't	10 (3.3%)
Treating physician couldn't/not available	20 (6.6%)
Other	37 (12%)
Unknown	3 (1.0%)
Suggestion to see the treating physician was necessary during this follow-up cal	206 (31%)
Patient education about her/his diseases has been refreshed	646 (98%)

**Only applicable if patient had Heart failure, Liver disorder, Chronic renal failure or Liver cirrhosis*
†*Only applicable if patient had diabetes*

Table 7: Follow-up phone call at day 14. Answers only available for patients reached by phone.

	Intervention group (N = 692)
Patient reached by phone	630 (91%)
Reason why the patient wasn't reached	
No answer, not done	13 (21%)
Death	19 (31%)
Readmission	10 (16%)
Withdrawal of consent	12 (19%)
Lost to follow-up	0 (0.0%)
Lost interest	1 (1.6%)
Not discharged home or nursing	7 (11%)
Condition improved since discharge	
No, or rather no	72 (11%)
Yes, or rather yes	295 (47%)
Stable	172 (27%)
New symptom	91 (14%)
Since your discharge of the hospital, do you have more pain?	113 (18%)
How does the mobility/walking distance change since discharge?	
Mobility/walking distance improved	291 (46%)
Mobility/walking remained about the same	244 (39%)
Mobility/walking decreased	92 (15%)
Not able to evaluate	3 (0.5%)
Capacity to prepare meals alone?	
No	228 (36%)
Yes	401 (64%)
missing	1 (0.2%)
Weight trend over the last days/weeks*	
Increased	69 (22%)
Reduced	75 (24%)
Stable	134 (43%)
Unknown	24 (7.7%)
missing	11 (3.5%)
Glucose values most often†	
Between 4-10	121 (67%)
>10	13 (7.2%)
Between 10-15	6 (3.3%)
>15	2 (1.1%)
Unknown	29 (16%)
missing	9 (5.0%)
Do you take your medication as prescribed?	
No, or rather no	19 (3.0%)
Yes	609 (97%)
missing	2 (0.3%)
Is there any medication discrepancy between current list and list of discharge?	
No	367 (58%)

continued on next page

Table 7: Follow-up phone call at day 14. Answers only available for patients reached by phone.

	Intervention group (N = 692)
Yes	259 (41%)
missing	4 (0.6%)
Presence of any of those medications?	
Anticoagulants	208 (33%)
Narcotics and opiates	124 (20%)
Sedatives	157 (25%)
Insulin	90 (14%)
Did any of these Adverse Event occur since discharge?	
Dizziness	23 (3.7%)
Bleeding	4 (0.6%)
Hypoglycemia	5 (0.8%)
Delirium	2 (0.3%)
Lethargy / Oversedation	8 (1.3%)
Nausea / vomiting	37 (5.9%)
Fall	8 (1.3%)
None	364 (58%)
Patient had doctor's office visit since discharge	
No	70 (11%)
Yes as planned	269 (43%)
Yes as a new appointment	289 (46%)
missing	2 (0.3%)
Reason why the patient didn't have any treating physician visit since discharge	
Visit is planned	19 (27%)
Patient didn't want/ couldn't	6 (8.6%)
Treating physician couldn't/not available	14 (20%)
Other	31 (44%)
Unknown	0 (0.0%)
Suggestion to see the treating physician was necessary during this follow-up cal	
No	444 (70%)
Yes	183 (29%)
missing	3 (0.5%)
Patient education about her/his diseases has been refreshed	
No	11 (1.7%)
Yes	617 (98%)
missing	2 (0.3%)

**Only applicable if patient had Heart failure, Liver disorder, Chronic renal failure or Liver cirrhosis*
†Only applicable if patient had diabetes

6 Primary analyses

Table 8: Primary analysis of the primary outcome (unplanned readmission or death within 30 days after discharge) and its components (deaths and readmissions within 30 days after discharge). Deaths without prior readmission is the competing event to unplanned readmission. A negative risk difference would indicate a benefit of the intervention.

	Intervention group (N = 692)	Control group (N = 694)	Risk difference (95% CI)	P-value	P-value for homogeneity
	n (%, 95% CI)				
Unplanned readmission or death	145 (21%, 18 to 24%)	134 (19%, 17 to 22%)	1.7% (-2.5 to 5.9%)	0.44	0.05
Death	32 (4.6%, 3.3 to 6.5%)	18 (2.6%, 1.6 to 4.1%)	2.0% (0.1 to 4.0%)	0.043	0.32
Unplanned readmission	127 (19%, 17 to 23%)	124 (18%, 15 to 21%)	1.3% (-2.9 to 5.4%)	0.55	0.06
Death without unplanned readmission	18 (2.8%, 1.8 to 4.3%)	10 (1.5%, 0.8 to 2.7%)	1.3% (-0.2 to 2.8%)	0.10	0.10

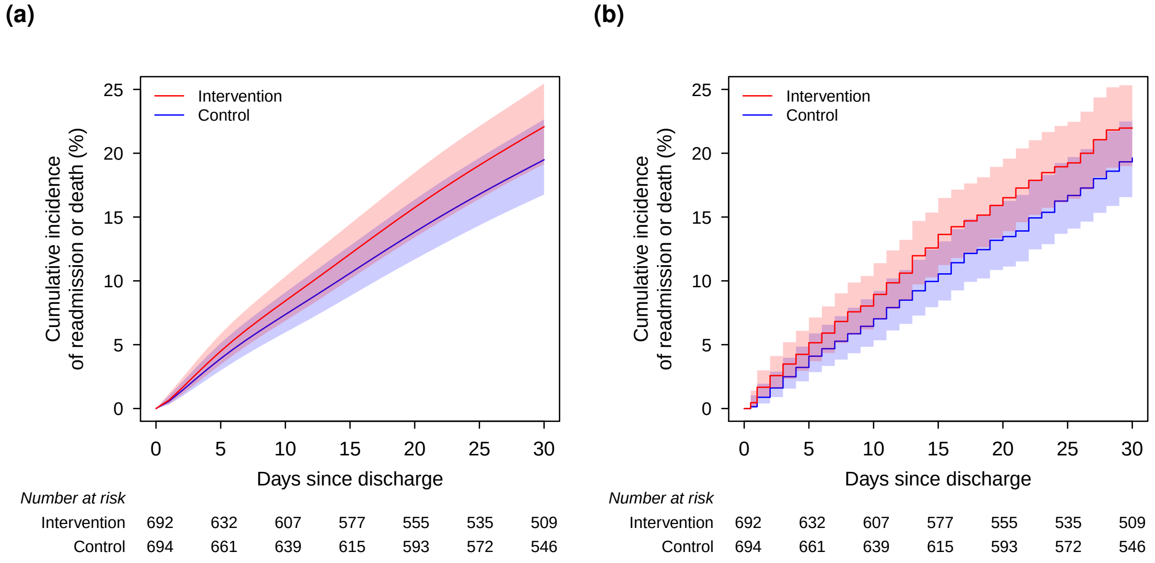

Figure 2: Cumulative incidence of the primary outcome (unplanned readmission or death within 30 days) estimated by a) flexible parametric survival models or b) the non-parametric estimator ignoring stratification.

(a) (b)

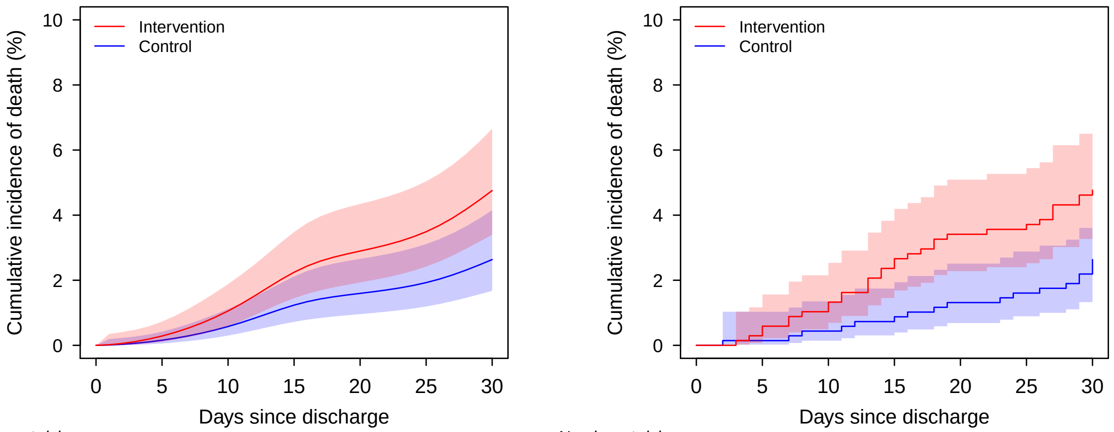

Number at risk							
Intervention	692	632	607	577	555	535	509
Control	694	661	639	615	593	572	546

Number at risk							
Intervention	692	675	670	653	642	641	628
Control	694	684	682	680	676	673	662

Figure 3: Cumulative incidence of death within 30 days estimated by a) flexible parametric survival models or b) the non-parametric estimator ignoring stratification.

(a) (b)

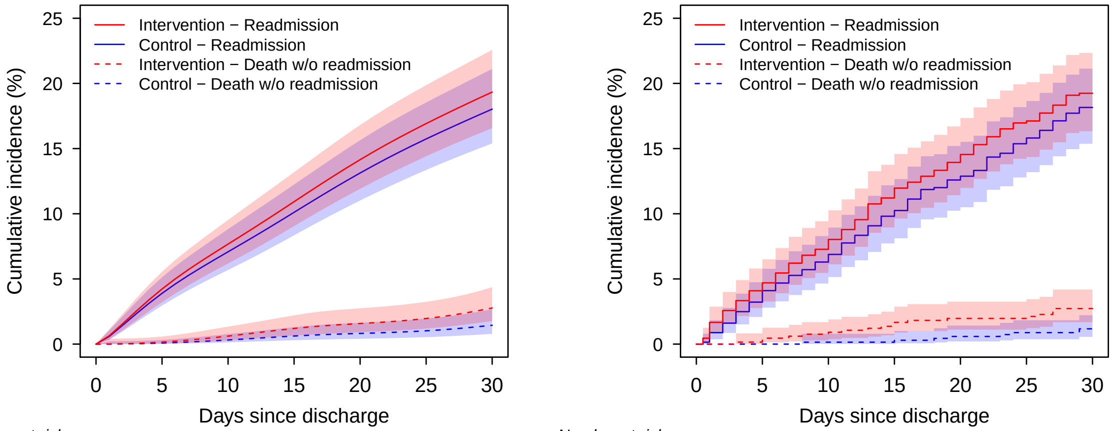

Number at risk							
Intervention	692	632	607	577	555	535	509
Control	694	661	639	615	593	572	546

Number at risk							
Intervention	692	632	607	577	555	535	509
Control	694	661	639	615	593	572	546

Figure 4: Cumulative incidence of unplanned readmission within 30 days and the competing event death without prior readmission within 30 days estimated by a) flexible parametric survival models or b) the non-parametric estimator ignoring stratification.

Table 9: Stratum-specific analysis of the primary outcome (unplanned readmission or death within 30 days after discharge) and unplanned readmission within 30 days after discharge together with its competing event (death without prior readmission). This analysis was included because there was evidence that the homogeneity assumption might not hold for these outcomes (i.e. that the effect of the intervention depended on the stratum). A negative risk difference would indicate a benefit of the intervention.

	Intervention group (N = 692)	Control group (N = 694)	Risk difference (95% CI)	P-value
	n (%, 95% CI)			
Unplanned readmission or death				
Intermediate risk				
Biel	29/136 (21%, 15 to 29%)	17/135 (13%, 7.9 to 19%)	8.7% (-0.1 to 17.6%)	0.06
Neuchatel	15/104 (14%, 8.8 to 23%)	22/104 (21%, 14 to 30%)	-6.7% (-17.1 to 3.6%)	0.20
Fribourg	15/103 (15%, 8.9 to 23%)	11/104 (11%, 5.9 to 18%)	4.0% (-5.0 to 13.0%)	0.39
Lausanne	20/129 (16%, 10 to 23%)	32/130 (25%, 18 to 33%)	-9.1% (-18.8 to 0.6%)	0.07
High risk				
Biel	17/61 (28%, 18 to 41%)	15/62 (24%, 15 to 37%)	3.7% (-11.8 to 19.2%)	0.64
Neuchatel	13/45 (29%, 17 to 44%)	5/43 (12%, 4.8 to 26%)	17.3% (0.9 to 33.6%)	0.045
Fribourg	14/45 (31%, 19 to 46%)	14/45 (31%, 19 to 46%)	0.0% (-19.1 to 19.1%)	1.00
Lausanne	22/69 (32%, 22 to 44%)	18/71 (25%, 16 to 37%)	6.5% (-8.4 to 21.5%)	0.39
Unplanned readmission				
Intermediate risk				
Biel	22/136 (17%, 12 to 25%)	17/135 (13%, 8.1 to 20%)	4.5% (-4.1 to 13%)	0.30
Neuchatel	14/104 (15%, 9.1 to 24%)	20/104 (19%, 13 to 29%)	-4.7% (-15 to 5.7%)	0.37
Fribourg	12/103 (12%, 7.3 to 21%)	10/104 (10%, 5.3 to 17%)	2.8% (-5.8 to 11%)	0.52
Lausanne	18/129 (14%, 9.4 to 22%)	31/130 (25%, 18 to 34%)	-10% (-20 to -0.7%)	0.036
High risk				
Biel	14/61 (24%, 15 to 38%)	14/62 (24%, 15 to 38%)	0.2% (-15 to 16%)	0.98
Neuchatel	12/45 (28%, 18 to 45%)	4/43 (9.2%, 3.6 to 23%)	19% (3.1 to 35%)	0.020
Fribourg	13/45 (30%, 19 to 47%)	10/45 (22%, 13 to 38%)	7.8% (-10 to 26%)	0.40
Lausanne	22/69 (33%, 23 to 46%)	18/71 (25%, 17 to 37%)	7.9% (-7.0 to 23%)	0.30
Death without unplanned readmission				
Intermediate risk				
Biel	7/136 (3.5%, 1.6 to 7.6%)	0/135 (1.9%, 0.8 to 4.6%)	1.6% (-0.6 to 3.8%)	0.15
Neuchatel	1/104 (2.0%, 0.6 to 6.4%)	2/104 (1.0%, 0.3 to 3.5%)	1.0% (-0.5 to 2.6%)	0.20
Fribourg	3/103 (2.6%, 1.0 to 7.2%)	1/104 (1.4%, 0.5 to 4.1%)	1.2% (-0.6 to 3.1%)	0.19
Lausanne	2/129 (1.6%, 0.5 to 5.1%)	1/130 (0.8%, 0.2 to 2.7%)	0.8% (-0.4 to 2.1%)	0.18
High risk				
Biel	3/61 (4.5%, 1.7 to 12%)	1/62 (2.4%, 0.8 to 7.1%)	2.2% (-1.0 to 5.3%)	0.18
Neuchatel	1/45 (2.9%, 0.7 to 12%)	1/43 (1.8%, 0.4 to 7.5%)	1.2% (-1.2 to 3.5%)	0.33
Fribourg	1/45 (7.2%, 3.0 to 18%)	4/45 (4.1%, 1.5 to 11%)	3.2% (-1.6 to 7.9%)	0.20
Lausanne	0/69 (0.0%, 0.0 to .%)	0/71 (0.0%, 0.0 to .%)	0.0% (-0.0 to 0.0%)	1.00

Table 10: Primary analysis of time to primary outcome using the restricted mean survival time (RMST) truncated at 30 days, i.e. the mean time to the primary outcome within 30 days. A positive RMST difference would indicate a benefit of the intervention.

	Intervention group (N = 692)	Control group (N = 694)	RMST difference (95% CI)	P-value
	RMST (95% CI), days			
Time to unplanned readmission or death	26.8 (26.3 to 27.3)	27.0 (26.5 to 27.6)	-0.23 (-0.94 to 0.47)	0.51

Table 11: Primary analysis of post-discharge health care utilization within 30 days after discharge. The incidence in each group is expressed per 30 person-days (i.e. over the observed period). An incidence rate ratio smaller than one would indicate a benefit of the intervention.

	Intervention group (N = 692)	Control group (N = 694)	Incidence rate ratio (95% CI)	P-value
	n (incidence, 95% CI)			
Number of unplanned hospital readmissions	135 (0.18, 0.15 to 0.21)	140 (0.18, 0.15 to 0.21)	1.02 (0.81 to 1.30)	0.85
Number of planned hospital readmissions	28 (0.04, 0.03 to 0.05)	36 (0.05, 0.03 to 0.06)	0.83 (0.50 to 1.37)	0.46
Number of unplanned hospitalization days	1118 (1.50, 1.42 to 1.59)	1191 (1.51, 1.42 to 1.60)	0.81 (0.57 to 1.15)	0.24
Number of planned hospitalization days	136 (0.18, 0.15 to 0.22)	202 (0.26, 0.22 to 0.29)	0.84 (0.39 to 1.81)	0.65
Number of emergency room visits	51 (0.07, 0.05 to 0.09)	55 (0.07, 0.05 to 0.09)	0.94 (0.62 to 1.43)	0.76
Number primary care provider visits	1103 (1.52, 1.43 to 1.61)	1158 (1.51, 1.43 to 1.60)	1.00 (0.92 to 1.09)	0.99

Table 12: Primary analysis of patient satisfaction with the quality of transition of care between hospital and home assessed by the three-item care transition measure (CTM-3) at 30 days based on multiply imputed data. Complete cases are shown for comparison. A positive risk difference would indicate a benefit of the intervention.

	Intervention group	Control group	Risk difference (95% CI)	P-value
	n (%, 95% CI)			
Mulitply imputed data	N = 692	N = 694		
Satisfied with quality of transition of care between hospital and home	575 (83%, 80 to 86%)	585 (84%, 81 to 87%)	-1.3% (-5.6 to 3.1%)	0.52
1) The hospital staff took my preferences and those of my family or caregiver into account.	617 (89%, 87 to 92%)	627 (90%, 88 to 93%)	-1.3% (-4.9 to 2.3%)	0.48
2) When I left the hospital, I had a good understanding of the things I was responsible for in managing my health.	644 (93%, 91 to 95%)	644 (93%, 91 to 95%)	0.2% (-3.1 to 3.5%)	0.60
3) When I left the hospital, I clearly understood the purpose for taking each of my medications.	638 (92%, 90 to 95%)	642 (93%, 90 to 95%)	-0.4% (-3.8 to 3.0%)	0.60
Complete cases	N = 536	N = 563		
Satisfied with quality of transition of care between hospital and home	453 (85%, 81 to 87%)	481 (85%, 82 to 88%)	-0.7% (-4.9 to 3.4%)	0.73
1) The hospital staff took my preferences and those of my family or caregiver into account.	481 (90%, 87 to 92%)	511 (91%, 88 to 93%)	-0.9% (-4.3 to 2.6%)	0.62
2) When I left the hospital, I had a good understanding of the things I was responsible for in managing my health.	504 (94%, 92 to 96%)	526 (93%, 91 to 95%)	0.5% (-2.4 to 3.4%)	0.72
3) When I left the hospital, I clearly understood the purpose for taking each of my medications.	501 (93%, 91 to 95%)	526 (93%, 91 to 95%)	0.2% (-2.7 to 3.1%)	0.89

Table 13: Primary analysis of the cost of readmission for patients with a readmission based on multiply imputed data. Complete cases are shown for comparison. The mean difference is calculated from a linear regression with robust standard errors, the mean ratio from a gamma regression. A negative mean difference and a mean ratio smaller than one would indicate a benefit of the intervention.

	Intervention group	Control group	Linear regression		Gamma regression	P-value
	mean (sd)		Mean difference (95% CI)	P-value	Mean ratio (95% CI)	
Mulitply imputed data	N = 127	N = 124				
Costs of readmission	15355 (20723)	15921 (24391)	-1075 (-5314 to 3165)	0.62	0.92 (0.72 to 1.18)	0.53
Complete cases	N = 124	N = 121				
Costs of readmission	15386 (14630)	15794 (16768)	-899 (-5105 to 3308)	0.67	0.94 (0.74 to 1.20)	0.61

6.1 Summary

- We did not find any evidence that the risk for unplanned readmission or death within 30 days after discharge was decreased in the intervention compared to the controls group (table 8). It was actually increased by 1.7% (95% CI -2.5 to 5.9%, p = 0.44).
- However, there was some evidence for heterogeneity between strata (i.e. site and readmission risk group) with a p-value of 0.05. The strata-specific analysis (table 9) indeed showed some heterogeneity with negative effects of the intervention on high risk patients in Neuchatel (and tendency-wise on intermediate risk patients in Biel), a tendency for some benefit on intermediate risk patients in Lausanne and not much evidence for any effect in the other strata.
- The risk for death within 30 days after discharge was increased in the intervention group by 2.0% (95% CI 0.1 to 4.0%), p = 0.043) (table 8).
- The risk for unplanned readmissions did not much differ between treatment groups (table 8). As for the primary outcome, some heterogeneity was observed with increased risk for unplanned readmission for high risk patients in Neuchatel but a reduced risk for intermediate risk patients in Lausanne.
- The competing event for unplanned readmissions—death without previous readmission—tended to be increased in the intervention group.
- We did not find evidence for an effect if the intervention on the time to unplanned readmission or death (table 10), the post-discharge health care utilization (table 11), patient satisfaction with the quality of transition of care between hospital and home (table 12), or the cost of readmission (table 13).

7 Secondary analyses

Table 14: Secondary analysis of the primary outcome (unplanned readmission or death within 30 days after discharge) and its components (deaths and readmissions within 30 days after discharge) based on the per-protocol set. Deaths without prior readmission is the competing event to unplanned readmission. A negative risk difference would indicate a benefit of the intervention. Only complete cases were considered and the number of patients was not the same for all outcomes.

	Intervention group (N = 631) n/N (%, 95% CI)	Control group (N = 645)	Risk difference (95% CI)	P-value	P-value for homogeneity
Unplanned readmission or death	134/608 (22%, 19 to 26%)	123/635 (19%, 16 to 23%)	2.6% (-1.9 to 7.1%)	0.25	0.045
Death	28/613 (4.6%, 3.2 to 6.5%)	15/637 (2.4%, 1.4 to 3.8%)	2.1% (0.1 to 4.2%)	0.037	0.35
Unplanned readmission	119/605 (20%, 17 to 23%)	114/635 (18%, 15 to 21%)	1.9% (-2.4 to 6.2%)	0.38	0.042
Death without unplanned readmission	12/605 (2.0%, 1.2 to 3.6%)	9/635 (1.4%, 0.7 to 2.7%)	0.6% (-0.8 to 2.1%)	0.39	0.13

Table 15: Strata-specific analysis of the primary outcome (unplanned readmission or death within 30 days after discharge) and unplanned readmission within 30 days after discharge together with its competing event (death without prior readmission) based on the per-protocol set. This analysis was included because there was evidence that the homogeneity assumption might not hold for these outcomes (i.e. that the effect of the intervention depended on the stratum). A negative risk difference would indicate a benefit of the intervention.

	Intervention group n (%, 95% CI)	Control group	Risk difference (95% CI)	P-value
Unplanned readmission or death	N = 608	N = 635		
Intermediate risk				
Biel	26/125 (21%, 15 to 29%)	14/122 (11%, 6.9 to 19%)	9.3% (0.2 to 18.4%)	0.047
Neuchatel	15/90 (17%, 10 to 26%)	22/103 (21%, 14 to 30%)	-4.7% (-15.7 to 6.3%)	0.41
Fribourg	11/76 (14%, 8.1 to 24%)	10/88 (11%, 6.2 to 20%)	3.1% (-7.2 to 13.4%)	0.55
Lausanne	17/117 (15%, 9.2 to 22%)	29/117 (25%, 18 to 33%)	-10.3% (-20.4 to -0.2%)	0.048
High risk				
Biel	17/57 (30%, 19 to 43%)	15/56 (27%, 17 to 40%)	3.0% (-13.6 to 19.6%)	0.72
Neuchatel	13/43 (30%, 18 to 46%)	5/42 (12%, 4.9 to 26%)	18.3% (1.5 to 35.2%)	0.039
Fribourg	13/35 (37%, 22 to 55%)	10/38 (26%, 14 to 43%)	10.8% (-10.4 to 32.1%)	0.32
Lausanne	22/65 (34%, 23 to 46%)	18/69 (26%, 17 to 38%)	7.8% (-7.7 to 23.2%)	0.33
Unplanned readmission	N = 605	N = 635		
Intermediate risk				
Biel	19/123 (16%, 10 to 24%)	14/122 (11%, 7.0 to 19%)	4.2% (-4.3 to 13%)	0.33
Neuchatel	14/90 (16%, 10 to 25%)	20/103 (20%, 13 to 29%)	-3.9% (-15 to 6.9%)	0.48
Fribourg	10/76 (14%, 7.6 to 24%)	9/88 (10%, 5.5 to 19%)	3.3% (-6.7 to 13%)	0.51
Lausanne	16/117 (14%, 8.7 to 22%)	28/117 (24%, 18 to 33%)	-11% (-20 to -0.6%)	0.038

continued on next page

Table 15: Strata-specific analysis of the primary outcome (unplanned readmission or death within 30 days after discharge) and unplanned readmission within 30 days after discharge together with its competing event (death without prior readmission) based on the per-protocol set. This analysis was included because there was evidence that the homogeneity assumption might not hold for these outcomes (i.e. that the effect of the intervention depended on the stratum). A negative risk difference would indicate a benefit of the intervention.

	Intervention group	Control group	Risk difference (95% CI)	P-value
	n (%, 95% CI)			
High risk				
Biel	14/57 (25%, 16 to 39%)	14/56 (24%, 15 to 38%)	0.3% (-15 to 16%)	0.97
Neuchatel	12/43 (28%, 18 to 46%)	4/42 (9.4%, 3.7 to 24%)	19% (2.8 to 35%)	0.021
Fribourg	12/34 (36%, 23 to 57%)	7/38 (18%, 9.3 to 35%)	18% (-2.1 to 38%)	0.08
Lausanne	22/65 (33%, 23 to 46%)	18/69 (26%, 17 to 38%)	7.2% (-7.9 to 22%)	0.35
Death without unplanned readmission				
Intermediate risk				
Biel	5/123 (2.4%, 1.0 to 6.2%)	0/122 (1.7%, 0.6 to 4.6%)	0.7% (-1.1 to 2.6%)	0.44
Neuchatel	1/90 (1.9%, 0.6 to 6.2%)	2/103 (1.3%, 0.4 to 4.3%)	0.7% (-0.9 to 2.2%)	0.39
Fribourg	1/76 (1.5%, 0.4 to 6.2%)	1/88 (1.0%, 0.2 to 4.4%)	0.5% (-0.8 to 1.7%)	0.47
Lausanne	1/117 (1.1%, 0.3 to 4.4%)	1/117 (0.7%, 0.1 to 2.9%)	0.4% (-0.5 to 1.3%)	0.37
High risk				
Biel	3/57 (4.2%, 1.5 to 12%)	1/56 (2.9%, 1.0 to 8.5%)	1.3% (-1.9 to 4.5%)	0.41
Neuchatel	1/43 (2.6%, 0.6 to 11%)	1/42 (2.1%, 0.5 to 9.0%)	0.5% (-1.6 to 2.7%)	0.63
Fribourg	0/34 (4.6%, 1.4 to 15%)	3/38 (3.7%, 1.1 to 12%)	0.9% (-2.8 to 4.7%)	0.63
Lausanne	0/65 (0.0%, 0.0 to .%)	0/69 (0.0%, 0.0 to .%)	0.0% (-0.0 to 0.0%)	1.00

Table 16: Secondary analysis of time to primary outcome based on the per-protocol set. using the restricted mean survival time (RMST) truncated at 30 days, i.e. the mean time to the primary outcome within 30 days. A positive RMST difference would indicate a benefit of the intervention.

	Intervention group (N = 608)	Control group (N = 635)	RMST difference (95% CI)	P-value
	RMST (95% CI), days			
Time to unplanned readmission or death	26.5 (25.9 to 27.1)	27.0 (26.4 to 27.5)	-0.48 (-1.22 to 0.25)	0.20

Table 17: Secondary analysis of post-discharge health care utilization within 30 days after discharge based on the per-protocol set. The incidence in each group is expressed per 30 person-days (i.e. over the observed period). An incidence rate ratio smaller than one would indicate a benefit of the intervention.

	Intervention group (N = 631)		Control group (N = 645)		Incidence rate ratio	P-value
	non-missing	n (incidence, 95% CI)	non-missing	n (incidence, 95% CI)	(95% CI)	
Number of unplanned hospital readmissions	605	127 (0.19, 0.16 to 0.22)	635	130 (0.18, 0.15 to 0.21)	1.05 (0.82 to 1.35)	0.68
Number of planned hospital readmissions	603	26 (0.04, 0.02 to 0.06)	632	32 (0.04, 0.03 to 0.06)	0.89 (0.52 to 1.53)	0.68
Number of unplanned hospitalization days	605	1041 (1.53, 1.43 to 1.62)	635	1071 (1.45, 1.37 to 1.54)	0.84 (0.58 to 1.21)	0.35
Number of planned hospitalization days	603	124 (0.18, 0.15 to 0.22)	632	158 (0.22, 0.18 to 0.25)	0.66 (0.31 to 1.42)	0.29
Number of emergency room visits	603	47 (0.07, 0.05 to 0.09)	630	50 (0.07, 0.05 to 0.09)	0.97 (0.62 to 1.53)	0.90
Number primary care provider visits	587	1020 (1.53, 1.44 to 1.63)	624	1091 (1.53, 1.44 to 1.62)	1.00 (0.92 to 1.10)	0.92

Table 18: Secondary analysis based on the per-protocol set of patient satisfaction with the quality of transition of care between hospital and home assessed by the three-item care transition measure (CTM-3) at 30 days using complete cases. A positive risk difference would indicate a benefit of the intervention.

	Intervention group (N = 491)	Control group (N = 531)	Risk difference (95% CI)	P-value
	n (%, 95% CI)			
Satisfied with quality of transition of care between hospital and home	412 (84%, 80 to 87%)	456 (86%, 83 to 89%)	-1.6% (-5.9 to 2.7%)	0.46
1) The hospital staff took my preferences and those of my family or caregiver into account.	437 (89%, 86 to 91%)	482 (91%, 88 to 93%)	-1.4% (-5.1 to 2.2%)	0.44
2) When I left the hospital, I had a good understanding of the things I was responsible for in managing my health.	461 (94%, 91 to 96%)	499 (94%, 92 to 96%)	-0.2% (-3.2 to 2.7%)	0.88
3) When I left the hospital, I clearly understood the purpose for taking each of my medications.	459 (93%, 91 to 95%)	498 (94%, 91 to 96%)	-0.2% (-3.2 to 2.7%)	0.88

Table 19: Secondary analysis based on the per-protocol set of the cost of readmission for patients with a readmission using complete cases. The mean difference is calculated from a linear regression with robust standard errors, the mean ratio from a gamma regression. A negative mean difference and a mean ratio smaller than one would indicate a benefit of the intervention.

	Intervention group (N = 117)	Control group (N = 112)	Linear regression Mean difference (95% CI)	P-value	Gamma regression Mean ratio (95% CI)	P-value
	mean (sd)					
Costs of readmission	15008 (14688)	15712 (16729)	-1226 (-5587 to 3135)	0.58	0.92 (0.71 to 1.19)	0.51

7.1 Summary

- The secondary analysis on the per-protocol set confirmed the findings form the main analysis.
- There was no significant effect of the intervention on the primary outcome or on unplanned readmission but the intervention was associated with an increased risk for death.
- There was no effect on any of the other outcomes.

8 Sensitivity analyses

Table 20: Sensitivity analysis of the primary outcome (unplanned readmission or death within 30 days after discharge) excluding early readmissions or deaths (i.e. patients who died or had a readmission up to the day after index discharge). A negative risk difference would indicate a benefit of the intervention.

	Intervention group (N = 681) n (%, 95% CI)	Control group (N = 688)	Risk difference (95% CI)	P-value	P-value for for homogeneity
Unplanned readmission or death	134 (20%, 17 to 23%)	128 (19%, 16 to 22%)	1.1% (-3.0 to 5.3%)	0.60	0.043

Table 21: Strata-specific sensitivity analysis of the primary outcome (unplanned readmission or death within 30 days after discharge) excluding early readmissions or deaths. This analysis was included because there was evidence that the homogeneity assumption might not hold (i.e. that the effect of the intervention depended on the stratum). A negative risk difference would indicate a benefit of the intervention.

	Intervention group (N = 681) n (%, 95% CI)	Control group (N = 688)	Risk difference (95% CI)	P-value
Unplanned readmission or death				
Intermediate risk				
Biel	26/133 (20%, 14 to 27%)	16/134 (12%, 7.4 to 19%)	7.6% (-1.1 to 16.3%)	0.09
Neuchatel	12/101 (12%, 6.8 to 20%)	20/102 (20%, 13 to 29%)	-7.7% (-17.7 to 2.2%)	0.13
Fribourg	14/102 (14%, 8.2 to 22%)	10/103 (10%, 5.3 to 17%)	4.0% (-4.8 to 12.8%)	0.37
Lausanne	18/127 (14%, 9.1 to 21%)	30/128 (23%, 17 to 32%)	-9.3% (-18.8 to 0.3%)	0.06
High risk				
Biel	16/60 (27%, 17 to 39%)	15/62 (24%, 15 to 37%)	2.5% (-13.0 to 17.9%)	0.75
Neuchatel	13/45 (29%, 17 to 44%)	5/43 (12%, 4.8 to 26%)	17.3% (0.9 to 33.6%)	0.045
Fribourg	13/44 (30%, 18 to 45%)	14/45 (31%, 19 to 46%)	-1.6% (-20.7 to 17.5%)	0.87
Lausanne	22/69 (32%, 22 to 44%)	18/71 (25%, 16 to 37%)	6.5% (-8.4 to 21.5%)	0.39

Table 22: Sensitivity analysis without adjusting for the stratification factors (crude analysis) of all outcome. Binary outcomes are presented with risks and risk difference (in time to primary outcome with the restricted mean survival time (RMST) truncated at 30 days and count outcomes with incidence (per 30 patient-days) and incidence rate ratio.

	Intervention group (N = 692)	Control group (N = 694)	Effect of intervention	P-value
	n (%, 95% CI)		Risk difference (95% CI)	
Unplanned readmission or death	145 (21%, 18 to 24%)	134 (19%, 17 to 22%)	1.6% (-2.6 to 5.9%)	0.44
Death	32 (4.6%, 3.3 to 6.5%)	18 (2.6%, 1.6 to 4.1%)	2.0% (0.05 to 4.1%)	0.043
Unplanned readmission	127 (19%, 17 to 23%)	124 (18%, 15 to 21%)	1.3% (-2.9 to 5.4%)	0.55
Death without unplanned readmission	18 (2.7%, 1.7 to 4.3%)	10 (1.5%, 0.8 to 2.7%)	1.3% (-0.3 to 2.8%)	0.10
Satisfied with quality of transition of care between hospital and home	575 (83%, 80 to 86%)	585 (84%, 81 to 87%)	-1.2% (-5.6 to 3.2%)	0.54
	RMST (95% CI), days	RMST (95% CI), days	RMST difference (95% CI)	
Time to unplanned readmission or death	26.8 (26.3 to 27.3)	27.0 (26.5 to 27.6)	-0.24 (-0.95 to 0.48)	0.51
	n (incidence, 95% CI)	n (incidence, 95% CI)	Incidence rate ratio (95% CI)	
Number of unplanned hospital readmissions	135 (0.18, 0.15 to 0.21)	140 (0.18, 0.15 to 0.21)	1.02 (0.80 to 1.31)	0.84
Number of planned hospital readmissions	28 (0.04, 0.03 to 0.05)	36 (0.05, 0.03 to 0.06)	0.82 (0.48 to 1.39)	0.44
Number of unplanned hospitalization days	1118 (1.50, 1.42 to 1.59)	1191 (1.51, 1.42 to 1.60)	1.00 (0.92 to 1.08)	0.94
Number of planned hospitalization days	136 (0.18, 0.15 to 0.22)	202 (0.26, 0.22 to 0.29)	0.71 (0.57 to 0.89)	0.002
Number of emergency room visits	51 (0.07, 0.05 to 0.09)	55 (0.07, 0.05 to 0.09)	0.98 (0.65 to 1.46)	0.91
Number primary care provider visits	1103 (1.52, 1.43 to 1.61)	1158 (1.51, 1.43 to 1.60)	1.00 (0.92 to 1.09)	0.94

Table 23: Crude analysis of the count outcomes using the same methods as for the primary analysis, i.e. negative binomial regression with robust standard errors and adjustment for zero-inflation for unplanned hospitalization days. The incidence in each group is expressed per 30 person-days (i.e. over the observed period).

	Intervention group (N = 692)	Control group (N = 694)	Incidence rate ratio (95% CI)	P-value
	n (incidence, 95% CI)			
Number of unplanned hospital readmissions	135 (0.18, 0.15 to 0.21)	140 (0.18, 0.15 to 0.21)	1.02 (0.80 to 1.30)	0.85
Number of planned hospital readmissions	28 (0.04, 0.03 to 0.05)	36 (0.05, 0.03 to 0.06)	0.82 (0.49 to 1.38)	0.46
Number of unplanned hospitalization days	1118 (1.50, 1.42 to 1.59)	1191 (1.51, 1.42 to 1.60)	0.82 (0.56 to 1.20)	0.30
Number of planned hospitalization days	136 (0.18, 0.15 to 0.22)	202 (0.26, 0.22 to 0.29)	0.78 (0.36 to 1.67)	0.52
Number of emergency room visits	51 (0.07, 0.05 to 0.09)	55 (0.07, 0.05 to 0.09)	0.97 (0.64 to 1.47)	0.88
Number primary care provider visits	1103 (1.52, 1.43 to 1.61)	1158 (1.51, 1.43 to 1.60)	1.00 (0.92 to 1.10)	0.94

Table 24: Sensitivity analysis of the primary outcome (unplanned readmission or death within 30 days after discharge) using survival methods, i.e. flexible parametric survival models (parametric) or the Kaplan-Meier estimator (non-parametric) at 30 days. A negative risk difference would indicate a benefit of the intervention.

	Intervention group (N = 692)	Control group (N = 694)	Risk difference (95% CI)	P-value
	n (%, 95% CI)			
Non-parametric	145 (22%, 19 to 25%)	134 (19%, 17 to 22%)	2.6% (-1.7 to 7.0%)	0.23
Parametric	145 (22%, 19 to 25%)	134 (20%, 17 to 23%)	2.5% (-1.8 to 6.8%)	0.25

Table 25: Sensitivity analysis of unplanned readmission within 30 days after discharge in the presence of the competing risk of death with a non-parametric method (i.e. the cumulative incidence function at 30 days), without adjusting for the stratification factors. A negative risk difference would indicate a benefit of the intervention.

	Intervention group (N = 692)	Control group (N = 694)	Risk difference (95% CI)	P-value
	n (%, 95% CI)			
Unplanned readmission	127 (19%, 16 to 22%)	124 (18%, 15 to 21%)	1.1% (-3.1 to 5.3%)	0.61
Death without unplanned readmission	18 (2.7%, 1.7 to 4.2%)	10 (1.5%, 0.8 to 2.6%)	1.3% (-0.3 to 2.8%)	0.11

Table 26: Sensitivity analysis of patient satisfaction with the quality of transition of care at 30 days assuming the worst case for patient that died (i.e. a negative respond to all question of the CTM-3) using multiply imputeted data or only complete cases. A positive risk difference would indicate a benefit of the intervention.

	Intervention group	Control group	Risk difference (95% CI)	P-value
	n (%, 95% CI)			
Mulitply imputed data	N = 692	N = 694		
Satisfied with quality of transition of care between hospital and home	549 (79%, 76 to 83%)	569 (82%, 79 to 85%)	-2.8% (-7.2 to 1.7%)	0.22
1) The hospital staff took my preferences and those of my family or caregiver into account.	587 (85%, 82 to 88%)	609 (88%, 85 to 90%)	-3.0% (-6.8 to 0.9%)	0.13
2) When I left the hospital, I had a good understanding of the things I was responsible for in managing my health.	613 (89%, 86 to 91%)	625 (90%, 88 to 92%)	-1.5% (-5.1 to 2.1%)	0.41
3) When I left the hospital, I clearly understood the purpose for taking each of my medications.	608 (88%, 85 to 91%)	624 (90%, 87 to 92%)	-2.0% (-5.7 to 1.7%)	0.28
Complete cases	N = 568	N = 581		
Satisfied with quality of transition of care between hospital and home	453 (80%, 76 to 83%)	481 (83%, 80 to 86%)	-2.8% (-7.2 to 1.6%)	0.21
1) The hospital staff took my preferences and those of my family or caregiver into account.	481 (85%, 81 to 87%)	511 (88%, 85 to 90%)	-3.1% (-7.0 to 0.9%)	0.13
2) When I left the hospital, I had a good understanding of the things I was responsible for in managing my health.	504 (89%, 86 to 91%)	526 (91%, 88 to 93%)	-1.8% (-5.4 to 1.7%)	0.30
3) When I left the hospital, I clearly understood the purpose for taking each of my medications.	501 (88%, 85 to 91%)	526 (91%, 88 to 93%)	-2.2% (-5.8 to 1.3%)	0.22

8.1 Summary

- The sensitivity analyses confirmed the findings form the main analysis.
- In particular, also crude analysis (table 22) and non-parametric survival analysis (tables 24 and 25.) confirmed that the intervention did not have an effect on the primary ouctcome but did increase the risk of death within 30 days .
- In crude analysis using an exact Poisson test, the number of planned hospitalization days appeared to be lower in the intervention group (table 22). However, there was clear overdispersion and the effect disappeared if robust standard errors and/or a negative binomial regression was used (table 23).

9 Subgroup analyses

	Intervention *n/N (%)*	Control *n/N (%)*	Risk difference (95% CI)	P−value	P−value for interaction
Overall	145/692 (21%)	134/694 (19%)	1.7% (−2.5 to 5.9%)	0.44	
Risk for readmission					0.15
Intermediate	72/448 (16%)	76/450 (17%)	−0.7% (−5.6 to 4.2%)	0.79	
High	73/244 (30%)	58/244 (24%)	6.1% (−1.7 to 14%)	0.13	
Clinical site					0.19
Biel	46/197 (23%)	32/197 (16%)	7.5% (−0.2 to 15%)	0.06	
Neuchatel	28/149 (19%)	27/147 (18%)	−0.1% (−9.2 to 8.9%)	0.98	
Fribourg	29/148 (20%)	25/149 (17%)	3.3% (−4.9 to 11%)	0.43	
Lausanne	42/198 (21%)	50/201 (25%)	−4.5% (−13 to 3.7%)	0.28	
Diabetes					0.18
No	108/492 (22%)	93/510 (18%)	3.4% (−1.5 to 8.2%)	0.17	
Yes	37/200 (19%)	41/184 (22%)	−3.4% (−12 to 4.7%)	0.41	
Chronic heart failure					0.38
No	102/487 (21%)	89/500 (18%)	2.8% (−2.0 to 7.5%)	0.26	
Yes	43/205 (21%)	45/194 (23%)	−1.8% (−10 to 6.3%)	0.66	
COPD					0.045
No	116/587 (20%)	115/576 (20%)	−0.2% (−4.7 to 4.3%)	0.94	
Yes	29/105 (28%)	19/118 (16%)	12% (0.9 to 22%)	0.034	
Cancer					0.17
No	76/404 (19%)	57/388 (15%)	4.2% (−0.9 to 9.4%)	0.11	
Yes	69/288 (24%)	77/306 (25%)	−1.8% (−8.7 to 5.0%)	0.60	
Living place					0.023
Not in nursing home	123/610 (20%)	126/631 (20%)	−0.1% (−4.5 to 4.3%)	0.97	
In nursing home	22/82 (27%)	8/63 (13%)	16% (3.5 to 28%)	0.011	
Living status					0.51
Not alone	82/389 (21%)	78/383 (20%)	0.4% (−5.2 to 6.1%)	0.88	
Alone	63/303 (21%)	56/311 (18%)	3.2% (−2.9 to 9.3%)	0.30	
Semi−private or private insurance					0.30
No	125/575 (22%)	109/568 (19%)	2.6% (−2.0 to 7.2%)	0.26	
Yes	20/117 (17%)	25/126 (20%)	−3.0% (−12 to 6.4%)	0.53	

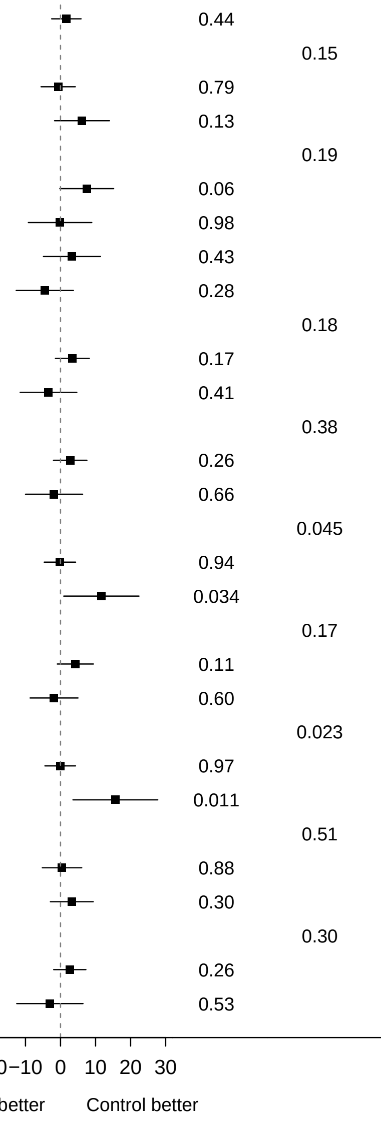

Figure 5: Subgroup analysis of the primary outcome (unplanned readmission or death within 30 days after discharge).

	Intervention *n/N (%)*	Control	Risk difference (95% CI)	P−value	P−value for interaction
Overall	145/692 (21%)	134/694 (19%)	1.7% (−2.5 to 5.9%)	0.44	
Risk for readmission					0.13
Intermediate	72/448 (16%)	76/450 (17%)	−1.0% (−5.9 to 3.9%)	0.68	
High	73/244 (30%)	58/244 (24%)	7.8% (−0.2 to 16%)	0.05	
Clinical site					0.31
Biel	46/197 (23%)	32/197 (16%)	7.2% (−0.7 to 15%)	0.07	
Neuchatel	28/149 (19%)	27/147 (18%)	0.4% (−8.5 to 9.3%)	0.93	
Fribourg	29/148 (20%)	25/149 (17%)	2.8% (−5.8 to 11%)	0.53	
Lausanne	42/198 (21%)	50/201 (25%)	−3.6% (−12 to 4.7%)	0.39	
Diabetes					0.12
No	108/492 (22%)	93/510 (18%)	3.6% (−1.4 to 8.5%)	0.15	
Yes	37/200 (19%)	41/184 (22%)	−3.8% (−12 to 4.3%)	0.36	
Chronic heart failure					0.27
No	102/487 (21%)	89/500 (18%)	3.1% (−1.8 to 8.0%)	0.22	
Yes	43/205 (21%)	45/194 (23%)	−1.5% (−10 to 6.7%)	0.72	
COPD					0.05
No	116/587 (20%)	115/576 (20%)	−0.3% (−4.9 to 4.2%)	0.88	
Yes	29/105 (28%)	19/118 (16%)	13% (2.0 to 24%)	0.021	
Cancer					0.22
No	76/404 (19%)	57/388 (15%)	4.1% (−1.1 to 9.3%)	0.12	
Yes	69/288 (24%)	77/306 (25%)	−1.6% (−8.5 to 5.3%)	0.65	
Living place					0.041
Not in nursing home	123/610 (20%)	126/631 (20%)	0.1% (−4.3 to 4.5%)	0.97	
In nursing home	22/82 (27%)	8/63 (13%)	16% (2.8 to 28%)	0.021	
Living status					0.63
Not alone	82/389 (21%)	78/383 (20%)	0.7% (−5.1 to 6.4%)	0.82	
Alone	63/303 (21%)	56/311 (18%)	3.1% (−3.1 to 9.3%)	0.32	
Semi−private or private insurance					0.34
No	125/575 (22%)	109/568 (19%)	2.5% (−2.1 to 7.2%)	0.29	
Yes	20/117 (17%)	25/126 (20%)	−1.4% (−11 to 8.4%)	0.78	

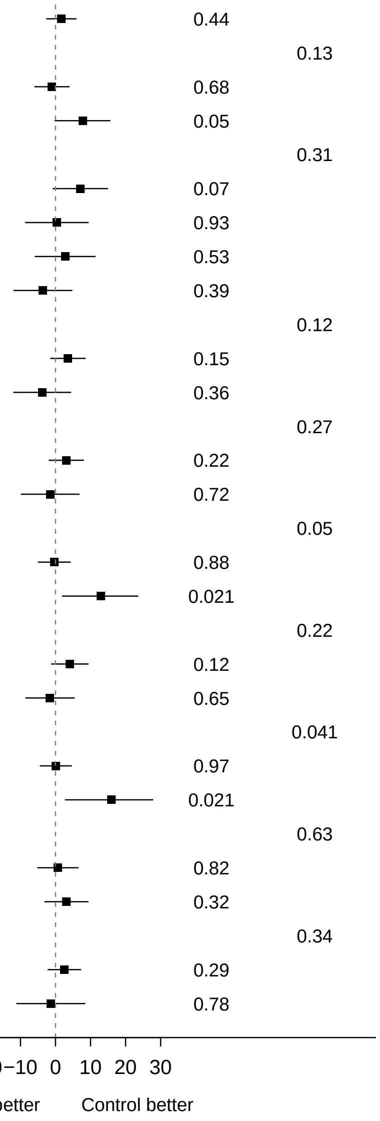

Figure 6: Subgroup analysis of the primary outcome (unplanned readmission or death within 30 days after discharge) based on Mantel-Haenszel methodology.

9.1 Summary

- The subgroup analyses showed the heterogeneity between risk group and sites (which has been seen before, section 6) although the interaction of risk group and site alone was not significant. The intervention tended to have a negative effect on high risk patients and in Biel.
- The intervention increased the risk for unplanned readmission or death for patients with COPD and living in nursing homes.

Appendix

R session info:

```
R version 4.0.3 (2020-10-10)
Platform: x86_64-w64-mingw32/x64 (64-bit)
Running under: Windows 10 x64 (build 18363)

Matrix products: default

locale:
[1] LC_COLLATE=German_Switzerland.1252  LC_CTYPE=German_Switzerland.1252
[3] LC_MONETARY=German_Switzerland.1252 LC_NUMERIC=C
[5] LC_TIME=C

attached base packages:
[1] splines   stats     graphics  grDevices utils     datasets  methods
[8] base

other attached packages:
 [1] RColorBrewer_1.1-3 lubridate_1.8.0    stringr_1.4.1      Hmisc_4.7-0
 [5] ggplot2_3.3.6      Formula_1.2-4      survival_3.3-1     lattice_0.20-45
 [9] lspline_1.0-0      locfit_1.5-9.4     lmerTest_3.1-3     xtable_1.8-4
[13] xlsx_0.6.5         merTools_0.5.2     arm_1.13-1         MASS_7.3-56
[17] lme4_1.1-29        Matrix_1.4-1

loaded via a namespace (and not attached):
 [1] tidyr_1.2.0         foreach_1.5.2       shiny_1.7.2
 [4] assertthat_0.2.1    latticeExtra_0.6-30 broom.mixed_0.2.9.4
 [7] xlsxjars_0.6.1      globals_0.16.1      numDeriv_2016.8-1.1
[10] pillar_1.8.1        backports_1.4.1     glue_1.6.2
[13] digest_0.6.29       checkmate_2.0.0     promises_1.2.0.1
[16] minqa_1.2.4         colorspace_2.0-3    htmltools_0.5.2
[19] httpuv_1.6.5        pkgconfig_2.0.3     broom_1.0.1
[22] listenv_0.8.0       purrr_0.3.4         mvtnorm_1.1-3
[25] scales_1.2.1        jpeg_0.1-9          later_1.3.0
[28] htmlTable_2.4.1     tibble_3.1.6        generics_0.1.3
[31] ellipsis_0.3.2      withr_2.5.0         furrr_0.3.1
[34] nnet_7.3-17         cli_3.2.0           magrittr_2.0.3
[37] mime_0.12           deldir_1.0-6        future_1.28.0
[40] fansi_1.0.3         parallelly_1.32.1   nlme_3.1-157
[43] forcats_0.5.2       foreign_0.8-82      data.table_1.14.2
[46] tools_4.0.3         lifecycle_1.0.1     interp_1.0-33
[49] munsell_0.5.0       cluster_2.1.3       compiler_4.0.3
[52] rlang_1.0.2         blme_1.0-5          grid_4.0.3
[55] nloptr_2.0.0        rstudioapi_0.14     iterators_1.0.14
[58] htmlwidgets_1.5.4   base64enc_0.1-3     boot_1.3-28
[61] gtable_0.3.1        codetools_0.2-18    abind_1.4-5
[64] DBI_1.1.3           R6_2.5.1            gridExtra_2.3
[67] knitr_1.40          dplyr_1.0.8         fastmap_1.1.0
```

```
[70] utf8_1.2.2        stringi_1.7.6      rJava_1.0-6
[73] parallel_4.0.3    Rcpp_1.0.8.3       vctrs_0.4.1
[76] rpart_4.1.16      png_0.1-7          xfun_0.30
[79] tidyselect_1.1.2  coda_0.19-4
```

Bibliography

[1] StataCorp. *Stata Statistical Software: Release 15*. StataCorp, College Station, TX: StataCorp LP., 2017.

[2] R Core Team. *R: A Language and Environment for Statistical Computing*. R Foundation for Statistical Computing, Vienna, Austria, 2016. URL http://www.R-project.org. ISBN 3-900051-07-0.

[3] Bernhard Klingenberg. A new and improved confidence interval for the mantel-haenszel risk difference. *Stat Med*, 33(17):2968–2983, Jul 2014. doi: 10.1002/sim.6122. URL http://dx.doi.org/10.1002/sim.6122.

[4] Patrick Royston, Paul C Lambert, et al. *Flexible parametric survival analysis using Stata: beyond the Cox model*. Stata College Station, Texas, 2011.

[5] Sally R. Hinchliffe and Paul C. Lambert. Extending the flexible parametric survival model for competing risks. *The Stata Journal*, (2):344–355, 2013. 13.

[6] Patrick Royston and Mahesh KB Parmar. Restricted mean survival time: an alternative to the hazard ratio for the design and analysis of randomized trials with a time-to-event outcome. *BMC Med Res Methodol*, 13(1), dec 2013. doi: 10.1186/1471-2288-13-152. URL http://dx.doi.org/10.1186/1471-2288-13-152.

[7] Jahar B Choudhury. Non-parametric confidence interval estimation for competing risks analysis: application to contraceptive data. *Statistics in medicine*, 21(8):1129–1144, 2002.

[8] Donald B Rubin. *Multiple imputation for nonresponse in surveys*, volume 81. John Wiley & Sons, 2004.
