# Supplementary material for: Effects of a Multimodal Transitional Care Intervention in Patients at High Risk of Readmission: The TARGET-READ Randomized Clinical Trial
Source: JAMA Intern Med. 2023 May 1;183(7):658–68. doi: 10.1001/jamainternmed.2023.0791 (PMC10152373; doi:10.1001/jamainternmed.2023.0791)
Supplement: Supplement 2. — eTable 1. Simplified HOSPITAL score Switzerland. eTable 2. Summary of Discharge Procedures for the Usual Care Groups eTable 3. Pre-Discharge Component of the Intervention eTable 4. Follow-Up Phone Call eTable 5. Secondary Analysis (Per-Protocol) of Main Outcomes at 30-Days eTable 6. Strata-Specific Primary Analysis (Intent-to-Treat) for the Primary Outcome eTable 7. Strata-Specific Secondary Analysis (Per Protocol) for the Primary Outcome eTable 8. Main Diagnoses of Unplanned Readmissions eTable 9. Sensitivity Analyses; Primary Outcome Excluding Early Readmissions or Deaths eTable 10. Sensitivity Analyses; All Outcomes Without Adjusting for Stratification Factors eTable 11. Sensitivity Analyses; Primary Outcome Using Survival Methods by Calculating the Risk Difference at 30 Days From Flexible Parametric Survival Models eTable 12. Sensitivity Analyses; Assuming a Negative CTM-3 Score Result for Patients Who Died eTable 13. Sensitivity Analyses for Readmission Costs Using the Mean Ratio Calculated From a Gamma Regression eTable 14. Sensitivity Analysis for Unplanned Readmission and Death, Excluding Patients Living in Nursing Homes [file jamainternmed-e230791-s002.pdf]

Supplemental Online Content

Donzé J, John G, Genné D, et al. Effects of a multimodal transitional care intervention in patients at high risk of readmission. *JAMA Intern Med.* Published online May 1, 2023. doi:10.1001/jamainternmed.2023.0791

eTable 1. Simplified HOSPITAL score Switzerland.

eTable 2. Summary of Discharge Procedures for the Usual Care Groups

eTable 3. Pre-Discharge Component of the Intervention

eTable 4. Follow-Up Phone Call

eTable 5. Secondary Analysis (Per-Protocol) of Main Outcomes at 30-Days

eTable 6. Strata-Specific Primary Analysis (Intent-to-Treat) for the Primary Outcome

eTable 7. Strata-Specific Secondary Analysis (Per Protocol) for the Primary Outcome

eTable 8. Main Diagnoses of Unplanned Readmissions

eTable 9. Sensitivity Analyses; Primary Outcome Excluding Early Readmissions or Deaths

eTable 10. Sensitivity Analyses; All Outcomes Without Adjusting for Stratification Factors

eTable 11. Sensitivity Analyses; Primary Outcome Using Survival Methods by Calculating the Risk Difference at 30 Days From Flexible Parametric Survival Models

eTable 12. Sensitivity Analyses; Assuming a Negative CTM-3 Score Result for Patients Who Died

eTable 13. Sensitivity Analyses for Readmission Costs Using the Mean Ratio Calculated From a Gamma Regression

eTable 14. Sensitivity Analysis for Unplanned Readmission and Death, Excluding Patients Living in Nursing Homes

© 2023 Donzé J et al. *JAMA Internal Medicine*.

This supplemental material has been provided by the authors to give readers additional information about their work.

© 2023 Donzé J et al. *JAMA Internal Medicine*.

eTable 1: Simplified HOSPITAL score for Switzerland

Low hemoglobin level (< 12 g/dl))	1
Discharge from an oncology service or cancer diagnosis	2
Low sodium level at discharge (< 135 mEq/L))	1
Index admission type: urgent or emergent (non-elective)	1
No. of hospital admissions during the previous year<br>0–1<br>2–5<br>> 5	 0<br>2<br>5
Length of stay ≥ 8 days	2

© 2023 Donzé J et al. *JAMA Internal Medicine*.

eTable 2: Summary of discharge procedures performed from 2018–2020 for the **usual care groups** in the four participating hospital centers

	**Center 1**	**Center 2**	**Center 3**	**Center 4**
**Hospital information**				
**Type of hospital**	Mid-sized community teaching hospital	Mid-sized community teaching hospital	Mid-sized community teaching hospital	Large university teaching hospital
**Center size**	253 acute beds	200 acute beds	314 acute beds	1500 beds
**Number of beds in internal medicine wards**	108	75	126	180
**Language spoken**	German > French	French	French > German	French
**Documents sent to primary care physician**				
**Medical summary**	Systematic	Systematic	Systematic	Systematic
**Timely medical summary**	1–3 days	Mean time between discharge and document sent: 20 days	Short summary within 1 week. Complete summary within 3 weeks	Mean time between discharge and discharge letter sent: less than 7 days
**Medical prescription**	Systematic	Systematic	Systematic	Systematic
**Discharge prescription**				
**Medication reconciliation**	Recommended<br>Not systemically checked	Recommended<br>Not systemically checked	Recommended<br>Not systemically checked	Recommended<br>Not systemically checked
**Team-based pharmacists**	Yes*	No	No	Yes*
**Drug-drug interaction check**	Yes	Not systemically performed, but checking for interaction possible in the electronic patient prescription system.	Not systemically performed, but checking for interaction possible in the electronic prescription system.	Yes, included in the electronic patient prescription system.
**Post-discharge follow-up**				
**Follow-up phone call**	Not performed	Not performed	Not performed	Not performed
**Follow-up consultation with primary care physician**	Not organized at discharge	Not organized at discharge	Not organized at discharge	Not organized at discharge
**Discharge care center (e.g. ,a phone number that a patient can call with post-discharge questions)**	Non-existant	Non-existant	Non-existant	Non-existant

© 2023 Donzé J et al. *JAMA Internal Medicine*.

Patient education				
**Patient education on their primary diagnosis resulting in hospital admission**	Recommended but depends on patient's educational level or cognition	Recommended Patient's understanding and knowledge not systematically checked	Recommended Patient's understanding and knowledge not systematically checked	Not standardized
**Patient education on their medication at discharge**	Recommended but depends on patient's educational level or cognition	Recommended Patient's understanding and knowledge not systematically checked	Recommended Patient's understanding and knowledge not systematically checked	Recommended Patient's understanding and knowledge not systematically checked
**Patient education on their secondary new diagnosis (during hospital admission)**	Recommended but not systematically performed	Not systematically performed	Not systematically performed	Not systematically performed
**Patient education on their main comorbid condition**	Not systematically performed	Not performed	Not performed	Not systematically performed
**Specialized discharged nurses**	No	No	No	No

* Team-based pharmacists were available during the hospital stay, not specifically at discharge.

© 2023 Donzé J et al. *JAMA Internal Medicine*.

eTable 3: Pre-discharge component of the intervention. Only for the intervention group.

	Intervention group (**N = 692**)
Basic information about the patient's main diseases was given* (Yes)	688 (99%)
Specific information/leaflet on the patient's comorbidities was given	
• Heart failure	196 (28%)
• Coronary disease	184 (27%)
• Peripheral artery disease	75 (11%)
• Atrial fibrillation	133 (19%)
• Thromboembolism	71 (10%)
• Chronic obstructive pulmonary disease	99 (14%)
• Stroke	43 (6.2%)
• Gastrointestinal bleeding	65 (9.4%)
• Chronic renal failure	202 (29%)
• Liver cirrhosis	48 (6.9%)
• Diabetes	200 (29%)
• Other	119 (17%)
• Non-listed comorbidity	102 (15%)
Has a medication reconciliation been performed?* (Yes)	681 (98%)
Was a medication discrepancy noticed? (Yes)	235 (34%)
Has patient education about general health recommendations been performed?* (Yes)	690 (100%)
Has a post-discharge follow-up consultation with a treating physician been planned?* (Yes)	596 (86%)
Has a discharge summary been sent to the treating physician?* (Yes)	644 (93%)
Barriers to a safe discharge has been explored* (Yes)	682 (99%)

*Information missing for one patient

© 2023 Donzé J et al. *JAMA Internal Medicine.*

eTable 4: Follow-up phone call. Answers only available for patients reached by phone.

	Intervention group (**N = 692**)	
	Follow-up phone call on **day 3**	Follow-up phone call on **day 14**
Patient reached by phone	661 (96%)	630 (91%)
Reason why the patient was not reached		
No answer, not done	6 (19%)	13 (21%)
Death	1 (3.2%)	19 (31%)
Readmission	12 (39%)	10 (16%)
Withdrawal of consent	5 (16%)	12 (19%)
Lost to follow-up	0 (0.0%)	0 (0.0%)
Lost interest	0 (0.0%)	1 (1.6%)
Not discharged home or to a nursing home	7 (23%)	7 (11%)
Condition's status since discharge		
Deteriorated or somewhat deteriorated	60 (9.1%)	72 (11%)
Improved or somewhat improved	250 (38%)	295 (47%)
Stable	298 (45%)	172 (27%)
New symptom	53 (8.0%)	91 (14%)
Have you felt more pain since your discharge from hospital? (Yes)	89 (13%)	113 (18%)
How has your mobility/walking distance changed since discharge?	199 (30%)	291 (46%)
Mobility/walking distance improved	401 (61%)	244 (39%)
Mobility/walking remained about the same	54 (8.2%)	92 (15%)
Mobility/walking distance deteriorated	6 (0.9%)	3 (0.5%)
Not able to evaluate	1 (0.2%)	-
Missing		
Capacity to prepare meals alone? (Yes)	401 (61%)	401 (64%)
Weight trend over the last few days/weeks*		
Increased	33 (10%)	69 (22%)
Reduced	61 (18%)	75 (24%)
Stable	170 (52%)	134 (43%)
Unknown	50 (15%)	24 (7.7%)
Missing	16 (4.8%)	11 (3.5%)
Glucose values are most often**		
Between 4 and 10	115 (60%)	121 (67%)
> 10	17 (8.9%)	13 (7.2%)
Between 10 and 15	8 (4.2%)	6 (3.3%)
> 15	3 (1.6%)	2 (1.1%)
Unknown	44 (23%)	29 (16%)
Missing	5 (2.6%)	9 (5.0%)
Do you take your medication as prescribed?		
No or not quite	20 (3.0%)	19 (3.0%)
Yes	640 (97%)	609 (97%)
Missing	1 (0.2%)	2 (0.3%)
Is there any medication discrepancy between your current and discharge lists?	466 (70%)	367 (58%)
No	192 (29%)	259 (41%)
Yes	3 (0.5%)	4 (0.6%)
Missing		
Are you taking any of these medications? (Yes)		
Anticoagulants	205 (31%)	208 (33%)
Narcotics and opiates	128 (19%)	124 (20%)
Sedatives	162 (25%)	157 (25%)
Insulin	97 (15%)	90 (14%)
Have you experienced any of these adverse events since discharge? (Yes)	18 (2.7%)	23 (3.7%)
Dizziness	3 (0.5%)	4 (0.6%)
Bleeding	1 (0.2%)	5 (0.8%)

© 2023 Donzé J et al. *JAMA Internal Medicine.*

Hypoglycemia	0 (0.0%)	2 (0.3%)
Delirium	10 (1.5%)	8 (1.3%)
Lethargy/oversedation	21 (3.2%)	37 (5.9%)
Nausea/vomiting	3 (0.5%)	8 (1.3%)
Falls	409 (62%)	364 (58%)
None		
Have you consulted with your primary care physician since your discharge?	305 (46%)	70 (11%)
No	278 (42%)	269 (43%)
Yes, as planned	78 (12%)	289 (46%)
Yes, as a new appointment	-	2 (0.3%)
Missing		
Why have you not yet consulted with any treating physician since your discharge?	235 (77%)	19 (27%)
Visit is planned	10 (3.3%)	6 (8.6%)
Patient did not want to or could not	20 (6.6%)	14 (20%)
Treating physician was not available	37 (12%)	31 (44%)
Other	3 (1.0%)	0 (0.0%)
Unknown		
This follow-up call suggested that seeing a treating physician was necessary	206 (31%)	183 (29%)
Patient education about their diseases was refreshed	646 (98%)	617 (98%)

*Only applicable if the patient had heart failure, liver disorder, chronic renal failure, or liver cirrhosis.
**Only applicable if the patient had diabetes.

© 2023 Donzé J et al. *JAMA Internal Medicine*.

eTable 5: Secondary analysis based on per-protocol dataset at 30 days post-discharge from the index hospital admission. Binary outcomes are presented with risks and risk difference (in days to primary outcome) with the restricted mean survival time (RMST) truncated at 30 days and count outcomes with incidence (per 30 patient-days) and incidence rate ratio.

	**Intervention group (N = 631)**	**Control group (N = 645)**		***P*-value**
Primary outcome	n/N (%, 95% CI)		Risk difference (95% CI)[a]	
Unplanned readmission or death	134/608 (22%, 19 to 26%)	123/635 (19%, 16 to 23%)	2.6% (-1.9 to 7.1%)	0.25
Secondary outcomes				
Death	28/613 (4.6%, 3.2 to 6.5%)	15/637 (2.4%, 1.4 to 3.8%)	2.1% (0.1 to 4.2%)	0.037
Death without unplanned readmission	12/605 (2.0%, 1.2 to 3.6%)	9/635 (1.4%, 0.7 to 2.7%)	0.6% (-0.8 to 2.1%)	0.39
Unplanned readmission	119/605 (20%, 17 to 23%)	114/635 (18%, 15 to 21%)	1.9% (-2.4 to 6.2%)	0.38
	RMST (95% CI), days		RMST difference (95% CI)[b]	
	N = 608	N = 635		
Time to unplanned readmission or death	26.5 (25.9 to 27.1)	27.0 (26.4 to 27.5)	-0.48 (-1.22 to 0.25)	0.20
	n/N (incidence per 30 days, 95% CI)		Incidence rate ratio (95% CI)[c]	
Health care utilization				
Number of unplanned hospitalization days	1041 (1.53, 1.43 to 1.62)	1071 (1.45, 1.37 to 1.54)	0.84 (0.58 to 1.21)	0.35
Number of planned hospitalization days	124 (0.18, 0.15 to 0.22)	158 (0.22, 0.18 to 0.25)	0.66 (0.31 to 1.42)	0.29
Number of unplanned hospital readmissions	127/605 (0.19, 0.16 to 0.22)	130/635 (0.18, 0.15 to 0.21)	1.05 (0.82 to 1.35)	0.68
Number of planned hospital readmissions	26/603 (0.04, 0.02 to 0.06)	32/632 (0.04, 0.03 to 0.06)	0.89 (0.52 to 1.53)	0.68
Number of emergency room visits	47/603 (0.07, 0.05 to 0.09)	50/630 (0.07, 0.05 to 0.09)	0.97 (0.62 to 1.53)	0.90
Number of primary care provider consultations	1020/587 (1.53, 1.44 to 1.63)	1091/624 (1.53, 1.44 to 1.62)	1.00 (0.92 to 1.10)	0.92
Patient satisfaction	n/N (%, 95% CI)		Risk difference (95% CI)[a]	
Satisfied with quality of their care transition (3 items on the CTM-3)	412/491 (84%, 80 to 87%)	456/531 (86%, 83 to 89%)	-1.6% (-5.9 to 2.7%)	0.46
Cost of readmission	**Intervention group (N = 127)**	**Control group (N = 124)**		
	Mean (SD), Swiss francs		Mean difference (95% CI)[d]	
Costs of readmission	15,008 (14,688)	15,712 (16,729)	-1,226 (-5,587 to 3,135)	0.58

a) A negative difference would indicate a positive intervention effect; b) restricted mean survival time (RMST) truncated at 30 days, a positive difference would indicate a positive intervention effect; c) an incidence rate ratio smaller than 1 would indicate a positive intervention effect; d) a negative mean difference would indicate a positive intervention effect.

© 2023 Donzé J et al. *JAMA Internal Medicine*.

eTable 6 Stratum-specific analysis of the primary outcome (unplanned readmission or death within 30 days of discharge) and unplanned readmission within 30 days of discharge, together with its competing event (death without prior readmission). This analysis was included because there was evidence that the homogeneity assumption might not hold for these outcomes (i.e., that the intervention effect depended on the stratum). A negative risk difference would indicate a positive intervention effect.

	Intervention group (N = 692) n (%, 95% CI)	Control group (N = 694) n (%, 95% CI)	Risk difference (95% CI)	*P*-value
Unplanned readmission or death				
**HOSPITAL score 4–5**				
Center 1	29/136 (21%, 15 to 29%)	17/135 (13%, 7.9 to 19%)	8.7% (-0.1 to 17.6%)	0.06
Center 2	15/104 (14%, 8.8 to 23%)	22/104 (21%, 14 to 30%)	-6.7% (-17.1 to 3.6%)	0.20
Center 3	15/103 (15%, 8.9 to 23%)	11/104 (11%, 5.9 to 18%)	4.0% (-5.0 to 13.0%)	0.39
Center 4	20/129 (16%, 10 to 23%)	32/130 (25%, 18 to 33%)	-9.1% (-18.8 to 0.6%)	0.07
**HOSPITAL score ≥ 6**				
Center 1	17/61 (28%, 18 to 41%)	15/62 (24%, 15 to 37%)	3.7% (-11.8 to 19.2%)	0.64
Center 2	13/45 (29%, 17 to 44%)	5/43 (12%, 4.8 to 26%)	17.3% (0.9 to 33.6%)	0.045
Center 3	14/45 (31%, 19 to 46%)	14/45 (31%, 19 to 46%)	0.0% (-19.1 to 19.1%)	1.00
Center 4	22/69 (32%, 22 to 44%)	18/71 (25%, 16 to 7%)	6.5% (-8.4 to 21.5%)	0.39
Unplanned readmission				
**HOSPITAL score 4–5**				
Center 1	22/136 (17%, 12 to 25%)	17/135 (13%, 8.1 to 20%)	4.5% (-4.1 to 13%)	0.30
Center 2	14/104 (15%, 9.1 to 24%)	20/104 (19%, 13 to 29%)	-4.7% (-15 to 5.7%)	0.37
Center 3	12/103 (12%, 7.3 to 21%)	10/104 (10%, 5.3 to 17%)	2.8% (-5.8 to 11%)	0.52
Center 4	18/129 (14%, 9.4 to 22%)	31/130 (25%, 18 to 34%)	-10% (-20 to -0.7%)	0.036
**HOSPITAL score ≥ 6**				
Center 1	14/61 (24%, 15 to 38%)	14/62 (24%, 15 to 38%)	0.2% (-15 to 16%)	0.98
Center 2	12/45 (28%, 18 to 45%)	4/43 (9.2%, 3.6 to 23%)	19% (3.1 to 35%)	0.020
Center 3	13/45 (30%, 19 to 47%)	10/45 (22%, 13 to 38%)	7.8% (-10 to 26%)	0.40
Center 4	22/69 (33%, 23 to 46%)	18/71 (25%, 17 to 37%)	7.9% (-7.0 to 23%)	0.30
Death without unplanned readmission				
**HOSPITAL score 4–5**				
Center 1	7/136 (3.5%, 1.6 to 7.6%)	0/135 (1.9%, 0.8 to 4.6%)	1.6% (-0.6 to 3.8%)	0.15
Center 2	1/104 (2.0%, 0.6 to 6.4%)	2/104 (1.0%, 0.3 to 3.5%)	1.0% (-0.5 to 2.6%)	0.20
Center 3	3/103 (2.6%, 1.0 to 7.2%)	1/104 (1.4%, 0.5 to 4.1%)	1.2% (-0.6 to 3.1%)	0.19
Center 4	2/129 (1.6%, 0.5 to 5.1%)	1/130 (0.8%, 0.2 to 2.7%)	0.8% (-0.4 to 2.1%)	0.18
**HOSPITAL score ≥ 6**				
Center 1	3/61 (4.5%, 1.7 to 12%)	1/62 (2.4%, 0.8 to 7.1%)	2.2% (-1.0 to 5.3%)	0.18
Center 2	1/45 (2.9%, 0.7 to 12%)	1/43 (1.8%, 0.4 to 7.5%)	1.2% (-1.2 to 3.5%)	0.33
Center 3	1/45 (7.2%, 3.0 to 18%)	4/45 (4.1%, 1.5 to 11%)	3.2% (-1.6 to 7.9%)	0.20

© 2023 Donzé J et al. *JAMA Internal Medicine.*

Center 4	0/69 (0.0%, 0.0 to 0.0%)	0/71 (0.0%, 0.0 to 0.0%)	0.0% (-0.0 to 0.0%)	1.00

eTable 7: Strata-specific analysis of the primary outcome (unplanned readmission or death within 30 days of discharge) and unplanned readmission within 30 days of discharge, together with its competing event (death without prior readmission), and based on the per-protocol dataset. This analysis was included because there was evidence that the homogeneity assumption might not hold for these outcomes (i.e., that the intervention effect depended on the stratum). A negative risk difference would indicate a positive intervention effect.

	Intervention group (N = 608) n (%, 95% CI)	Control group (N = 635) n (%, 95% CI)	Risk difference (95% CI)	*P*-value
Unplanned readmission or death				
**HOSPITAL score 4–5**				
Center 1	26/125 (21%, 15 to 29%)	14/122 (11%, 6.9 to 19%)	9.3% (0.2 to 18.4%)	0.047
Center 2	15/90 (17%, 10 to 26%)	22/103 (21%, 14 to 30%)	-4.7% (-15.7 to 6.3%)	0.41
Center 3	11/76 (14%, 8.1 to 24%)	10/88 (11%, 6.2 to 20%)	3.1% (-7.2 to 13.4%)	0.55
Center 4	17/117 (15%, 9.2 to 22%)	29/117 (25%, 18 to 33%)	-10.3% (-20.4 to -0.2%)	0.048
**HOSPITAL score ≥ 6**				
Center 1	17/57 (30%, 19 to 43%)	15/56 (27%, 17 to 40%)	3.0% (-13.6 to 19.6%)	0.72
Center 2	13/43 (30%, 18 to 46%)	5/42 (12%, 4.9 to 26%)	18.3% (1.5 to 35.2%)	0.039
Center 3	13/35 (37%, 22 to 55%)	10/38 (26%, 14 to 43%)	10.8% (-10.4 to 32.1%)	0.32
Center 4	22/65 (34%, 23 to 46%)	18/69 (26%, 17 to 38%)	7.8% (-7.7 to 23.2%)	0.33
Unplanned readmission				
**HOSPITAL score 4–5**				
Center 1	19/123 (16%, 10 to 24%)	14/122 (11%, 7.0 to 19%)	4.2% (-4.3 to 13%)	0.33
Center 2	14/90 (16%, 10 to 25%)	20/103 (20%, 13 to 29%)	-3.9% (-15 to 6.9%)	0.48
Center 3	10/76 (14%, 7.6 to 24%)	9/88 (10%, 5.5 to 19%)	3.3% (-6.7 to 13%)	0.51
Center 4	16/117 (14%, 8.7 to 22%)	28/117 (24%, 18 to 33%)	-11% (-20 to -0.6%)	0.038
**HOSPITAL score ≥ 6**				
Center 1	14/57 (25%, 16 to 39%)	14/56 (24%, 15 to 38%)	0.3% (-15 to 16%)	0.97
Center 2	12/43 (28%, 18 to 46%)	4/42 (9.4%, 3.7 to 24%)	19% (2.8 to 35%)	0.021
Center 3	12/34 (36%, 23 to 57%)	7/38 (18%, 9.3 to 35%)	18% (-2.1 to 38%)	0.08
Center 4	22/65 (33%, 23 to 46%)	18/69 (26%, 17 to 38%)	7.2% (-7.9 to 22%)	0.35
Death without unplanned readmission				
**HOSPITAL score 4–5**				
Center 1	5/123 (2.4%, 1.0 to 6.2%)	0/122 (1.7%, 0.6 to 4.6%)	0.7% (-1.1 to 2.6%)	0.44
Center 2	1/90 (1.9%, 0.6 to 6.2%)	2/103 (1.3%, 0.4 to 4.3%)	0.7% (-0.9 to 2.2%)	0.39
Center 3	1/76 (1.5%, 0.4 to 6.2%)	1/88 (1.0%, 0.2 to 4.4%)	0.5% (-0.8 to 1.7%)	0.47
Center 4	1/117 (1.1%, 0.3 to 4.4%)	1/117 (0.7%, 0.1 to 2.9%)	0.4% (-0.5 to 1.3%)	0.37
**HOSPITAL score ≥ 6**				
Center 1	3/57 (4.2%, 1.5 to 12%)	1/56 (2.9%, 1.0 to 8.5%)	1.3% (-1.9 to 4.5%)	0.41
Center 2	1/43 (2.6%, 0.6 to 11%)	1/42 (2.1%, 0.5 to 9.0%)	0.5% (-1.6 to 2.7%)	0.63

© 2023 Donzé J et al. *JAMA Internal Medicine.*

Center 3	0/34 (4.6%, 1.4 to 15%)	3/38 (3.7%, 1.1 to 12%)	0.9% (-2.8 to 4.7%)	0.63
Center 4	0/65 (0.0%, 0.0 to .%)	0/69 (0.0%, 0.0 to .%)	0.0% (-0.0 to 0.0%)	1.00

© 2023 Donzé J et al. *JAMA Internal Medicine*.

eTable 8: Main diagnoses of unplanned readmissions for the entire study population and by intervention group. N (%).

	**Entire study population**		**By group**	
	Readmission n = 229	Proportion of readmissions with the same diagnosis as their index admission	Intervention n = 117	Control n = 112
Heart failure	24 (10)	9 (38)	12 (10)	12 (11)
Ischemia	1 (0)	1 (100)	-	1 (1)
Rhythm	4 (2)	-	3 (3)	1 (1)
Thromboembolism	2 (1)	-	2 (2)	-
Stroke	1 (0)	1 (100)	1 (1)	-
COPD decompensate	10 (4)	8 (80)	7 (6)	3 (3)
Pneumonia	19 (8)	5 (26)	10 (9)	9 (8)
Infection	37 (16)	10 (27)	16 (14)	21 (19)
Gastro	14 (6)	4 (29)	10 (9)	4 (4)
Hepatology	5 (2)	2 (40)	2 (2)	3 (3)
Nephrology	5 (2)	1 (20)	2 (2)	3 (3)
Metabolic	4 (2)	1 (25)	1 (1)	3 (3)
Drug side effect	2 (1)	1 (50)	2 (2)	-
Oncology	49 (21)	23 (47)	23 (20)	26 (23)
Epilepsy	2 (1)	-	1 (1)	1 (1)
Other	50 (22)	10 (20)	25 (21)	25 (22)

251 readmissions in table; 231 in database; info only available for 229 patients

© 2023 Donzé J et al. *JAMA Internal Medicine.*

eTable 9: Sensitivity analyses; primary outcome (unplanned readmission or death within 30 days of discharge) excluding early readmissions or deaths (i.e., patients who died or were readmitted within 24 hours of the index discharge).

	**Intervention group (N = 681)**	**Control group (N = 688)**	**Risk difference (95% CI)[a]**	***P*-value**
Primary outcome	n (%, 95% CI)			
Unplanned readmission or death	134 (20%, 17 to 23%)	128 (19%, 16 to 22%)	1.1% (-3.0 to 5.3%)	0.60

a) A negative difference would indicate a positive intervention effect

© 2023 Donzé J et al. *JAMA Internal Medicine*.

eTable 10: Sensitivity analyses. All outcomes are without adjusting for the stratification factors (crude analysis). Binary outcomes are presented with risks and the risk difference (in days to primary outcome) with the restricted mean survival time (RMST) truncated at 30 days. Count outcomes are presented with incidence (per 30 patient-days) and incidence rate ratio, using the same methods as for the primary analysis, i.e., negative binomial regression with robust standard errors and adjustment for zero-inflation in unplanned hospitalization days.

	**Intervention group (N = 692)**	**Control group (N = 694)**		***P*-value**
	n (%, 95% CI)		Risk difference (95% CI)[a]	
Unplanned readmission or death	145 (21%, 18 to 24%)	134 (19%, 16 to 23%)	1.6% (-2.6 to 5.9%)	0.44
Death	32 (4.6%, 3.3 to 6.5%)	18 (2.4%, 1.4 to 3.8%)	2.0% (0.05 to 4.1%)	0.043
Unplanned readmission	127 (19%, 17 to 23%)	124 (18%, 15 to 21%)	1.3% (-2.9 to 5.4%)	0.55
Death without unplanned readmission	18 (2.7%, 1.7 to 4.3%)	10 (1.5%, 0.8 to 2.7%)	1.3% (-0.3 to 2.8%)	0.10
Satisfied with quality of their care transition (3 items on the CTM-3)	575 (83%, 80 to 86%)	585 (84%, 81 to 87%)	-1.2% (-5.6 to 3.2%)	0.54
	RMST (95% CI), days		RMST difference (95% CI)[b]	
Time to unplanned readmission or death	26.8 (26.3 to 27.3)	27.0 (26.5 to 27.6)	-0.24 (-0.95 to 0.48)	0.51
	n (incidence per 30 days, 95% CI)		Incidence rate ratio (95% CI)[c]	
Number of unplanned hospitalization days	1118 (1.50, 1.42 to 1.59)	1191 (1.51, 1.42 to 1.60)	0.82 (0.56 to 1.20)	0.30
Number of planned hospitalization days	136 (0.18, 0.15 to 0.22)	202 (0.26, 0.22 to 0.29)	0.78 (0.36 to 1.67)	0.52
Number of unplanned hospital readmissions	135 (0.18, 0.15 to 0.21)	140 (0.18, 0.15 to 0.21)	1.02 (0.80 to 1.30)	0.85
Number of planned hospital readmissions	28 (0.04, 0.03 to 0.05)	36 (0.05, 0.03 to 0.06)	0.82 (0.49 to 1.38)	0.46
Number of emergency department visits	51 (0.07, 0.05 to 0.09)	55 (0.07, 0.05 to 0.09)	0.97 (0.64 to 1.47)	0.88
Number of primary care provider consultations	1103 (1.52, 1.43 to 1.61)	1158 (1.51, 1.43 to 1.60)	1.00 (0.92 to 1.10)	0.94

a) A negative difference would indicate a positive intervention effect; b) restricted mean survival time (RMST) truncated at 30 days, a positive difference would indicate a positive intervention effect; c) an incidence rate ratio smaller than one would indicate a positive intervention effect.

© 2023 Donzé J et al. *JAMA Internal Medicine*.

eTable 11: Sensitivity analysis of the primary outcome (unplanned readmission or death within 30 days of discharge) using survival methods, i.e., flexible parametric survival models (parametric) or the Kaplan–Meier estimator (non-parametric) at 30 days.

	**Intervention group (N = 692)**	**Control group (N = 694)**		***P*-value**
	n (%, 95% CI)		Risk difference (95% CI)[a]	
Non-parametric	145 (22%, 19 to 25%)	134 (19%, 17 to 22%)	2.6% (-1.7 to 7.0%)	0.23
Parametric	145 (22%, 19 to 25%)	134 (20%, 17 to 23%)	2.5% (-1.8 to 6.8%)	0.25

a) A negative difference would indicate a positive intervention effect.

© 2023 Donzé J et al. *JAMA Internal Medicine*.

eTable 12: Sensitivity analysis of patient satisfaction with the quality of their care transition at 30 days, assuming a negative response to all the questions on the CTM-3 for patient who died and using multiple imputed data or complete cases only.

	**Intervention group (N = 692)**	**Control group (N = 694)**		**P-value**
	n/N (%, 95% CI)		Risk difference (95% CI)[a]	
Multiple imputed data	549/692 (79%, 76 to 83%)	569/694 (82%, 79 to 85%)	-2.8% (-7.2 to 1.7%)	0.22
Complete cases	453/568 (80%, 76 to 83%)	481/581 (83%, 80 to 86%)	-2.8% (-7.2 to 1.6%)	0.21

a) A positive risk difference would indicate a positive intervention effect.

© 2023 Donzé J et al. *JAMA Internal Medicine*.

eTable 13: Sensitivity analysis for readmission costs for patients who were readmtted, based on multiple imputed data. The mean ratio was calculated from a gamma regression. A mean ratio smaller than one would indicate a positive intervention effect.

	**Intervention group (N = 127)**	**Control group (N = 124)**		***P*-value**
	Mean (SD), Swiss francs		Gamma regression Mean ratio (95% CI)	
Multiple imputed data	15,355 (20,723)	15,921 (24,391)	0.92 (0.72 to 1.18	0.53

© 2023 Donzé J et al. *JAMA Internal Medicine*.

eTable 14: Sensitivity analysis for unplanned readmission and death, excluding patients living in nursing homes.

	**Intervention group (N = 610)**	**Control group (N = 631)**		***P*-value**
	n/N (%, 95% CI)		Risk difference (95% CI)[a]	
Unplanned readmission	115 (20%, 17 to 24%)	117 (19%, 16 to 22%)	1.3% (-3.1 to 5.7%)	0.57
Death	18 (3.0%, 1.9 to 4.6%)	13 (2.1%, 1.2 to 3.5%)	0.9% (-0.9 to 2.6%)	0.33

a) A positive risk difference would indicate a positive intervention effect.

© 2023 Donzé J et al. *JAMA Internal Medicine*.
