# Supplementary material for: Effects of a Multimodal Transitional Care Intervention in Patients at High Risk of Readmission: The TARGET-READ Randomized Clinical Trial
Source: JAMA Intern Med. 2023 May 1;183(7):658–68. doi: 10.1001/jamainternmed.2023.0791 (PMC10152373; doi:10.1001/jamainternmed.2023.0791)
Supplement: Supplement 3. — Data Sharing Statement [file jamainternmed-e230791-s003.pdf]

Data Sharing Statement

Donz�. Effects of a Multimodal Transitional Care Intervention in Patients at High Risk of Readmission. *JAMA Intern Med*. Published May 01, 2023.
doi:10.1001/jamainternmed.2023.0791

Data
Data available: No
